# Discovery of Clinical Candidate GLPG3970: A Potent and Selective Dual SIK2/SIK3 Inhibitor for the Treatment of Autoimmune and Inflammatory Diseases

**DOI:** 10.1021/acs.jmedchem.3c02246

**Published:** 2024-03-29

**Authors:** Christophe Peixoto, Agnes Joncour, Taouès Temal-Laib, Amynata Tirera, Aurélie Dos Santos, Hélène Jary, Denis Bucher, Wendy Laenen, Anna Pereira Fernandes, Stephanie Lavazais, Carole Delachaume, Didier Merciris, Corinne Saccomani, Michael Drennan, Miriam López-Ramos, Emanuelle Wakselman, Sonia Dupont, Monica Borgonovi, Carlos Roca Magadan, Alain Monjardet, Reginald Brys, Steve De Vos, Martin Andrews, Juan-Miguel Jimenez, David Amantini, Nicolas Desroy

**Affiliations:** † Galapagos SASU, 93230 Romainville, France; ‡ Galapagos NV, 2800 Mechelen, Belgium

## Abstract

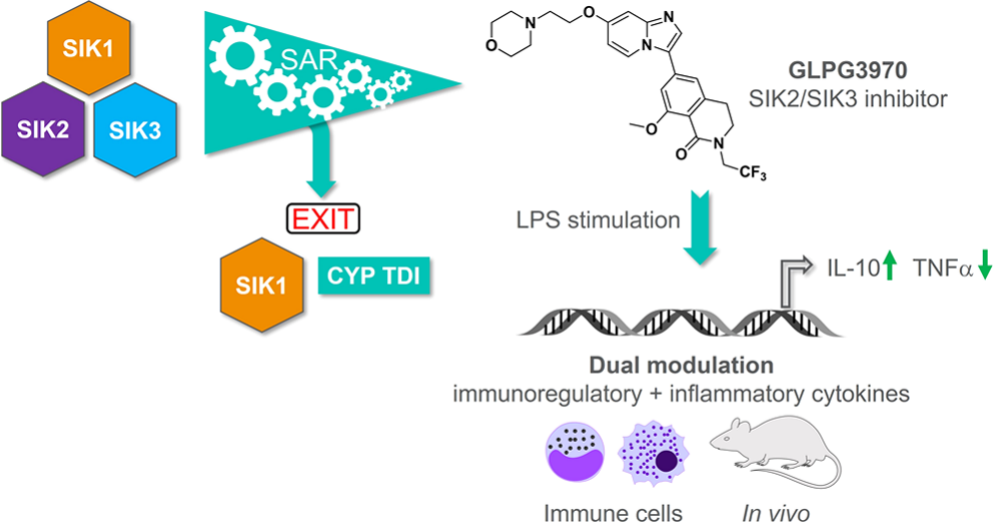

The salt-inducible kinases (SIKs) SIK1, SIK2, and SIK3 belong to the adenosine monophosphate-activated protein kinase (AMPK) family of serine/threonine kinases. SIK inhibition represents a new therapeutic approach modulating pro-inflammatory and immunoregulatory pathways that holds potential for the treatment of inflammatory diseases. Here, we describe the identification of GLPG3970 (**32**), a first-in-class dual SIK2/SIK3 inhibitor with selectivity against SIK1 (IC_50_ of 282.8 nM on SIK1, 7.8 nM on SIK2 and 3.8 nM on SIK3). We outline efforts made to increase selectivity against SIK1 and improve CYP time-dependent inhibition properties through the structure–activity relationship. The dual activity of **32** in modulating the pro-inflammatory cytokine TNFα and the immunoregulatory cytokine IL-10 is demonstrated *in vitro* in human primary myeloid cells and human whole blood, and *in vivo* in mice stimulated with lipopolysaccharide. Compound **32** shows dose-dependent activity in disease-relevant mouse pharmacological models.

## Introduction

Salt-inducible kinases (SIKs) belong to the adenosine monophosphate-activated protein kinase (AMPK) family of serine/threonine kinases.^1^ The first member of the SIK family (SIK1) was identified in 1999 when rats fed on a high-salt diet were found to induce a protein kinase in adrenal cortical tissue.^2^ Later, two other protein kinases with closely related catalytic domains were identified and are now called SIK2 and SIK3. The three SIK isoforms have very closely related kinase domain structures, making identification of selective compounds highly challenging, and are expressed broadly in tissues. The mRNA encoding SIK1 is regulated by multiple stimuli, including high dietary salt intake, adrenocorticotropic hormone signaling, glucagon signaling, circadian rhythms, and membrane depolarization. In contrast, the expression of SIK2 and SIK3 is constitutive in tissues, and little knowledge is available about ligands that increase or decrease their level of expression.

**Figure 1:**
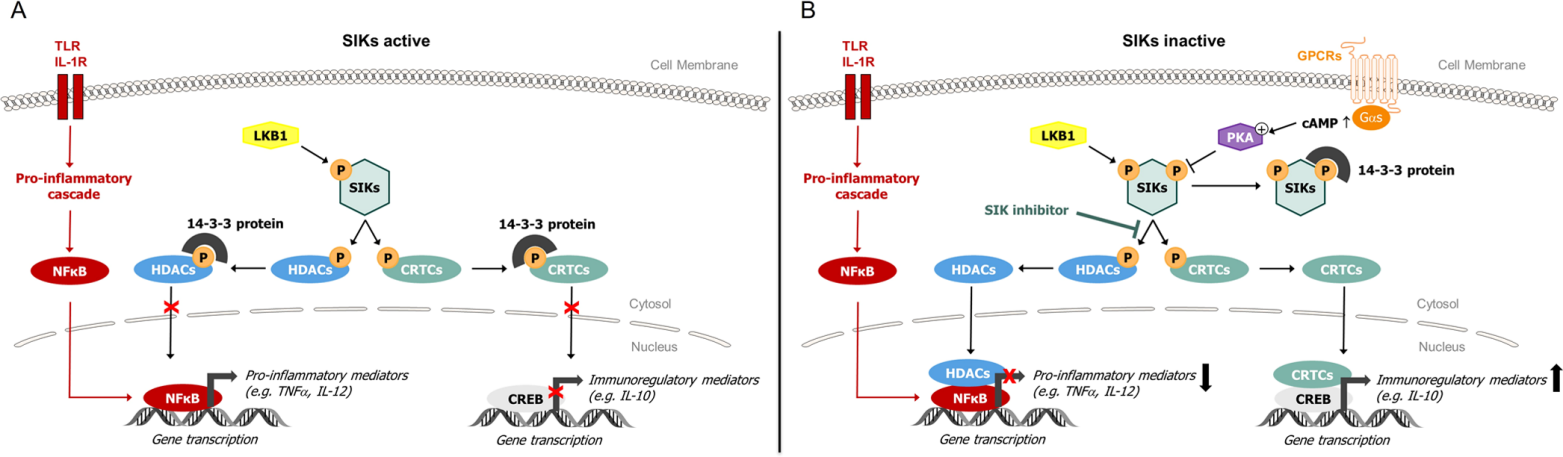
Signaling pathway of SIKs. (A) SIKs are activated. (B) SIKs are inactivated. Abbreviations: cAMP, cyclic adenosine monophosphate; CREB, cAMP-response element binding protein; CRTC, CREB-regulated transcription coactivators; GPCR, G protein coupled receptor; HDAC, histone deacetylases; IL-10, interleukin-10; IL-12, interleukin-12; IL-1R, interleukin-1 receptor; LKB1, liver kinase B1; NFκB, nuclear factor kappa B; PKA, protein kinase A; SIK, salt-inducible kinases; TLR, Toll-like receptor; TNFα, tumor necrosis factor alpha.

Activity of SIKs is induced by phosphorylation of their activation loop by the liver kinase B1 (LKB1) and is inhibited by cyclic adenosine monophosphate (cAMP)/protein kinase A (PKA)-dependent phosphorylation leading to increased binding to 14-3-3 proteins (Figure 1). Two important groups of substrates for the SIKs have been identified, namely the cAMP-response element binding protein (CREB)-regulated transcriptional coactivators (CRTCs: CRTC1, CRTC2, and CRTC3) and the Class 2a histone deacetylases (HDACs: HDAC4, HDAC5, HDAC7, and HDAC9). Phosphorylation of CRTC and HDAC family members by SIKs induces their binding to 14-3-3 proteins and retention in the cytosol. The inhibition of SIKs, either by cAMP/PKA pathway-dependent phosphorylation or by direct pharmacological inhibition, allows the dephosphorylation of CRTCs and HDACs, and their respective translocation to the nucleus, where they promote or repress gene transcription, respectively.^3^

The effect of SIK inhibition on immune responses has been explored in innate and adaptive immune cells. Investigation of pan-SIK inhibitors in mouse bone marrow-derived dendritic cells (BMDCs) stimulated with zymosan has demonstrated increased production of the immunoregulatory cytokines interleukin-10 (IL-10) associated with decreased secretion of the pro-inflammatory cytokines tumor necrosis factor alpha (TNFα), IL-12, IL-1β, and IL-6.^4^ Furthermore, SIK inhibition in human macrophages stimulated with lipopolysaccharide (LPS) or IL-1β markedly reduced the phosphorylation of CRTC3 and HDAC4 substrates, resulting in modulation of pro- and anti-inflammatory cytokines.^5^ SIK inhibition during mouse macrophage differentiation induced a phenotypic switch to regulatory macrophages characterized by a reduced capacity to produce pro-inflammatory cytokines coupled with enhanced IL-10 production upon stimulation with LPS.^6^

SIK inhibition represents a promising novel approach for the treatment of inflammatory diseases, specifically for those that are characterized by an imbalance of the immune system such as inflammatory bowel disease (IBD), where a dysregulation of pro- and anti-inflammatory pathways in intestinal tissues defines the disease pathogenesis.^7^

The enzymatic activity of endogenous SIK isoforms was determined following immunoprecipitation from mouse fetal liver-derived macrophages (FLDMs) and bone marrow-derived macrophages (BMDMs); SIK2 showed higher enzymatic activity than SIK3, and SIK1 had the lowest contribution. Investigation of the role of SIK isoforms using catalytically inactive mutants and combination with pan-SIK inhibitors led to the conclusion that all three isoforms, in particular SIK2 and SIK3, contribute toward the overall phenotype of macrophages. SIK2 and SIK3 were also found to be essential for the secretion of inflammatory mediators in mouse bone marrow-derived mast cells (BMMCs) stimulated with IL-33.^8^ Exploration of the role of SIK isoforms on T cell development in mice suggested that SIK2 and SIK3 are the major functional isoforms in T cells, with combined knockout of SIK2 and SIK3 resulting in a severe block in thymic T cell development, consistent with a defect in the positive or negative selection of double-positive thymocytes.^9^ Consequently, SIK1 appears to have a minor contribution to the activity in immune cells. In contrast, SIK1 was reported to play a key role in blood pressure regulation and vascular remodeling.^10−13^ Dual SIK2/SIK3 inhibition with selectivity against SIK1 could therefore offer a better safety profile with a lower impact on the cardiovascular system than pan-SIK inhibition, while retaining the desired activity on the immune system.

**Figure 2:**
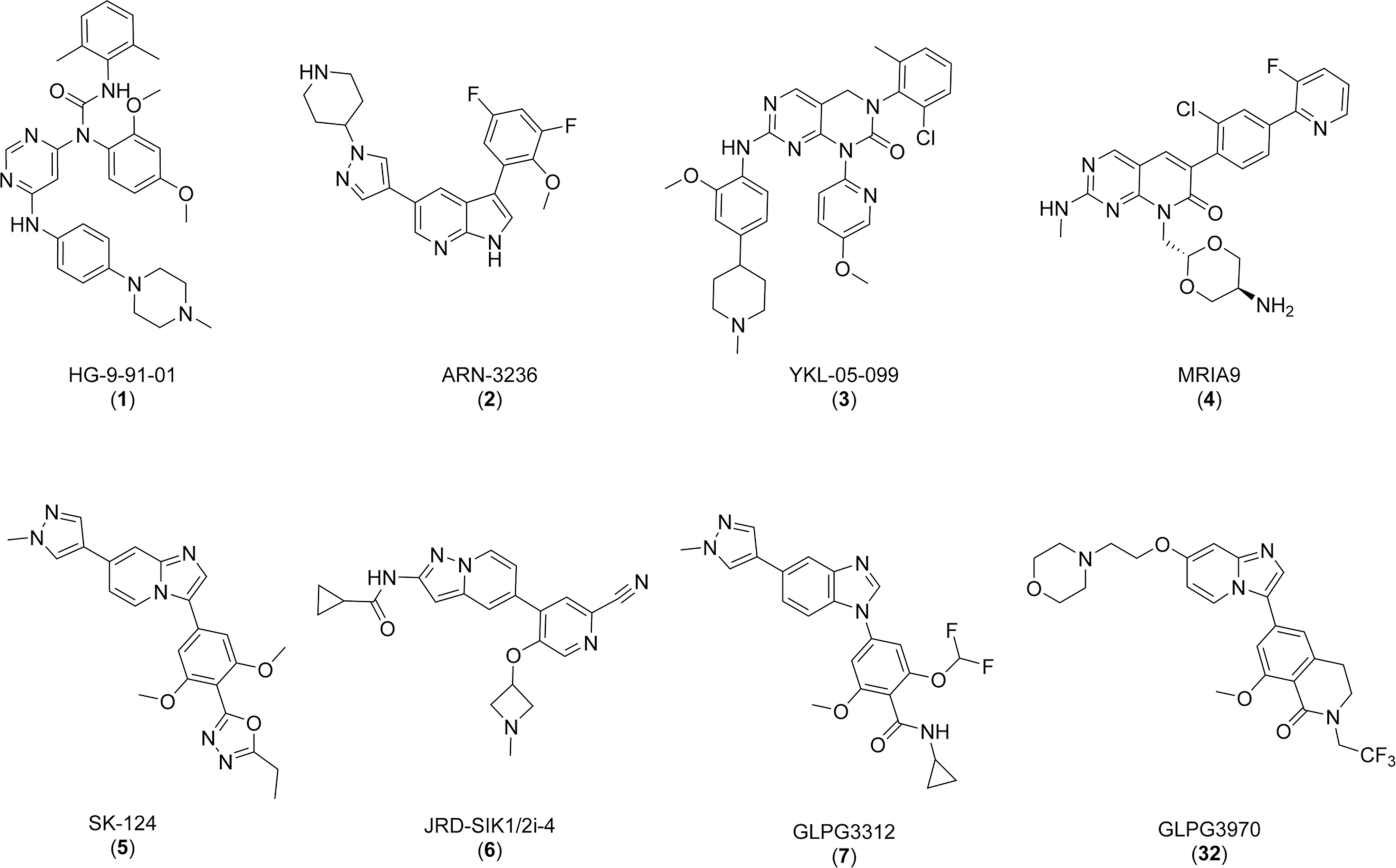
Structures of selected small-molecule SIK inhibitors described in the literature and of GLPG3970 (**32**).^4,5,14−20^

An increasing number of chemotypes inhibiting the kinase activity of SIKs have been described in the literature. Examples of small-molecule SIK inhibitors are shown in Figure 2, and their associated potency on SIKs is reported in Table 1. Improvement of selectivity against other kinases and between SIK isoforms is a key objective in the development of new SIK inhibitors to provide tools for interrogating the pharmacology of SIKs. We previously disclosed the identification of the potent and selective pan-SIK inhibitor GLPG3312 (**7**), a type 1 kinase inhibitor, and the first human SIK3 crystal structure.^14^ In parallel to the efforts that led to GLPG3312, we took advantage of the knowledge gained from the crystal structure of SIK3 to explore the possibility to achieve potent dual SIK2 and SIK3 inhibition with selectivity against SIK1 to avoid SIK1-related side effects mentioned above.

**Table 1:** Reported SIK Inhibitory Activity of Compounds from Figure 2

	**IC_50_ or *K*_i_, nM**		
**Compound**	**SIK1**	**SIK2**	**SIK3**
HG-9-91-01 **(1)**(15)	250	67	430
ARN-3236 **(2)**(5,16,17)	21.6	<1	6.6
YKL-05-099 **(3)**(4)	10	40	30
MRIA9 **(4)**(18)	55	48	22
SK-124 **(5)**(19)	6.5	0.4	1.2
JRD-SIK1/2i-4 **(6)**(20)	3.1	1.9	70
GLPG3312 **(7)**(14)	2.0	0.7	0.6
GLPG3970 **(32)**	282.8	7.8	3.8

Here, we describe the discovery of GLPG3970, a first-in-class dual SIK2/SIK3 inhibitor evaluated in clinical trials for the treatment of autoimmune and inflammatory diseases. Efforts to increase selectivity against SIK1 and improve CYP time-dependent inhibition (TDI) properties through modulation of structural properties will be described. The dual mechanism of GLPG3970 in modulating pro- and anti-inflammatory cytokines is demonstrated in immune-mediated *in vitro* and *in vivo* models.

## Results and Discussion

### Strategy and Hit Identification

We previously described the identification of a 4-(5-substituted-benzimidazol-1-yl)benzamide chemical series displaying pan-SIK inhibition following an HTS campaign.^14^ The first crystal structure of SIK3 in complex with compound **8** (Table 2) was elucidated, allowing a better understanding of the binding mode and of the selectivity of the chemical series.

**Table 2:**
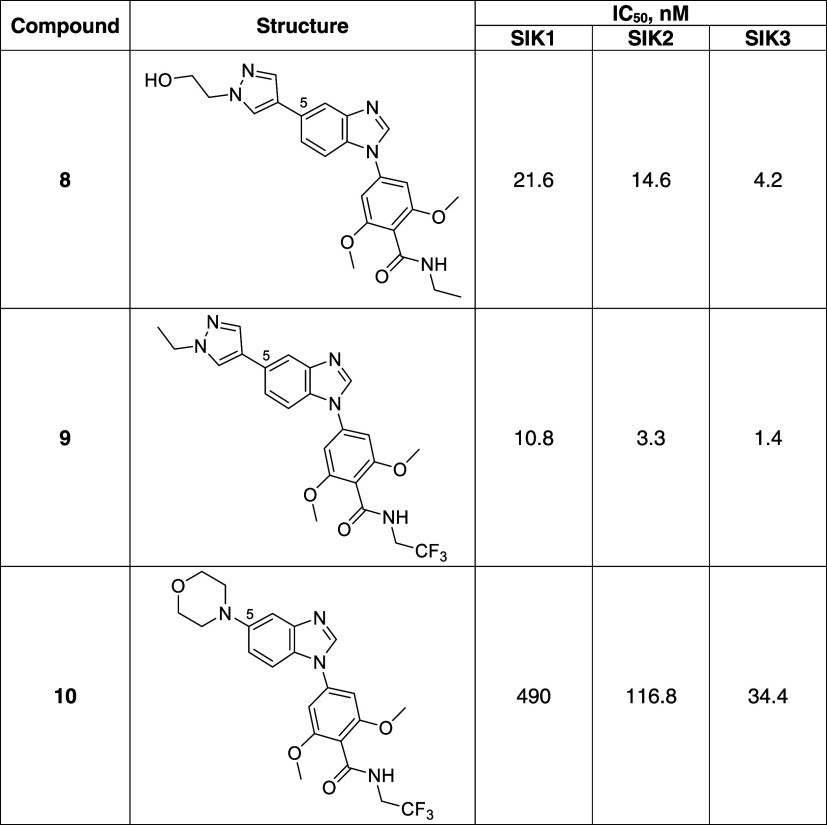
Compounds in Cocrystal Structure and Early Structure–Activity Relationship

**Figure 3:**
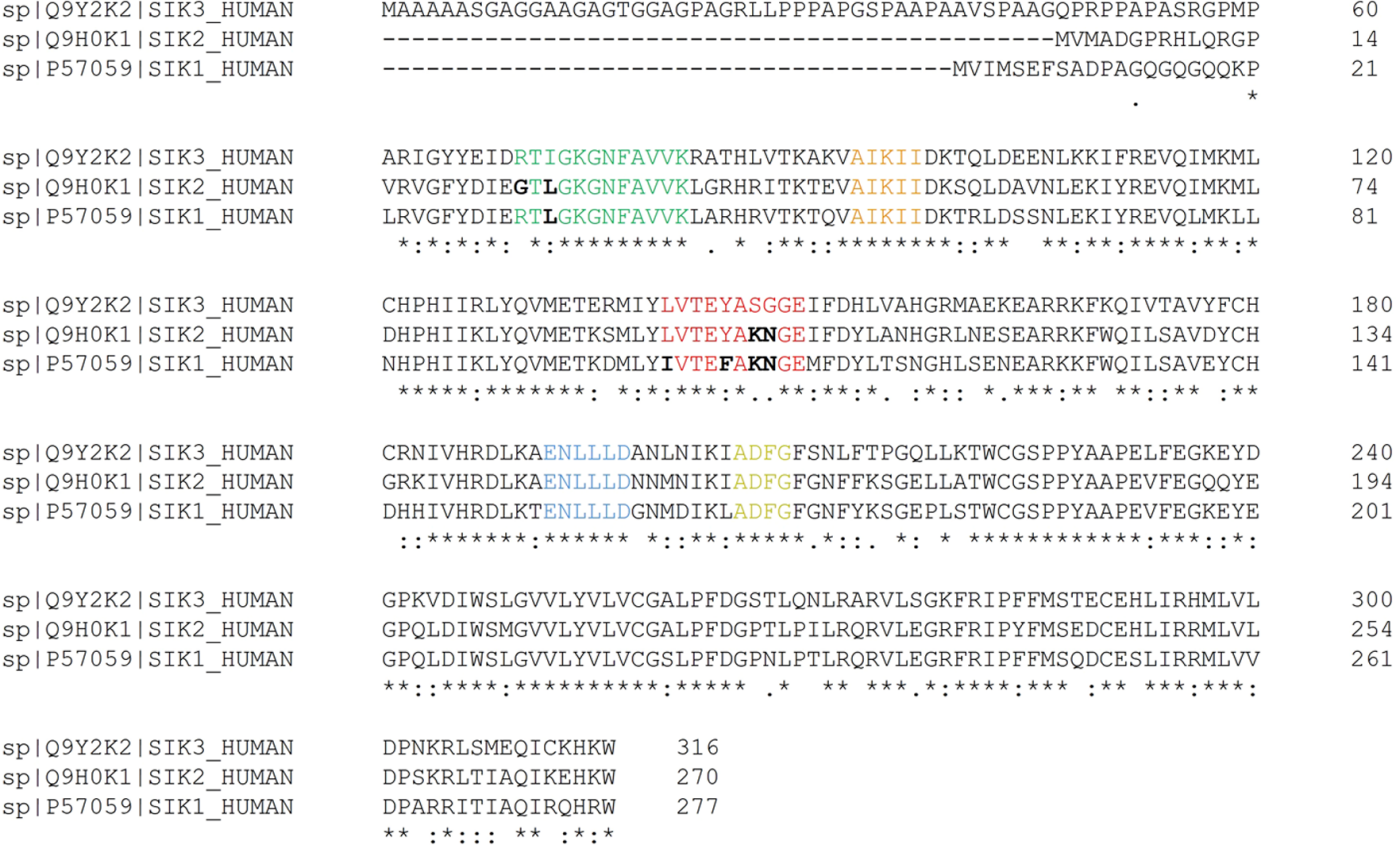
Sequence alignment between the kinase domain of SIK3 (top), SIK2 (middle), and SIK1 (bottom). The residues that form the different binding site regions are highlighted in different colors: P-loop in green, catalytic center in orange, hinge in red, bottom part in blue, and DFG motif in yellow. Additionally, non-conserved residues in these regions are indicated in black bold, showing the high similarity between the binding sites of isoforms.

**Figure 4:**
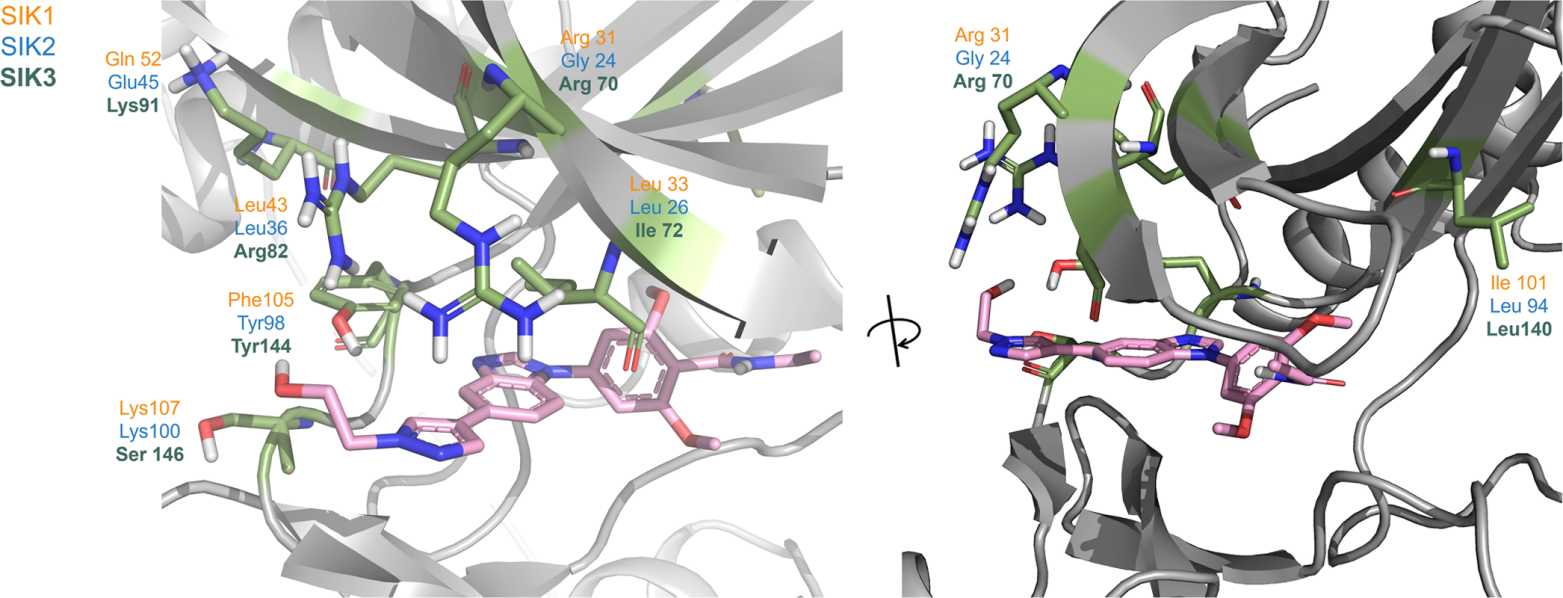
X-ray structure of SIK3 in complex with **8** (PDB: 8OKU). Non-conserved residues between SIK1, SIK2, and SIK3 that are located close to the binding site are displayed in sticks.

Sequence alignment of the three SIK isoforms showed that there are only five non-conserved residues in the ATP-binding site, pointing at the high similarity between isoforms (Figure 3). Sequence alignment and visual inspection of the ligand binding site in the X-ray structure were used to predict how different residues could have an impact on SIK selectivity (Figure 4). Most of the residues differing between the isoforms are located close to the hinge and in the top part of the pocket, while the residues in the rest of the binding site are all conserved.

Structure–activity relationship (SAR) optimization described previously led to the identification of the alkyl pyrazole and second methoxy group on the phenyl ring as important features for high potency on SIKs. As can be seen in Figure 4, residues are very similar between isoforms around the dimethoxybenzamide moiety of compound **8**. In contrast, different residues between SIK isoforms are present around the pyrazole substituent in position 5 of the benzimidazole scaffold of compound **8**. In particular, a tyrosine residue in SIK2 (Tyr98) and SIK3 (Tyr144) is replaced by a phenylalanine residue in SIK1 (Phe105). The pyrazole moiety in compound **8** is coplanar with the benzimidazole scaffold allowing a displaced π–π interaction with tyrosine or phenylalanine and a weak hydrogen bond between the slightly polarized C–H group of the pyrazole and the carbonyl moiety of alanine in the hinge, which resulted in pan-SIK inhibition. We hypothesized that exploration of structurally diverse substituents in position 5 of the benzimidazole scaffold could allow escape from flatland, achieving selectivity against SIK1 through interactions with the side chain of residues in an area of the active site where non-conserved residues between isoforms are present. For example, H-bond interaction with the tyrosine residue in SIK2 (Tyr98) and SIK3 (Tyr144) would not be feasible with the phenylalanine residue in SIK1 (Phe105). The possibility that more distal, non-conserved residues could lead to subtle differences in the conformation of the active site between isoforms and impact the binding of compounds was not excluded and could result in isoform selectivity. Therefore, a broad exploration including modification of substituents and scaffold was envisaged.

Our goal was to achieve single-digit nanomolar potency on SIK2 and SIK3 and at least 30-fold selectivity against SIK1. We decided to start from a moderately active hit compound on SIK isoforms, trying to improve potency on SIK2 and SIK3 only.

As shown in Table 2, replacement of the ethyl pyrazole moiety in position 5 of the benzimidazole scaffold in compound **9** by a non-aromatic morpholine substituent (**10**) led to at least a 25-fold drop of activity on SIKs. Interestingly, hit compound **10** displayed a trend of selectivity against SIK1.

### SAR Exploration

Starting from the moderately active hit compound **10**, scaffold hopping, replacement of morpholine, and modification of benzamide moiety were explored to improve potency on SIKs and selectivity against SIK1. Unexpectedly, scaffold hopping from benzimidazole in **10** to imidazo[1,2-*a*]pyridine in **11** resulted in increased potency on SIK2 and SIK3, and improved selectivity against SIK1 (Table 3). The bottom part of the molecule was also modified, as previously reported,^14^ by replacing one of the methyl groups on the dimethoxyphenyl portion with a difluoromethyl group and replacing the trifluoroethyl group on the carboxamide moiety with a cyclopropyl group to produce analog **12**. These changes further increased potency on SIK2 but also on SIK1. Analogs with other amine substituents on the imidazo[1,2-*a*]pyridine scaffold were tested pairwise using the bottom parts of molecules **11** and **12**. The substituents examined were difluoroazetidine (**13** and **14**) and *N*-methylpiperazine (**15** and **16**), which showed improved potency, in particular on SIK2, for the bottom part containing the difluoromethoxy group and cyclopropyl amide (Table 3). The difluoroazetidine derivative **14** exhibited greater inhibition of SIK1, SIK2, and SIK3 compared with analog **13**, as was similarly observed for the *N*-methylpiperazine derivatives **16** and **15**.

**Table 3:**
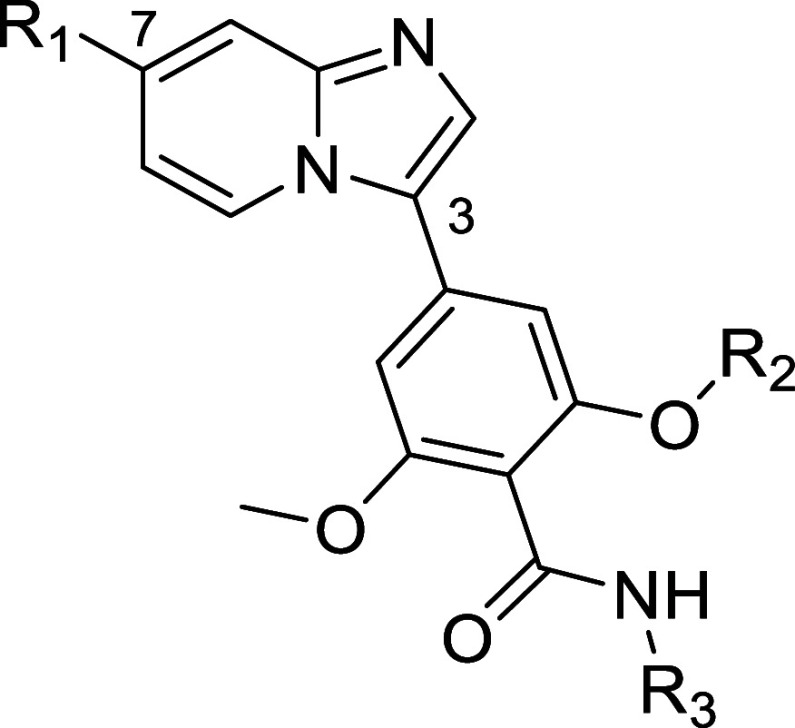

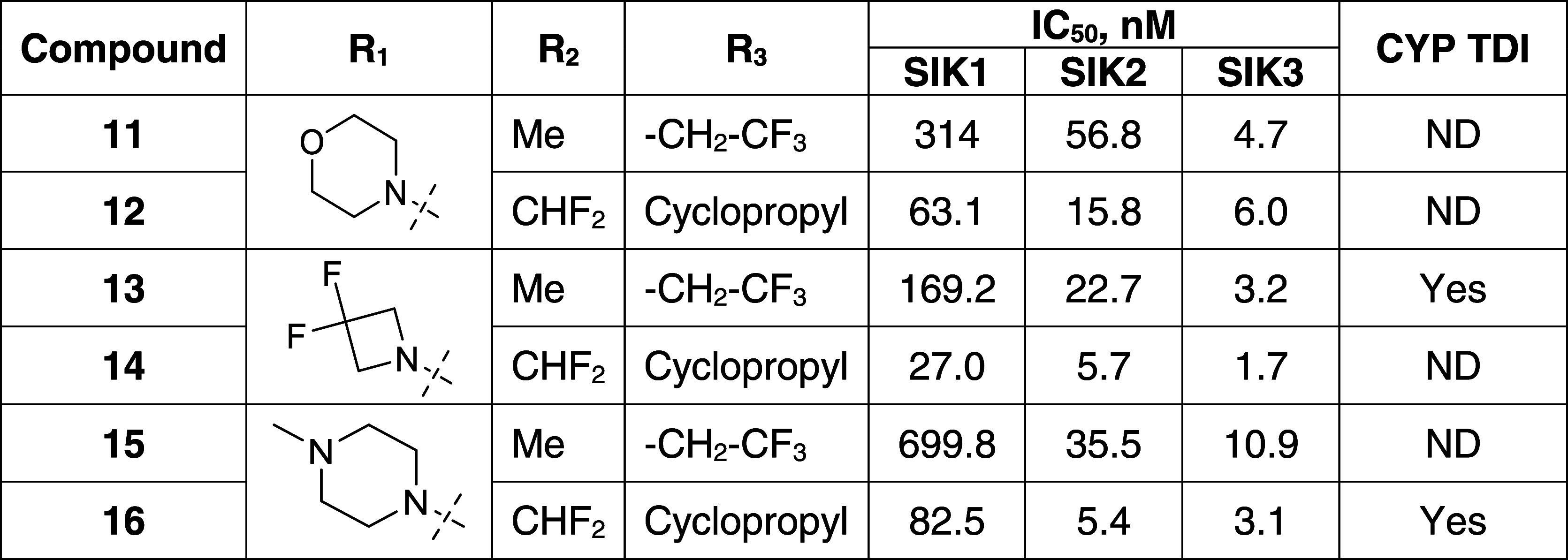
Matched Pairs SAR on Bottom Part^a^

a Abbreviations: ND, not determined; TDI, time-dependent inhibition.

Despite potent activity on SIK2 and SIK3 and encouraging selectivity against SIK1, progression of *N*-methylpiperazine derivative **16** was stopped owing to IC_50_ shift in a CYP TDI assay in human liver microsomes. This property is undesirable because it may lead to drug–drug interaction (DDI) or idiosyncratic drug toxicity.^21,22^

Further exploration of the amine substituent in position 7 of the imidazo[1,2-*a*]pyridine scaffold to increase selectivity against SIK1 was carried out with cyclopropylamide bottom part as summarized in Table 4.

**Table 4:**
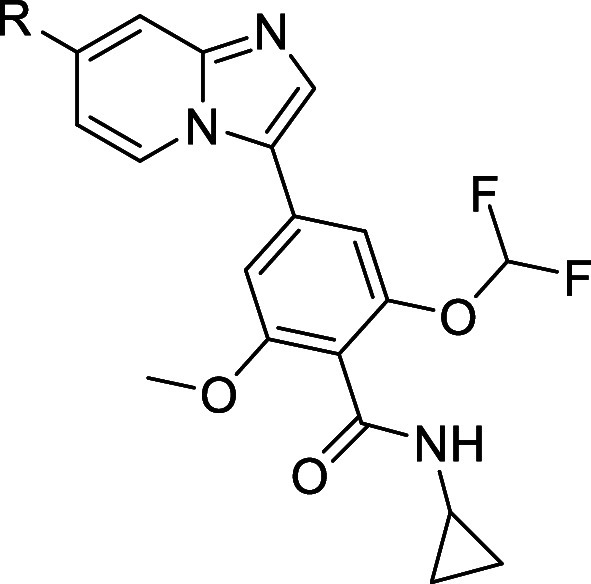

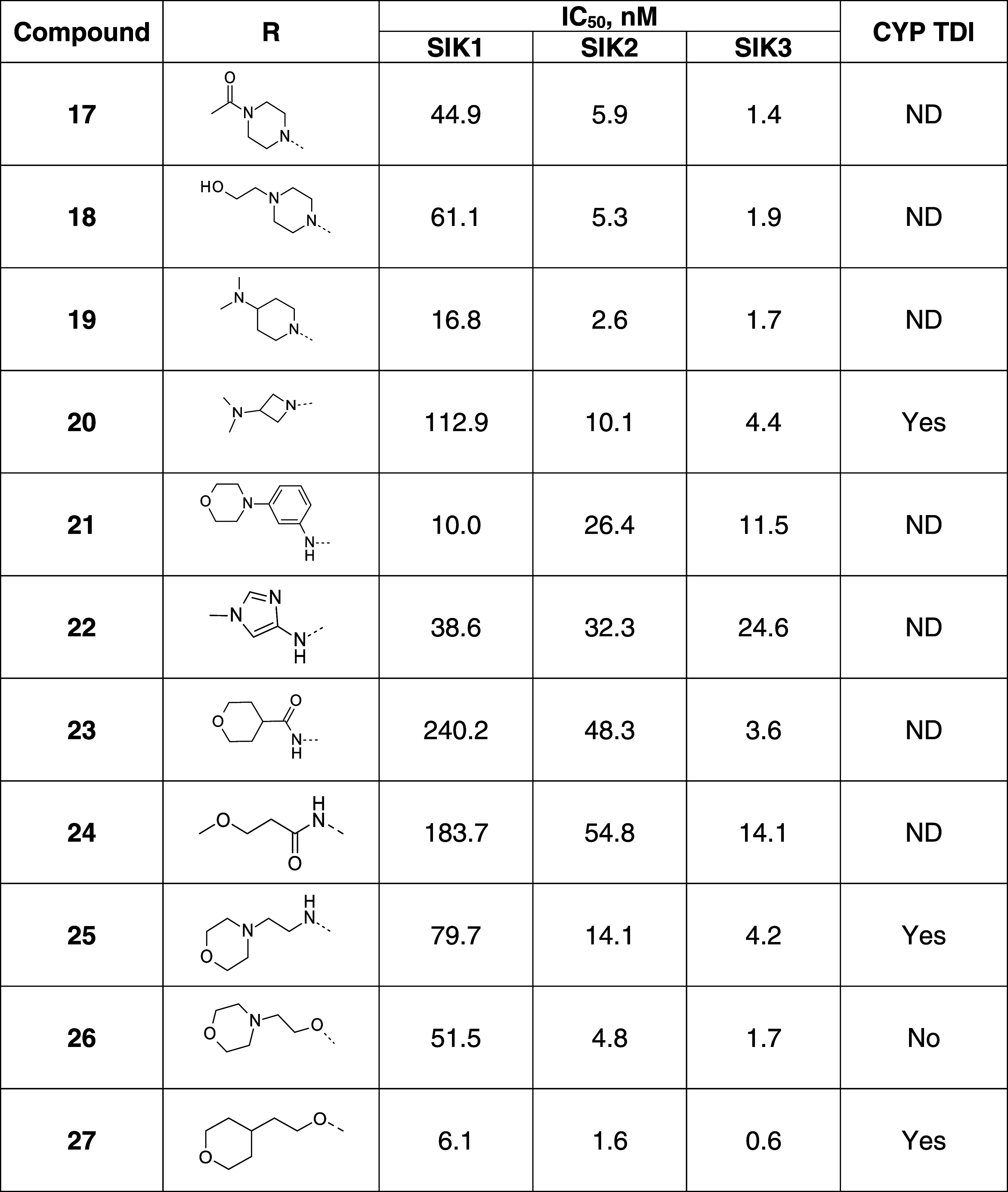
SAR Exploration in Position 7 of the Imidazo[1,2-*a*]pyridine Scaffold^a^

a Abbreviations: ND, not determined; TDI, time-dependent inhibition.

Replacement of the *N*-methyl basic center from **16** with a polar non-basic acetamide group in **17** retained comparable potency on SIKs (Table 4), as did extending the chain on the piperazine nitrogen with a hydroxyethyl group in **18**.

Introduction of a basic dimethyl amino group on position 4 of a piperidine ring substituent (**19**) maintained potency on SIK2 and SIK3, but it reduced the selectivity against SIK1. In contrast, analog **20** with a dimethyl amino group on an azetidine substituent retained comparable potency and selectivity as a methyl piperazine derivative **16**. These data suggested the position of the basic group was important for selectivity against SIK1. However, compound **20** turned out to be positive in the CYP TDI assay and so was not further progressed.

Substitution of the imidazo[1,2-*a*]pyridine scaffold by an aniline moiety in **21** or a heteroaromatic amine in **22** led to potent activity on SIKs with no isoform selectivity. Compounds **23** and **24** bearing an amide substituent could maintain potent activity on SIK3 with selectivity against SIK1 but resulted in a drop of activity on SIK2.

Incorporating a flexible acyclic linker between the imidazo[1,2-*a*]pyridine scaffold and the basic group was tolerated, as illustrated with compound **25** bearing a 2-morpholinoethylamine substituent, which displayed low nanomolar potency on SIK2 and SIK3, and some selectivity against SIK1. Replacing the nitrogen in the linker by oxygen in the ether analog **26** led to single-digit nanomolar activity on SIK2 and SIK3, and 10-fold/30-fold selectivity against SIK1, respectively. Similarly, replacing the morpholine moiety by a tetrahydropyran in derivative **27** led to a drop of selectivity against SIK1 to 4-fold/10-fold.

An important observation was made upon assessment in the CYP TDI assay; although the N-linked derivative **25** was positive in the CYP TDI assay, the O-linked analog **26** was negative, and the tetrahydropyran analog **27** was positive. Although the mechanism that led to CYP TDI was not investigated, we speculated that the imidazo[1,2-*a*]pyridine scaffold could be involved, as it is known to be a structural alert for risk of formation of reactive metabolites and occurrence of CYP TDI.^23−25^ Strategies to remove CYP TDI within a chemical series include the modification of physicochemical properties, the modulation of stereoelectronic properties of substituents, and the introduction of soft spots to redirect metabolism to another site.^26^ We hypothesized that the absence of CYP TDI for O-linked compound **26** resulted from a decreased electron density in the imidazo[1,2-*a*]pyridine ring system compared with the N-linked substituent in compound **25**. Data also suggest that the increase of lipophilicity in compound **27** (CLogP = 4.4) compared with **26** (CLogP = 3.8) restored CYP TDI activity. Since the ether linker could improve DDI properties, it was kept for further investigations, and lipophilicity of compounds was monitored.

Although residues are well conserved between SIK isoforms in the active site around the dimethoxybenzamide moiety, the possibility that more distal, non-conserved residues could lead to subtle differences in the conformation of the active site between isoforms and contribute to isoform selectivity was not excluded. Therefore, we explored the impact of modifying the benzamide part of ether derivative **26** on potency on SIKs and selectivity against SIK1 (Table 5). Replacing the phenyl ring with a pyridine ring which could form an intramolecular hydrogen bond was found to be detrimental to potency. Compounds **28** and **29** with a cyclopropyl amide and compound **30** with a trifluoroethyl amide displayed IC_50_ higher than 50 nM on the three SIK isoforms with some selectivity against SIK1 (Table 5). Cyclization of the amide with one of the meta positions to form a lactam ring substituted by a cyclopropyl group in derivative **31** led to an improvement of potency and selectivity compared with pyridine analog **28**. Finally, introduction of a trifluoroethyl group instead of a cyclopropyl group on the lactam ring gave GLPG3970 (**32**) which displayed single digit nanomolar potency on SIK2 and SIK3 with IC_50_ of 7.8 and 3.8 nM, and 36-fold/74-fold selectivity against SIK1, respectively (IC_50_ of 282.8 nM). Compound **32** displayed comparable lipophilicity to compound **26** and was negative in the CYP TDI assay.

**Table 5:**
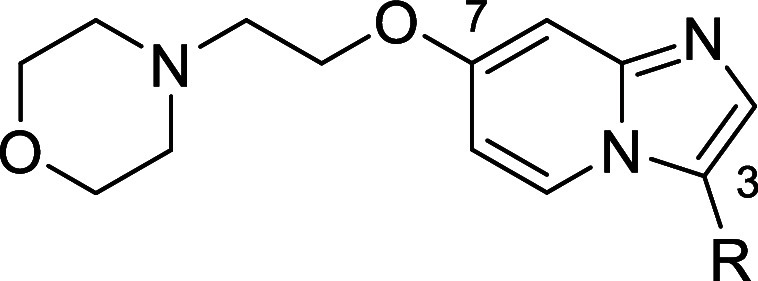

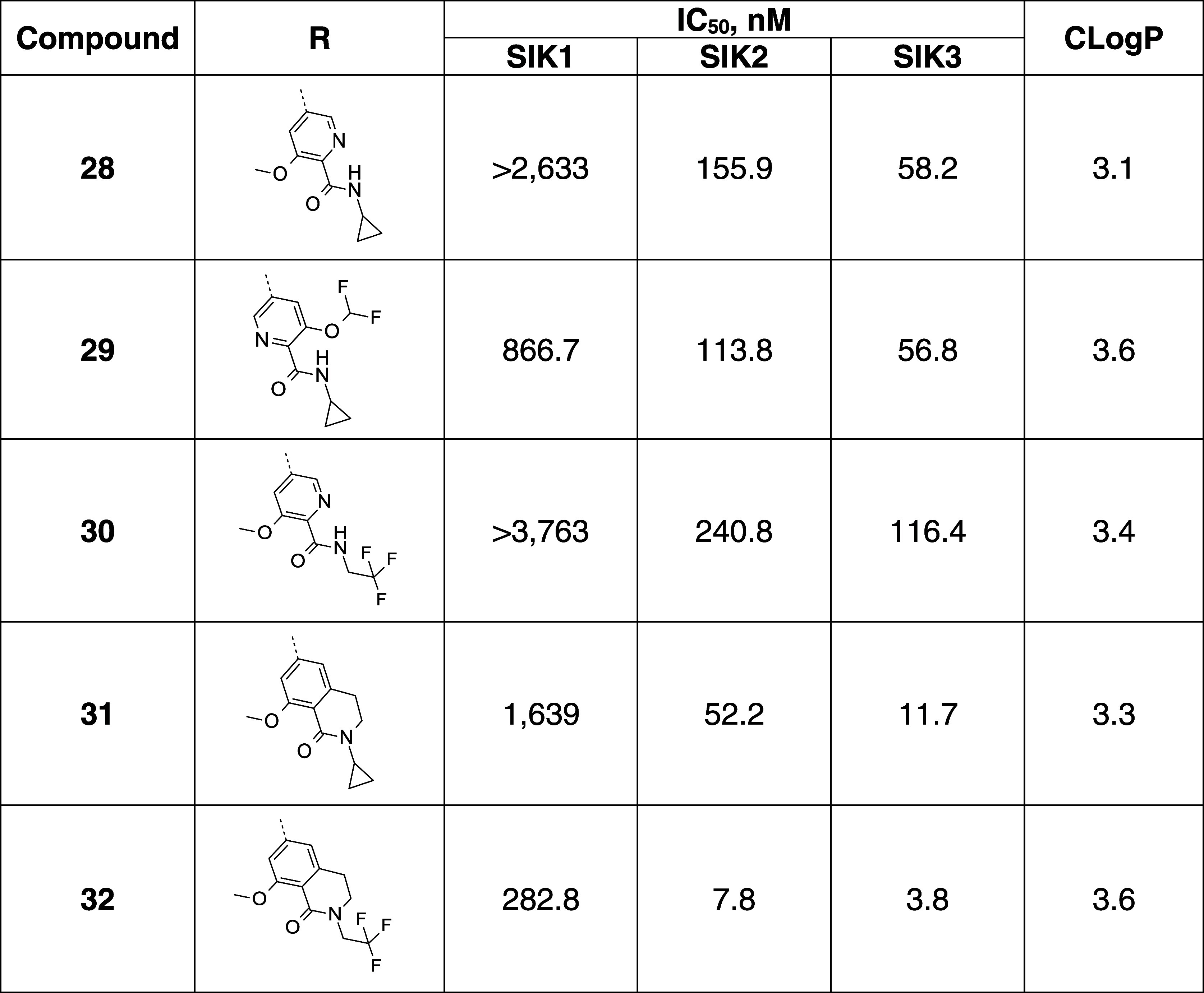
Bottom Part Exploration with Ether Substitution in Position 7 of the Imidazo[1,2-*a*]pyridine Scaffold

In summary, consideration of the cocrystal structure of compound **8** in SIK3 and the non-conserved residues in the different SIK isoforms around the pyrazole group bound to the benzimidazole scaffold prompted us to investigate structural modifications in this area to achieve selectivity against SIK1. Exploration started from hit compound **10** bearing a morpholine substituent on the benzimidazole scaffold instead of a pyrazole group, which displayed moderate activity on SIKs. Scaffold hopping from benzimidazole to imidazo[1,2-*a*]pyridine and changing the benzamide moiety, as in our previous investigation,^14^ boosted potency on SIKs. Replacing the morpholine substituent by an N-linked cyclic or acyclic substituent bearing a basic group could display potent activity on SIK2 and SIK3, and improve selectivity against SIK1. Oxygen-linked substitution as in analog **26** improved CYP TDI properties and displayed potent activity on SIK2 and SIK3, but better selectivity against SIK1 was desired. Finally, cyclization of the benzamide moiety into a lactam alkylated by a trifluoroethyl group led to ether analog **32** (GLPG3970), which displayed the desired levels of potency on SIKs and of selectivity against SIK1, and was negative in the CYP TDI assay.

### Molecular Modeling

**Figure 5:**
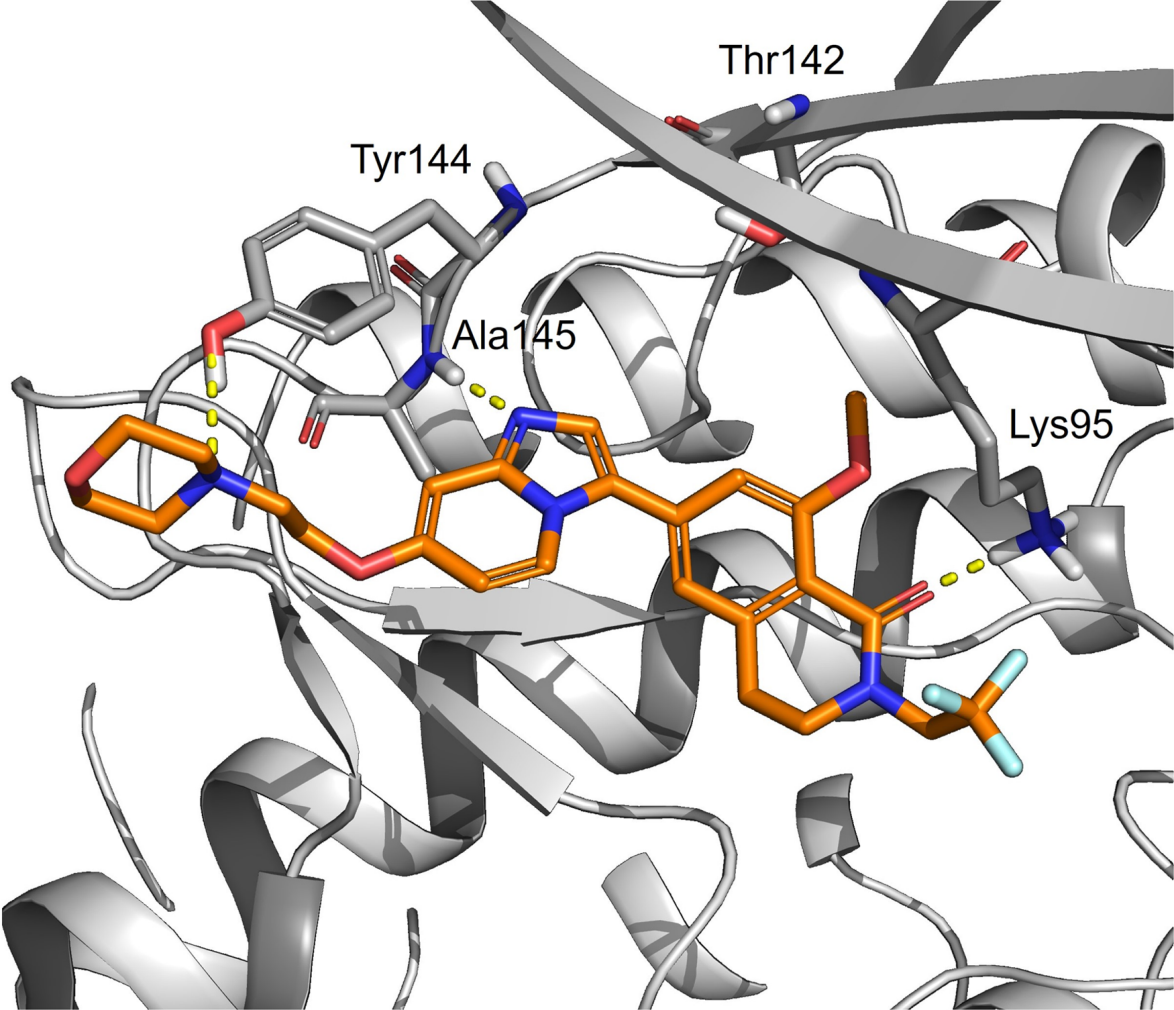
Representative frame from molecular dynamics simulations of compound **32** (orange sticks) in SIK3 (shown in white ribbons and gray carbons). The morpholine ring is facing Tyr144, present in SIK3 and SIK2. H-bond interactions are highlighted in yellow.

In order to understand the selectivity of compound **32**, docking and molecular dynamics simulations were carried out in SIK3. Compound **32** binds in the ATP site with the protein, adopting an active-like conformation as observed for compound **8**, and as such can be classed as a type 1 kinase inhibitor. In the simulations, the morpholine ring of **32** was occupying the solvent-exposed area while being able to maintain an H-bond interaction with Tyr144 (Figure 5). As shown in Figure 4, this tyrosine residue is conserved in SIK2 but not in SIK1, suggesting that interacting with this residue could favor a dual SIK2/SIK3 selectivity profile. In the molecular dynamics simulations of compound **32**, the carbonyl of the lactam was able to establish an H-bond interaction with the catalytic Lys95 (Figure 5). Considering that all the residues are conserved in the region where the lactam ring is hypothesized to be, molecular modeling investigations using homology modeling, docking, and molecular dynamics simulation did not provide an unambiguous rationale as to why the lactam ring is better tolerated in SIK2 and SIK3 than in SIK1.

### Chemistry

**Scheme 1:**
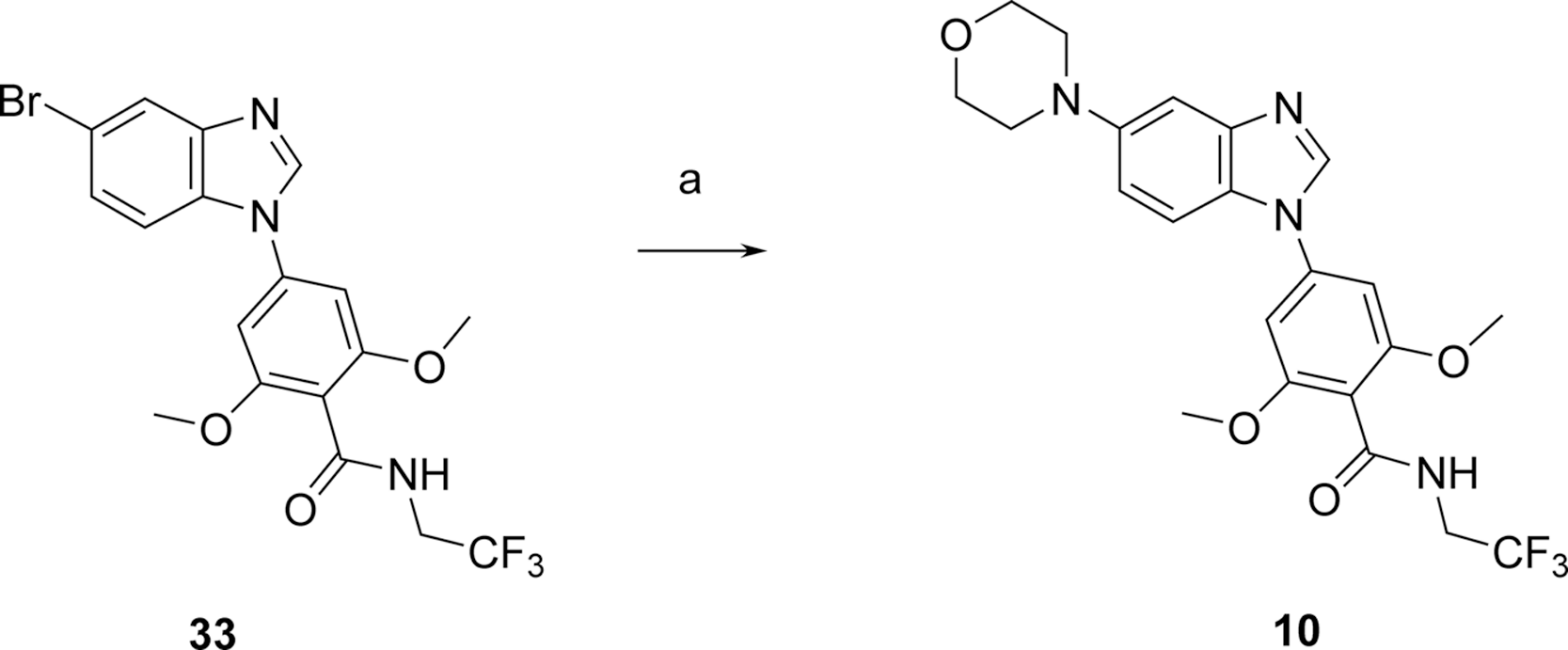
Preparation of Compound **10** Reagents and conditions: (a) morpholine, Pd_2_dba_3_, tBuXPhos, tBuOK, dioxane, 110 °C, overnight, 17%.

Benzimidazole derivative **10** was prepared from intermediate **33** described previously^14^ via a palladium-catalyzed Buchwald–Hartwig cross-coupling reaction with morpholine (Scheme 1). The preparation of imidazo[1,2-*a*]pyridine analogs **11**–**32** used 7-substituted or 3,7-disubstituted imidazo[1,2-*a*]pyridine building blocks and palladium-catalyzed cross-coupling, C–H insertion, amide coupling, or aromatic nucleophilic substitution reactions to introduce the desired substituents on positions 3 and 7 of the scaffold.

**Scheme 2:**
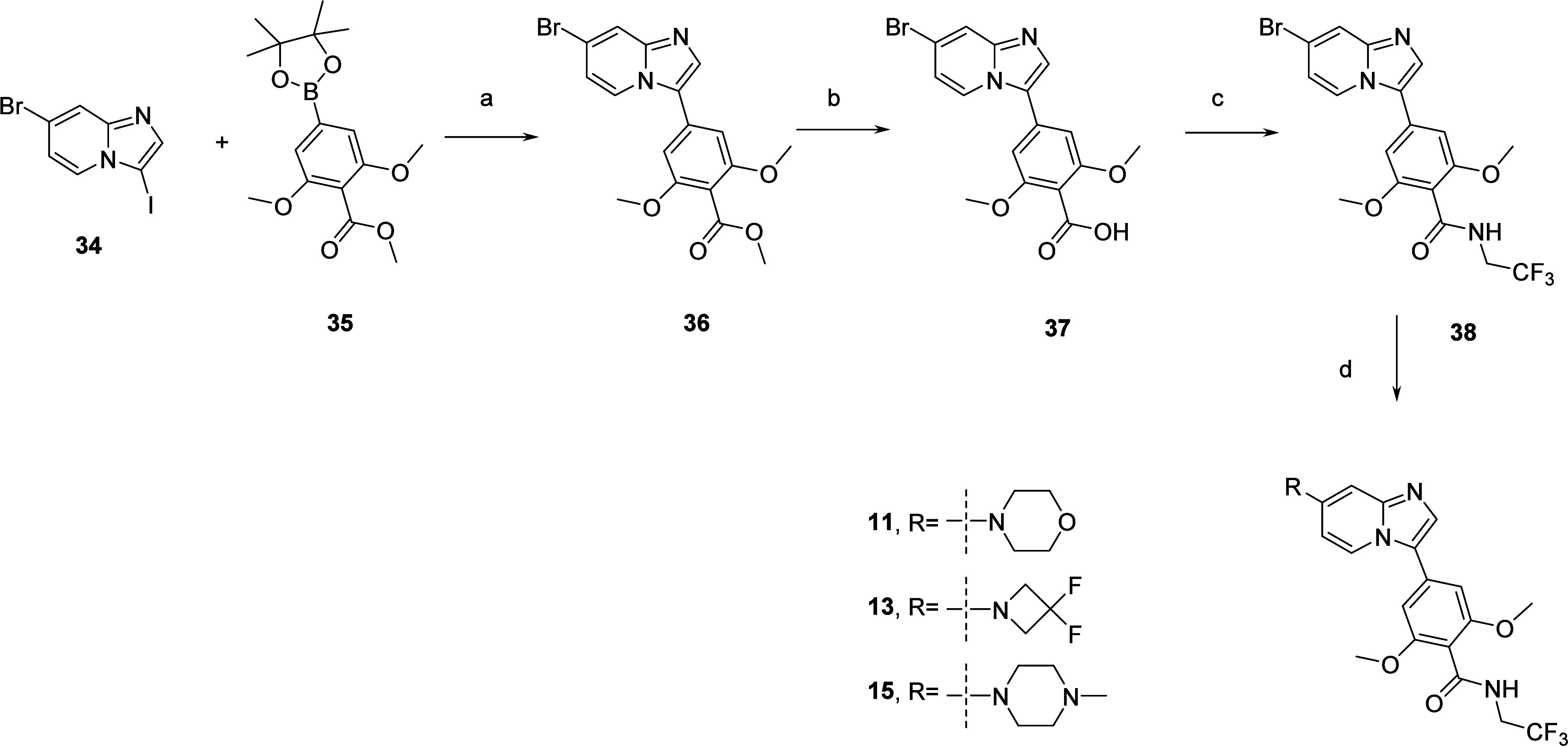
Preparation of Compounds **11**, **13**, and **15** Reagents and conditions: (a) Pd(dppf)Cl_2_, Cs_2_CO_3_, dioxane, water 90 °C, 1 h, 68%; (b) NaOH 2N, THF, MeOH, 70 °C, 18 h, 96%; (c) HATU, DIPEA, 2,2,2-trifluoroethylamine, DMF, rt, 18 h, 57%; (d) amine, RuPhos Pd G3, RuPhos, Cs_2_CO_3_, dioxane, 90 °C, 5 h to overnight, 36–83%.

The preparation of imidazo[1,2-*a*]pyridine analogs with 2,6-dimethoxy-*N*-(2,2,2-trifluoroethyl)benzamide moiety started with a Suzuki cross-coupling reaction between 7-bromo-3-iodo-imidazo[1,2-*a*]pyridine **34** and commercially available boronate **35** to obtain ester **36** (Scheme 2). Saponification followed by amide coupling with 2,2,2-trifluoroethylamine led to amide **38**. The Buchwald–Hartwig cross-coupling reaction with morpholine, 3,3-difluoroazetidine, and *N*-methylpiperazine afforded compounds **11**, **13**, and **15**, respectively.

**Scheme 3:**
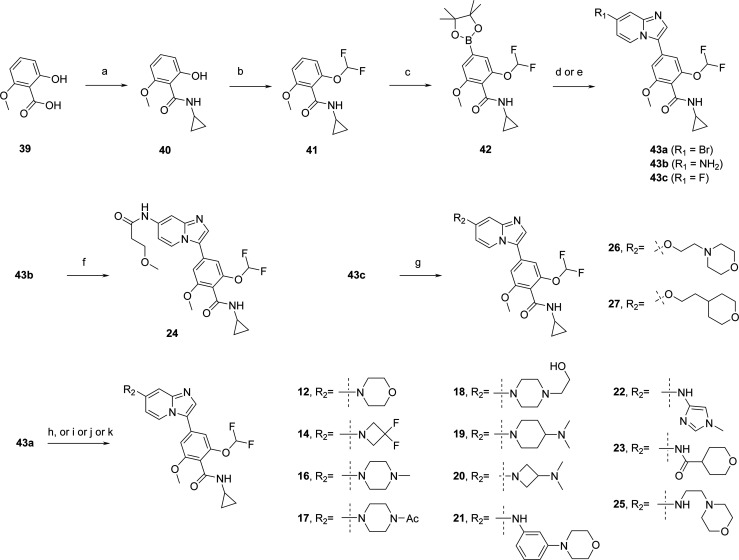
Preparation of Compounds **12**, **14**, **16**–**27** Reagents and conditions: (a) cyclopropylamine, HATU, DIPEA, DMF, rt, 3 d, 28%; (b) bromodifluoromethyl diethylphosphonate, KOH, ACN/water, −20 °C then rt, 1 h, 79%; (c) bis(pinacolato)diboron, [Ir(OCH_3_)(COD)]_2_, dtbpy, THF, 70 °C then rt, overnight, 49%; (d) 7-bromo-3-iodo-imidazo[1,2-*a*]pyridine (**34**), Pd(PPh_3_)_4_, Cs_2_CO_3_, dioxane, water, 90 °C, overnight, 96% (**43a**); (e) 3-bromoimidazo[1,2-*a*]pyridin-7-amine or 7-fluoro-3-iodoimidazo[1,2-*a*]pyridine, Pd(dppf)Cl_2_, Cs_2_CO_3_ or KOAc, dioxane/water or DMAC, 90 °C, 1 or 4 h, 65% (**43b**), 77% (**43c**); (f) 3-methoxypropionic acid, Ghosez’s reagent, DCM, rt, 4 d, 38%; (g) 2-morpholinoethanol or 2-tetrahydropyran-4-ylethanol, NaH, DMF, rt, overnight, 60–74%; (h) amine, RuPhos, RuPhos Pd G3, Cs_2_CO_3_, dioxane, 90 °C, overnight, 14–48%; (i) amine, RuPhos, RuPhos Pd G2, K_3_PO_4_, dioxane, 90 °C, 7 h, 19–24%; (j) 2-morpholinoethan-1-amine, 1-methyl-1*H*-imidazol-4-amine or 3-morpholinoaniline, Pd_2_(dba)_3_, XantPhos, Cs_2_CO_3_, dioxane, 90 °C, 2 h to overnight, 25–43%; (k) tetrahydropyran-4-carboxamide, Pd(OAc)_2_, Xantphos, dioxane, 150 °C (microwave irradiation), 1 h, 45% (**23**).

The exploration of analogs bearing a difluoromethoxy group on the phenyl ring required the preparation of the corresponding pinacol boronate (Scheme 3). Commercially available 2-hydroxy-6-methoxybenzoic acid **39** was transformed into the corresponding *N*-cyclopropyl-benzamide **40** via amide coupling. Difluoromethylation of the phenol group was performed using bromodifluoromethyl diethylphosphonate^27^ to generate the difluoromethoxy intermediate **41**. Direct and regioselective C–H borylation in the para position of the carboxamide moiety afforded the pinacol boronate building block **42**,^28^ which was engaged in Suzuki–Miyaura coupling with 3-halo-imidazo[1,2-*a*]pyridine scaffolds to obtain intermediates **43a**–**c**. Intermediate **43b** was reacted with 3-methoxypropionic acid activated with Ghosez’s reagent^29^ to give compound **24**. Nucleophilic aromatic substitution (SNAr) on fluoro derivative **43c** with the anion of 2-morpholinoethanol or 2-tetrahydropyran-4-ylethanol led to ether analogs **26** and **27**, respectively. Bromo intermediate **43a** was engaged in Buchwald–Hartwig cross-coupling reactions with amines, 3-morpholinoaniline, or 1-methyl-1*H*-imidazol-4-amine, to obtain compounds **12**, **14**, **16**–**23**, and **25**.

**Scheme 4:**
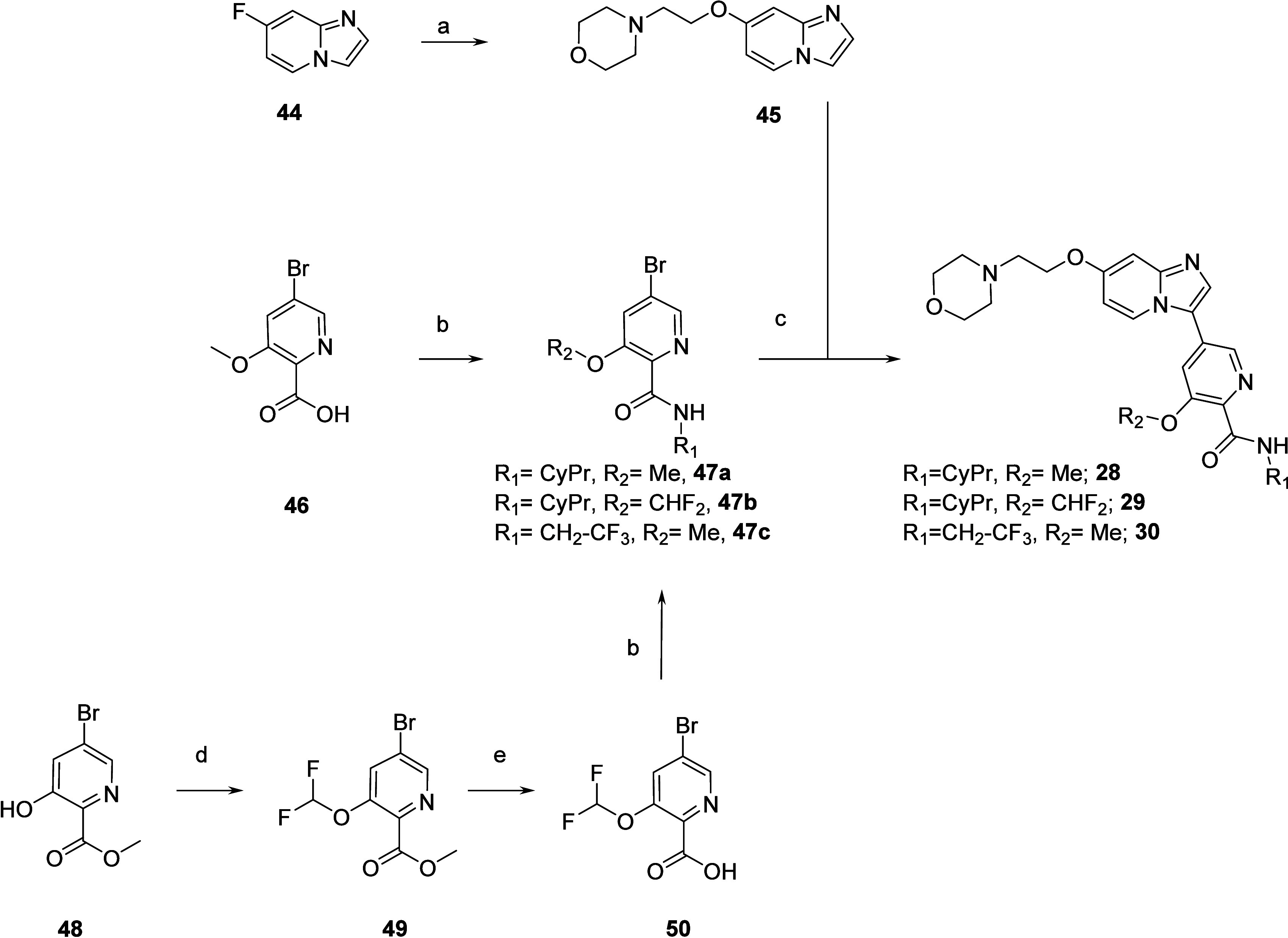
Preparation of Compounds **28**–**30** Reagents and conditions: (a) 2-morpholinoethanol, NaH, DMF, rt, 20 h, 100%; (b) amine, HATU, DIPEA, DMF, rt, 17–20 h, 54–83%; (c) KOAc, Pd(dppf)Cl_2_·DCM, DMAC, 110 °C, 2 h to overnight, 23–55%; (d) K_2_CO_3_, sodium chlorodifluoroacetate, acetonitrile, reflux, 2 h, 56%; (e) LiOH, THF, water, rt, 2 h, 85%.

In order to explore replacement of the benzamide part by heteroaryl groups, intermediate **45** was prepared by SNAr on 7-fluoro-imidazo[1,2-*a*]pyridine **44** with the anion of 2-morpholinoethanol (Scheme 4). 5-Bromo-picolinamide intermediates **47a**–**c** were prepared from picolinic acid reagents **46** and **50** via amide coupling with the corresponding amines. Picolinic acid reagent **50** was synthesized in 2 steps from commercially available methyl 5-bromo-3-hydroxypicolinate **48** by difluoromethylation (**49**) followed by saponification. Imidazo[1,2-*a*]pyridine intermediate **45** was reacted with 5-bromo-picolinamide intermediates **47a**–**c** using a palladium-catalyzed direct C–H arylation^30^ to deliver **28**–**30**.

**Scheme 5:**
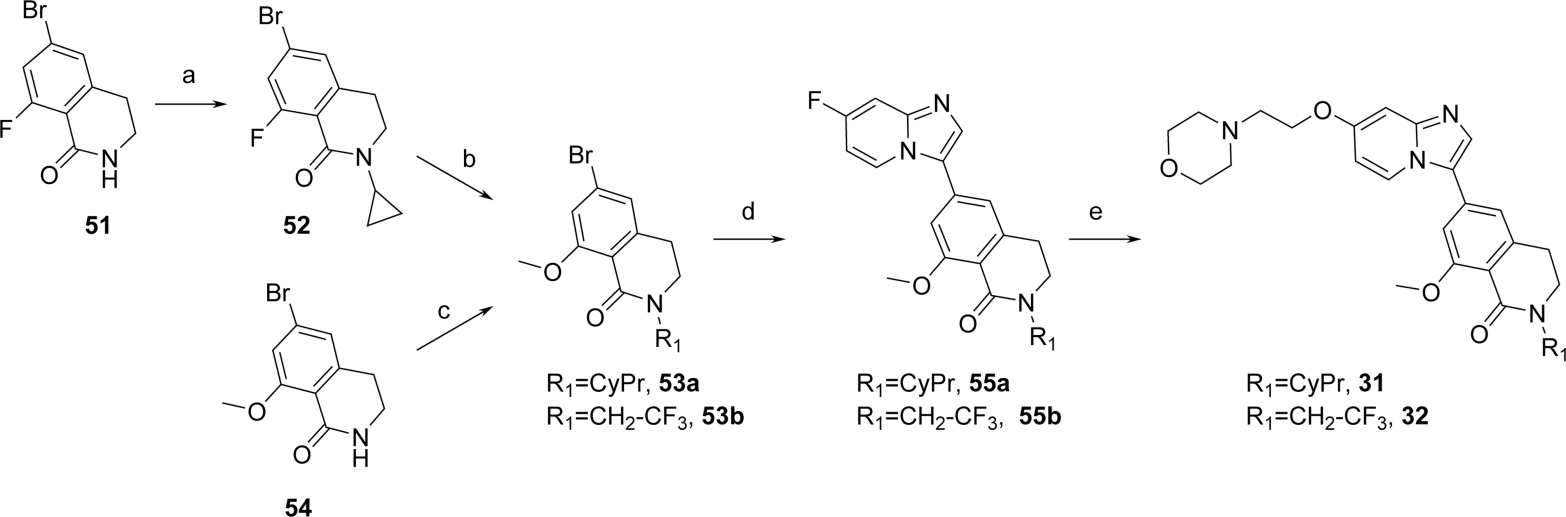
Preparation of Compounds **31** and **32** Reagents and conditions: (a) cyclopropylboronic acid, Cu(OAc)_2_, TEA, pyridine, THF, 70 °C, 18 h, 36%; (b) MeONa, MeOH, THF, rt, 1.5 h, 77%; (c) 2,2,2-trifluoroethyl trifluoromethanesulfonate, LiHMDS, THF, 0 °C to rt, 22 h, 76%; (d) 7-fluoroimidazo[1,2-*a*]pyridine (**44**), KOAc, Pd(dppf)Cl_2_·DCM, DMAC, 110 °C, 2 h, 77–93%; (e) 2-morpholinoethanol, NaH, DMF, rt, 5 h, 47–90%.

Introduction of 3,4-dihydroisoquinolin-1(2*H*)-one moiety started from commercially available building blocks **51** and **54** (Scheme 5). First, a Chan–Lam coupling^31^ on **51** was performed to install a cyclopropyl ring (**52**), then SNAr reaction with sodium methoxylate led to intermediate **53a**. The 2,2,2-trifluoroethyl chain of **53b** was introduced on commercially available **54** by N-alkylation of the lactam ring using 2,2,2-trifluoroethyl trifluoro-methanesulfonate. Palladium-catalyzed direct C–H arylation on 7-fluoroimidazo[1,2-*a*]pyridine with **53a**,**b** led to intermediates **55a**,**b** which underwent SNAr with the anion of 2-morpholinoethanol to give compounds **31** and **32**.

### Kinase Selectivity Profiling

**Figure 6:**
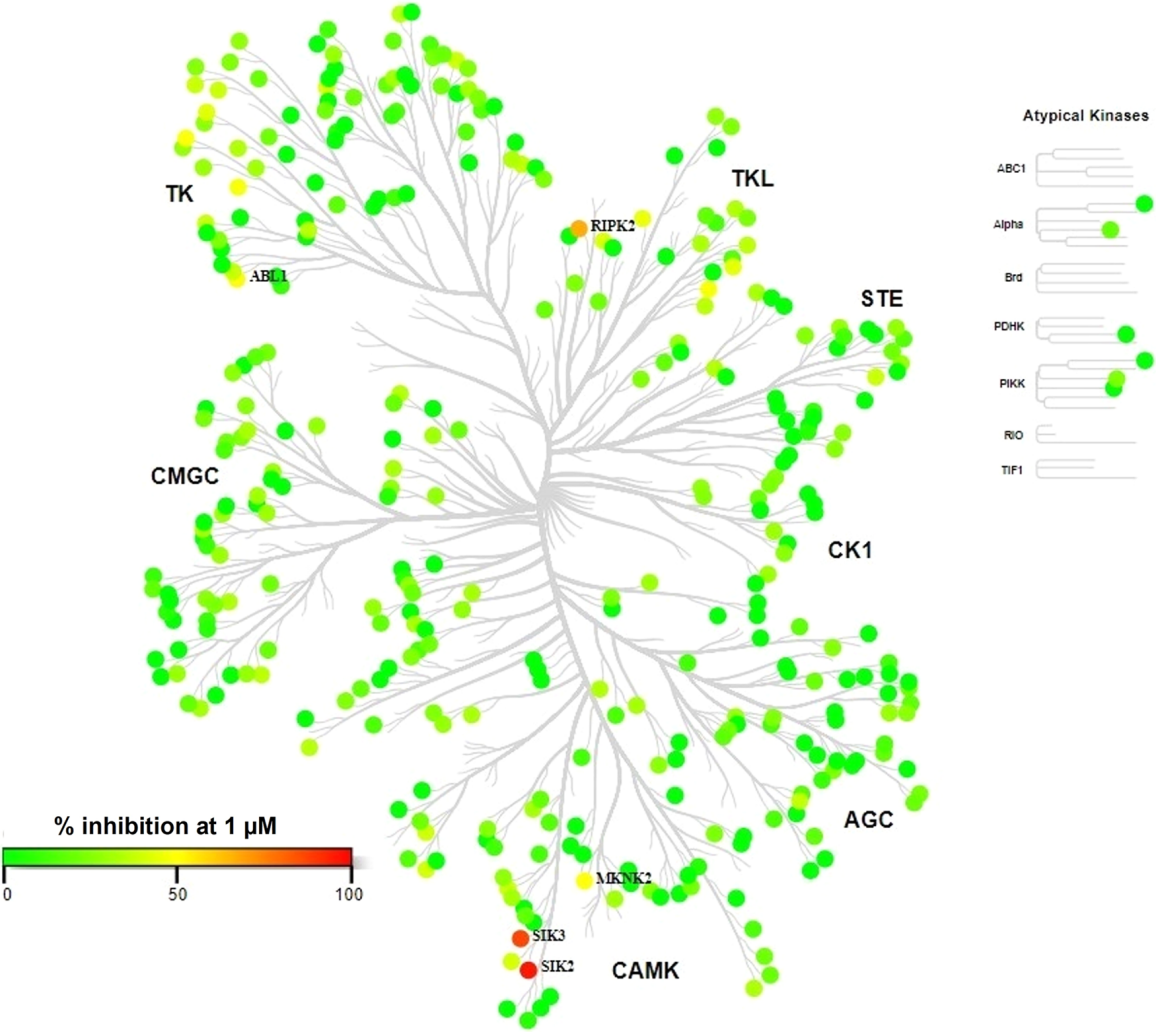
Kinome tree of **32** at 1 μM.

In addition to potent dual SIK2/SIK3 inhibition and selectivity against SIK1, we aimed to identify a compound with good kinome selectivity to explore the therapeutic potential of dual SIK2 and SIK3 inhibition only. The inhibition of enzymatic activity by compound **32** at a concentration of 1 μM was assessed against a panel of 372 kinases and is represented in Figure 6. Apart from SIKs, **32** had a percentage of inhibition (PIN) of kinase activity above 50% on only three other kinases: RIPK2 (79%), ABL1 (58%), and MKNK2 (54%). Further characterization led to IC_50_ values of 78.4 nM (RIPK2), 1,095 nM (ABL1), and 1,074 nM (MKNK2). **32** shows 10-fold/20-fold selectivity against RIPK2 based on potency in biochemical assays on SIK2 and SIK3, respectively. Since RIPK2 has been explored for the treatment of inflammatory diseases including IBD, RIPK2 inhibition was not considered as incompatible with further progression of **32**.^32,33^

In summary, profiling of compound **32** against a panel of 372 kinases at 1 μM showed excellent selectivity, with RIPK2 identified as the only off-target with an IC_50_ below 1 μM. Interestingly RIPK2 is also the main off-target for GLPG3312.^14^ Compound **32** is approximately 10-fold more potent on SIK2 and 20-fold more potent on SIK3 than on RIPK2. Compound **32** is, therefore, a highly selective dual SIK2/SIK3 inhibitor.

### *In Vitro* Pharmacology Profiling of **32**

Activity of **32** was explored in cellular assays to confirm the isoforms’ selectivity, monitor activity on target substrate, and assess phenotypic activity on immune cells.

SIK isoform selectivity of **32** in the cellular context was determined using NanoBRET binding assays in HEK293 cells and resulted in IC_50_ values of >17,500 nM, 254 nM, and 79 nM on SIK1, SIK2, and SIK3, respectively (Table 6). The NanoBRET kinase assays confirmed **32** to be a potent SIK2 and SIK3 binder highly selective against SIK1.

**Table 6:** Activity of **32** in Target-Based and Phenotypic Cellular Assays

**Target-Based Cellular Assays**		
Assay Model		IC_50_/EC_50_ (nM)
NanoBRET assay (HEK293 cells)	SIK1	>17,500
	SIK2	254
	SIK3	79
CRTC3 translocation assay (U2OS cells)		1,703


**Phenotypic Cellular and Whole Blood Assays**			
Assay Model	Trigger/Time	Cytokine Inhibition IC_50_ (nM)^a^	Maximum Fold Change Increase of IL-10^b^
Human monocytes	LPS/4 h	TNFα: 231 (5)	13.8 ± 1.9 (4)
Human monocytes	LPS/20 h	IL-12: 67 (5)	ND
Human monocyte-derived macrophages	LPS/20 h	TNFα: 365 (6)	ND
	LPS/2 h	ND	2.7 ± 0.4 (5)
Human whole blood	LPS/2 h	TNFα: 1,000 (52)	5.83 ± 0.32 (52)

a Number of replicates/donors for each readout indicated in parentheses.

b Compared with LPS stimulation with no compound treatment at the same time point. Abbreviations: IL-12, interleukin-12; ND, not determined; U2OS, human bone osteosarcoma cells.

**Figure 7:**
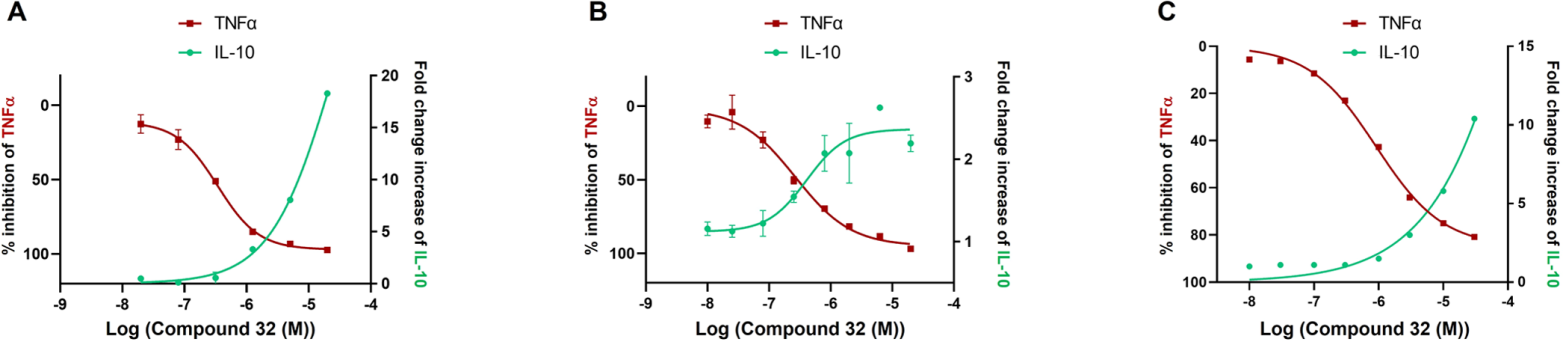
Representative graphs for inhibition of TNFα and induction of IL-10 by **32** in LPS-stimulated human monocytes (A), human monocyte-differentiated macrophages (B), and human whole blood (C). Data are represented as mean values of percentage of inhibition of TNFα and fold change increase of IL-10 levels. Abbreviations: IL, interleukin; LPS, lipopolysaccharide; TNFα, tumor necrosis factor alpha.

SIK inhibition has demonstrated the capacity to skew pro-inflammatory macrophages to an immunoregulatory phenotype with a mechanism that involves phosphorylation of CRTC3 by SIKs.^15^ As shown in Figure 1, inhibition of SIKs leads to dephosphorylation of CRTC3 and subsequent translocation to the nucleus. Compound **32** induced a concentration-dependent nuclear translocation of CRTC3 in U2OS cells with an EC_50_ of 1,703 nM, as measured by high-content imaging technology (Table 6). The anti-inflammatory and immunoregulatory activities of **32** were examined in phenotypic assays using human primary myeloid cell types and whole blood from healthy donors stimulated with LPS. In LPS-stimulated monocytes, **32** suppressed TNFα and IL-12 at IC_50_s of 231 and 67 nM, respectively. In monocyte-derived macrophages (MdMs), **32** inhibited TNFα at an IC_50_ of 365 nM. In both assays, **32** dose-dependently increased the production of IL-10 (Table 6, Figure 7). Data on IL-10 are expressed as fold-induction versus LPS trigger at the top concentration of 20 μM evaluated in the assay as the clear dose response obtained did not however allow accurate curve fitting on IL-10 induction for robust EC_50_ determination across different experiments. In human whole blood from healthy donors stimulated with LPS, compound **32** confirms its dual activity inhibiting TNFα production with an IC_50_ of 1,000 nM coupled with a dose-dependent IL-10 increase (Table 6, Figure 7).

Overall, compound **32** displayed high selectivity against SIK1 in cellular context. Nuclear translocation of CRTC3 was observed in the presence of **32**, indicating SIK1 is dispensable for this activity. Compound **32** inhibited the production of TNFα and IL-12, and increased the release of IL-10 by primary human myeloid cells and in human whole blood stimulated by LPS. Dual SIK2/SIK3 inhibition as with compound **32** therefore leads to both anti-inflammatory and immunoregulatory properties *in vitro*.

### Pharmacokinetic Properties of **32**

Pharmacokinetic properties of **32** were evaluated in mice, rats, and dogs. Mice models were used to assess the *in vivo* pharmacological activity of **32**. Rats and dogs are the preferred species for *in vivo* toxicology investigations in preclinical development, and pharmacokinetic properties from several preclinical species are generally used to make a prediction of human pharmacokinetic properties. Pharmacokinetic properties of **32** were evaluated in male mice, rats, and dogs with doses of 1 mg/kg and 5 mg/kg administered iv and po, respectively (Table 7). Following iv dosing in CD1 mice, **32** was characterized by moderate total and low unbound plasma clearances, a large steady-state volume of distribution, and a short apparent terminal half-life. Absolute oral bioavailability of 68.7% was observed in mice. In Sprague–Dawley rats, **32** was characterized by moderate total blood clearance, low unbound plasma clearance, large steady-state volume of distribution, and moderate apparent terminal half-life. Absolute oral bioavailability was 55.9% in rats. Following iv administration in beagle dogs, **32** was characterized by moderate total blood clearance, low unbound plasma clearance, large steady-state volume of distribution, and long apparent terminal half-life. Following po administration at 5 mg/kg in beagle dogs, absolute oral bioavailability was 41.0%.

**Table 7:** Pharmacokinetic Properties of **32**^a^

	Species		
	Mouse	Rat	Dog
Strain	CD1	Sprague–Dawley	Beagle
Doses (mg/kg)	1 iv/5 po	1 iv/5 po	1 iv/5 po
CL_b_ (L/h/kg)	2.48^b^	1.89 (21)	0.767 (26)
CL_u_ (L/h/kg)^c^	5.57	7.64	1.57
*V*_ss_ (L/kg)	2.51	2.82 (27)	2.89 (22)
Half-life, iv (h)	0.555	1.42 (8.1)	4.28 (11)
Half-life, po (h)	1.28 (34)	3.61 (31)	4.63 (26)
AUC_0–∞_, iv (ng·h/mL)	402	759 (21)	897 (7.0)
AUC_0–∞_, po (ng·h/mL)	1,380 (54)	2,120 (45)	1,840 (33)
*F* (%)	68.7	55.9	41.0

a Mean values; % coefficient of variation indicated in parentheses.

b Assuming blood-to-plasma ratio equals 1.

c Fraction unbound in plasma is 0.445, 0.246, and 0.507 in mouse, rat, and dog, respectively. Abbreviations: CL_b_, blood clearance; CL_u_, unbound plasma clearance; *V*_ss_, apparent volume of distribution at steady state.

Overall, **32** displayed moderate total clearance, low unbound plasma clearance, and moderate to high oral bioavailability across species. The pharmacokinetic properties of **32** made it suitable for further development.

### *In Vivo* Pharmacology Activity of **32**

**Figure 8:**
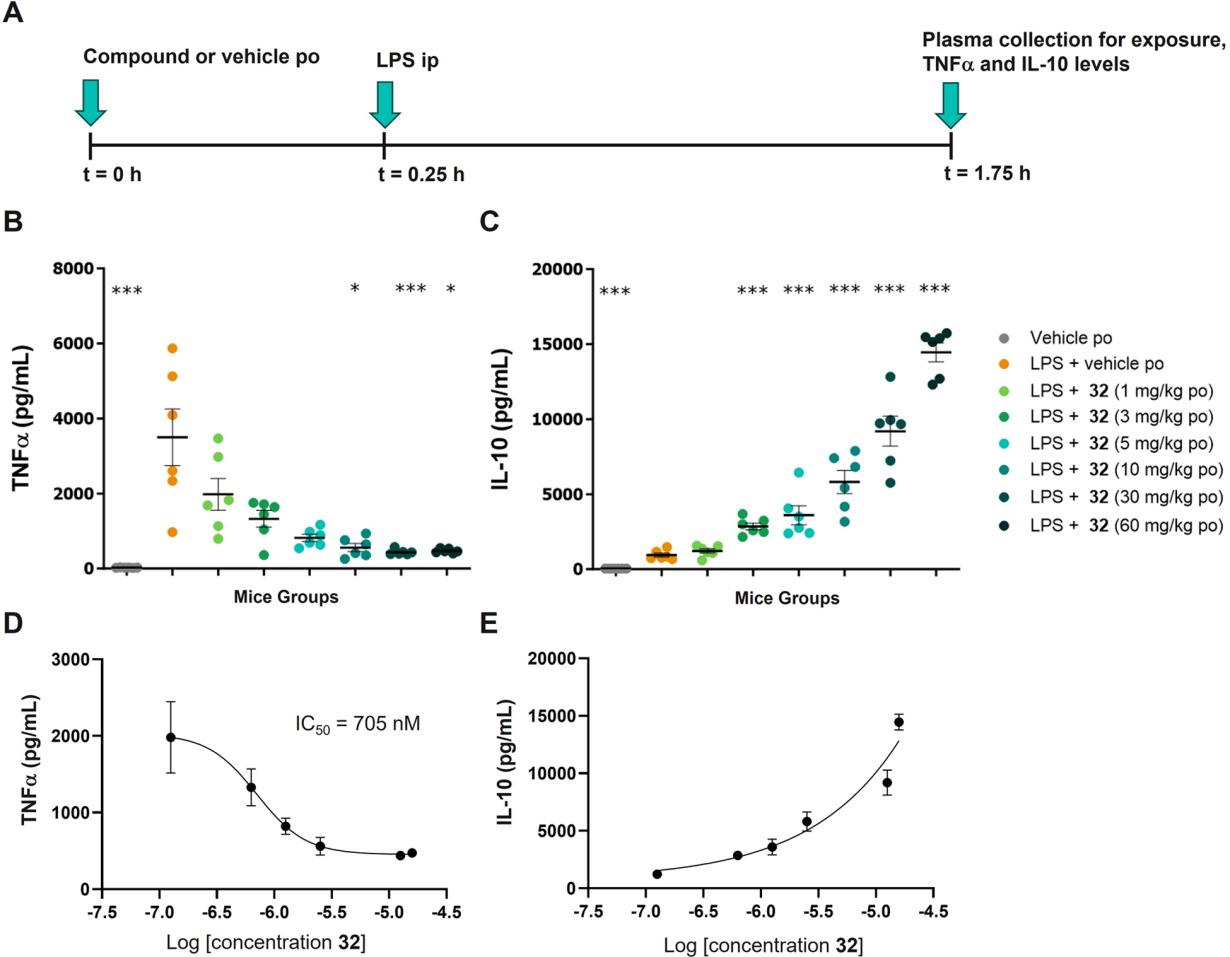
Plasma levels of TNFα and IL-10 after oral administration of **32** and *in vivo* LPS challenge in mice. (A) Study schematic illustrating administration of **32** (*n* = 6 mice/group) followed by administration of LPS before collecting plasma for exposure of **32** and cytokine levels. Data are presented as mean value levels of TNFα (B) and IL-10 (C) ± SEM for each group. Statistical analysis of plasma TNFα levels versus LPS + vehicle was performed with a Kruskal–Wallis and Dunn’s post-test: *p < 0.05; ***p < 0.001. Statistical significance of plasma IL-10 levels was calculated using ANOVA and Dunnett’s multiple comparison test: ***p < 0.001. Exposure–response curves for TNFα (D) and IL-10 (E) are shown using mean values for concentrations (ng/mL) and cytokine levels (±SEM). Abbreviations: ANOVA, analysis of variance; IL-10, interleukin-10.

For *in vivo* translation of the observed *in vitro* effect on TNFα and IL-10, the activity of **32** was explored in an acute LPS challenge model in mice. In this model, stimulation by LPS triggers an immune response with increased levels of TNFα and IL-10 circulating in blood. LPS was injected intraperitoneally 15 min after oral administration of **32** at doses of 1, 3, 5, 10, 30, and 60 mg/kg or corresponding vehicle (Figure 8A). Blood was collected 1.5 h post LPS stimulation, and levels of TNFα and IL-10 in plasma were quantified. As shown in Figure 8, compound **32** dose-dependently reduced the release of TNFα with significant inhibition higher than 85% at doses of 10, 30, and 60 mg/kg compared with vehicle group in mice stimulated with LPS, despite high inter-individual variability seen in the vehicle group (Table 8). Analysis of the exposure–response curve for **32** led to an IC_50_ of 705 nM on TNFα production. Compound **32** also dose-dependently increased IL-10 levels with a significant induction of 3.0-fold and above, starting at the dose of 3 mg/kg onward, relative to vehicle in mice stimulated with LPS. No EC_50_ was determined on IL-10 as the exposure–response curve did not reach a plateau.

**Table 8:** Plasma Exposure of **32** and Levels of TNFα and IL-10 in LPS Challenge in Mice^a^

compound **32** dose	*C*_plasma_ at 1.75 h (nM)	TNFα (pg/mL)	% inhibition TNFα vs LPS + vehicle	IL-10 (pg/mL)	fold-induction IL-10 vs LPS + vehicle
1 mg/kg po	129 ± 21	1,981 ± 426	44 ± 12	1,217 ± 149	1.3 ± 0.2
3 mg/kg po	705 ± 63	1,328 ± 220	63 ± 6	2,856 ± 226	3.0 ± 0.2
5 mg/kg po	1,216 ± 107	821 ± 96	77 ± 3	3,596 ± 628	3.8 ± 0.7
10 mg/kg po	2,729 ± 186	562 ± 107	85 ± 3	5,814 ± 767	6.2 ± 0.8
30 mg/kg po	13,871 ± 1,235	438 ± 30	88 ± 1	9,198 ± 997	9.8 ± 1.1
60 mg/kg po	17,578 ± 1,126	473 ± 23	87 ± 1	14,457 ± 626	15.3 ± 0.7

a Mean values with standard error of mean (SEM).

### Efficacy of **32** in the Murine DSS-Induced Colitis Model

**Figure 9:**
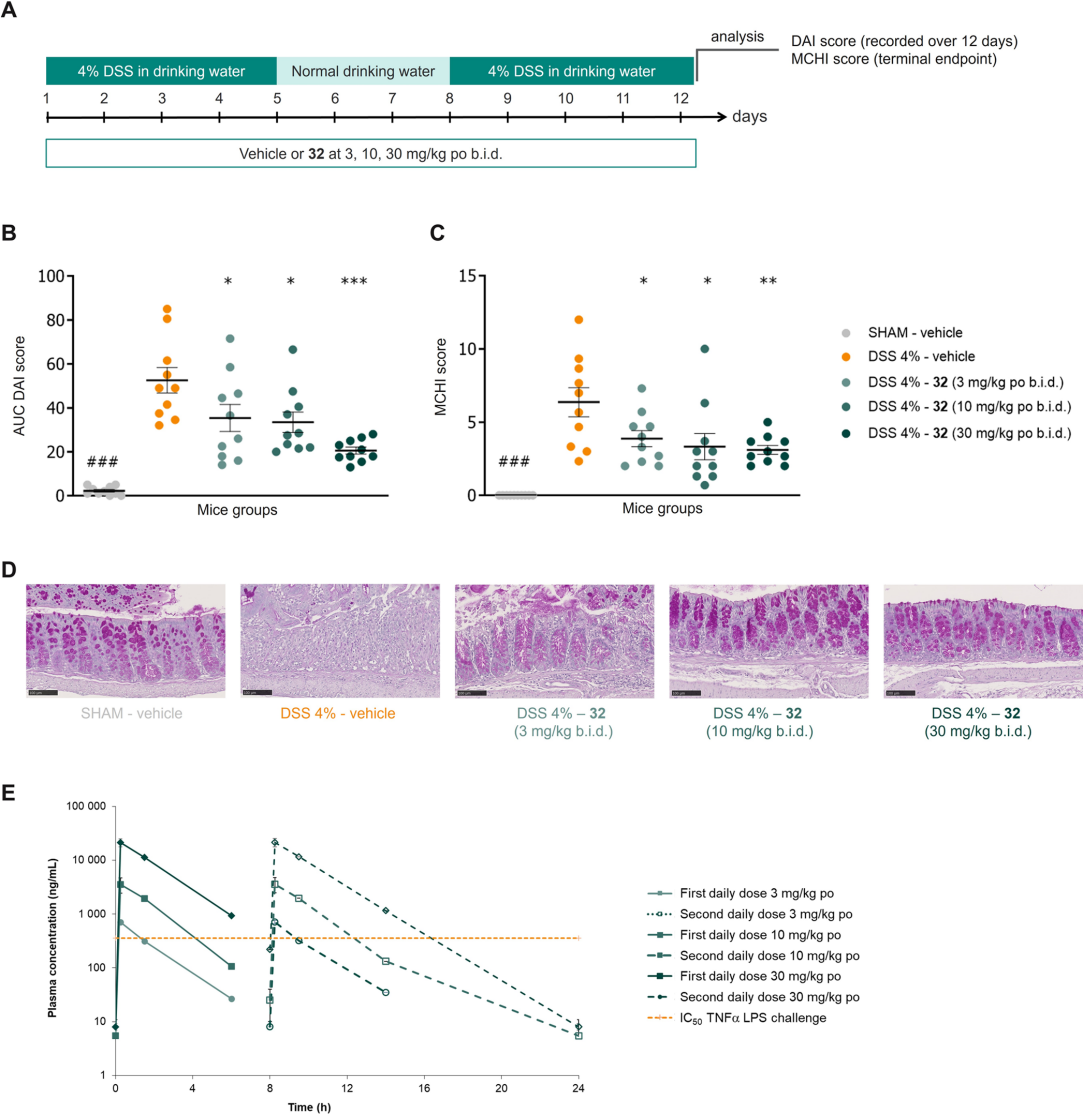
Activity of compound **32** in the DSS-induced colitis model. (A) Schematic setup of the mouse DSS-induced colitis model. (B) Activity of **32** on the AUC of DAI score (composite score of body weight loss, stool consistency, and fecal blood). Data are presented as mean values ± SEM (*n* = 10 mice/group). Statistical analysis of Log(Y) AUC DAI data transformation without SHAM – vehicle group was calculated using one-way ANOVA and Dunnett’s post-test analysis vs DSS 4% disease vehicle group: ^###^*p* < 0.001; *p < 0.05; **p < 0.01; ***p < 0.001. (C) Activity of **32** on MCHI score (mouse colitis histology index, a composite histological score of eight subscores following the published methodology).^35^ Data are presented as mean values ± SEM (*n* = 10 mice/group). Statistical analysis was performed with a one-way ANOVA without SHAM – vehicle group and Dunnett’s post hoc multiple comparison test vs DSS 4% disease vehicle group: ^###^*p* < 0.001; *p < 0.05; **p < 0.01; ***p < 0.001. (D) Representative images (scale 100 μm) of PAS-stained (and hematoxylin counter-stained) colonic tissues collected at study termination from each treatment group: “SHAM – vehicle”, “DSS 4% disease – vehicle”, and “DSS 4% disease – compound **32**” treated with 3, 10, and 30 mg/kg (b.i.d. po). (E) Plasma exposure of compound **32** in the mouse DSS-induced colitis model on day 9 (*n* = 2 or 3 mice/time point between 0 and 6 h). The curve of the second daily dose was extrapolated and indicated by a dashed line for each group. Abbreviations: ANOVA, analysis of variance; AUC, area under the curve; DSS, dextran sulfate sodium; DAI, disease activity index; MCHI, mouse colitis histology index; PAS, periodic acid–Schiff; SEM, standard error of the mean.

*In vivo* efficacy of compound **32** was tested in a mouse DSS-induced colitis model to evaluate its therapeutic potential for IBD.^34^ In this model, ulcerative colitis-like inflammation is induced by 2 cycles of 4 days 4% DSS in drinking water (Figure 9A). Disease evolution is recorded as disease activity index (DAI), a composite score of body weight (BW) loss, stool consistency, and fecal blood over a course of 12 days. Efficacy was further assessed by histological examination of colonic tissues collected at study termination using a mouse colitis histology index (MCHI) score. MCHI is a composite scoring system of eight histological components (inflammatory infiltrate, goblet cell loss, crypt hyperplasia, crypt density, muscle thickness, submucosal infiltration, ulcerations, and crypt abscesses), categorized from 0 to 3 and summed into a total MCHI score. The MCHIs were optimized as valid and reliable measures of intestinal inflammation in mice, responsive to treatment effects in preclinical studies, and relevant to clinical IBD where histopathologic indices are routinely used as outcome measures in controlled clinical trials.^35^ Activity of **32** was assessed by prophylactic treatment at doses of 3, 10, and 30 mg/kg (twice daily, po). As seen for vehicle groups, the DSS treatment significantly increased the area under the curve (AUC) of DAI score (sum of scores for BW loss, stool consistency, and fecal blood), reflecting active colitis. In this study, oral treatment with **32** at 3, 10, or 30 mg/kg twice daily reduced dose-dependently and significantly the AUC of DAI score by 29, 33, and 59%, respectively, compared with mice treated with vehicle only (Figure 9B). In addition to reduced DAI score, treatment with **32** dose-dependently and significantly reduced histological end points as recorded by the MCHI (Figure 9C). Representative images of periodic acid–Schiff (PAS) staining visualizing mucin (in pink) produced by goblet cells and hematoxylin counter-staining for mucosal leukocyte infiltration are shown in Figure 9D. Oral treatment with **32** at 30 and 10 mg/kg twice daily clearly demonstrated prevention of goblet cell loss, reduced inflammatory infiltrates, decreased crypt density, and reduced mucosal erosion.

Analysis of plasma exposure of **32** showed more than dose-proportional exposure increase in the study as highlighted by the AUC/dose ratio (Table 9). As shown in Figure 9E, the dose of 3 mg/kg twice daily led to only a short coverage of the IC_50_ on TNFα from *in vivo* LPS challenge. In contrast, exposure at 30 mg/kg twice daily was above the IC_50_ on TNFα for approximately 16 h. The significant activity at 3 mg/kg twice daily suggests that this colitis model is sensitive to local SIK inhibition in the gut such that local exposure in the gut contributes to efficacy. Overall compound **32** dose-dependently reduced disease activity in a mouse DSS-induced colitis model.

**Table 9:** Plasma Exposure of **32** in a DSS-Induced Colitis Model on Day 9

	Dosing Regimen		
	3 mg/kg po b.i.d.	10 mg/kg po b.i.d.	30 mg/kg po b.i.d.
*C*_max_ (ng/mL)	705	3,580	21,300
AUC_0–24_ (ng·h/mL)	2,490	13,900	86,800
AUC/dose	415	695	1,447

In summary, the dual SIK2/SIK3 inhibitor **32** decreased the production of TNFα and increased the release of IL-10 in mice stimulated with LPS. These results suggest SIK1 inhibition is dispensable to exert a dual mechanism of immunomodulatory activity by compound **32***in vivo* in a short LPS challenge model along with robust preclinical activity in a mouse model of DSS-induced colitis.

### Drug–Drug Interaction and *In Vitro* Safety Properties

Further *in vitro* properties of **32** were evaluated to assess the risk of DDI and potential safety concerns. Reversible inhibition of CYP isoforms by **32** was determined in human liver microsomes (HLMs) and led to IC_50_ > 33 μM for CYP2C19 and CYP2C9, and IC_50_ > 100 μM for CYP3A4, CYP2D6, and CYP1A2 (Table 10). As mentioned previously, **32** was negative in the CYP3A4 TDI assay in HLMs using midazolam and testosterone as probe substrates. The compound also did not show any significant induction of CYP3A4 mRNA at 10 μM in primary human hepatocytes. **32** inhibited hERG channel in a manual patch clamp assay with an IC_50_ of 15.3 μM. Finally, the compound was negative in *in vitro* assays for genotoxicity with and without S9 metabolic activation. Overall properties of **32** were considered suitable for further development.

**Table 10:** DDI and *In Vitro* Safety Properties of **32**^a^

CYP450: IC_50_ (μM) in HLMs	>33: 2C19, 2C9
	>100: 3A4, 2D6, 1A2
CYP3A4 HLM-TDI midazolam/testosterone: IC_50_ (μM) initial, change (fold shift)	>33, no shift/>100, no shift
CYP3A4 mRNA induction at 10 μM in hepatocytes: fold increase vs vehicle, % increase vs rifampicin	<2-fold
	<20%
hERG: IC_50_ (μM) (manual patch clamp assay)	15.3
Genotoxicity Ames/micronucleus +/– S9	Negative/negative

a Abbreviations: HLM, human liver microsome; TDI, time-dependent inhibition.

## Conclusion

Starting from the same hit series that led to the pan-SIK inhibitor GLPG3312 and taking advantage of the cocrystal structure obtained previously, structural modifications of the chemotype were made in areas where differences of residues were identified between SIK isoforms. SAR optimization of amine substituents was performed, aiming at increasing potency on SIKs and selectivity against SIK1. Introduction of an oxygen linker improved the CYP TDI properties. Unexpectedly, scaffold modification and cyclization of the benzamide moiety into a lactam ring positively impacted potency and selectivity and allowed identification of GLPG3970 (**32**), a potent dual SIK2/SIK3 inhibitor showing high selectivity against SIK1 and high kinome selectivity. *In vitro* analysis showed that **32** had an immunomodulatory effect by inhibiting the production of TNFα and stimulating the release of IL-10 from LPS-stimulated human monocytes and MdMs, with similar effects seen in human whole blood from healthy volunteers. Compound **32** showed concordant results on TNFα and IL-10 *in vivo* in mice challenged with LPS. **32** displayed significant efficacy in a mouse DSS colitis model, suggesting that SIK1 inhibition is dispensable for immunoregulatory properties and activity in IBD models. Finally, **32** showed suitable pharmacokinetics, ADMET, and *in vitro* safety properties for further development. The results of clinical investigations with **32** will be described in a dedicated report.

## Experimental Section

All reagents were of commercial grade and were used as received, without further purification. Full-length SIK3 protein and compounds **8** and **9** were prepared as described previously.^14^ Testing of **32** on 372 kinases panel and follow-up IC_50_ determination were performed at Eurofins (Eurofins Cerep, Le Bois l’Evêque, France). *Homo sapiens* SIK1 (full length, reference 02-131), was purchased from Carna Biosciences. *Homo sapiens* SIK2 (full length, reference PR8353A), was purchased from Invitrogen. AMARA peptide (AMARAASAAALARRR, A11-58) was obtained from SignalChem. NanoBRET Kinase Tracer-04 (CS181041), Extracellullar NanoLuc inhibitor (CS181048), NanoBRET Nano-Glo substrate (CS181063A or CS181046) were purchased from Promega. Alexa Fluor 594-labeled goat anti-rabbit secondary antibody (A-11012) was obtained from ThermoFisher Scientific. Rabbit monoclonal antibody to human CRTC3 (AB91654) was purchased from Abcam. JetPEI (101-40) was obtained from PolyPlus. HLM were obtained from BD Gentest. GraphPad Prism software was used for generation and fitting of graphs, IC_50_ determination, and statistical analysis. Pharmacokinetic parameters were calculated using Phoenix software (Certara, version 6.4.0.768). Commercially available anhydrous solvents were used for reactions conducted under an inert atmosphere. Reagent-grade solvents were used in all other cases, unless otherwise specified. Column chromatography was performed on silica gel 60 (thickness: 35–70 μm). ^1^H NMR spectra were recorded on a 400 MHz Bruker Avance spectrometer (SEI probe) or a 300 MHz DPX Bruker spectrometer (QNP probe). Chemical shifts (δ) for 1H NMR spectra are reported in ppm relative to tetramethylsilane (δ 0.00) or the appropriate residual solvent peak (i.e., CHCl_3_ [δ 7.27], as internal reference). Multiplicities are given as singlet (s), doublet (d), doublet of doublets (dd), doublet of doublet of doublets (ddd), doublet of quartets (dq), doublet of triplets (dt), doublet of triplet of doublets (dtd), triplet (t), quartet (q), quintuplet (quin), multiplet (m), and broad (br). Electrospray MS spectra were obtained with a Waters Acquity UPLC instrument equipped with a Waters Acquity photodiode array detector and single quad detector mass spectrometer. Columns used were UPLC bridged-ethylene hybrid (BEH) C18 1.7 μm, 2.1 × 5 mm VanGuard precolumn with Acquity UPLC BEH C18 1.7 μm, 2.1 × 30 mm column, or Acquity UPLC BEH C18 1.7 μm, 2.1 × 50 mm column. All of the methods used MeCN/H_2_O gradients. MeCN and H_2_O contained either 0.1% formic acid or 0.05% NH_3_. As needed an autopurification system from Waters was used for LC-MS purification. LC-MS columns used were Waters XBridge Prep C18 5 μm, ODB 30 mm inner diameter (ID) × 100 mm length (L) (preparative column); and Waters XBridge C18 5 μm, 4.6 mm ID × 100 mm L (analytical column). All of the methods used MeCN/H_2_O gradients. MeCN and H_2_O contained either 0.1% formic acid or 0.1% diethylamine. All final compounds reported were analyzed using these analytical methods, and purities were greater than 95% unless otherwise indicated.

### Chemistry

#### General Procedure A for Buchwald–Hartwig Cross-Coupling Reaction

In a microwave vial under inert atmosphere, to a solution of 7-bromoimidazo[1,2-*a*]pyridine derivative (1.0 equiv) in dioxane (10 mL per mmol) were added amine (1.5–2 equiv), Cs_2_CO_3_ (2–3 equiv), RuPhos Pd G3 (0.1–0.2 equiv), and RuPhos (0.1–0.2 equiv). The reaction mixture was degassed and stirred at 90 °C until completion of the reaction (monitored by HPLC). The mixture was quenched with saturated aqueous NaHCO_3_ and extracted with AcOEt. The combined organic layers were dried over Na_2_SO_4_, filtered, and evaporated under reduced pressure. The residue was purified by preparative LC-MS to afford the desired compound.

#### 2,6-Dimethoxy-4-(5-morpholinobenzimidazol-1-yl)-*N*-(2,2,2-trifluoroethyl) benzamide, (**10**)

In a sealed tube containing a previously degassed solution of **33** (50 mg, 0.1 mmol, 1 equiv) and morpholine (13 μL, 0.15 mmol, 1.5 equiv) in dioxane (0.7 mL) under nitrogen atmosphere were added potassium *tert*-butoxide (34 mg, 0.3 mmol, 3 equiv), tBuXPhos (8.5 mg, 0.02 mmol, 0.2 equiv), and catalyst Pd_2_dba_3_ (9.1 mg, 0.01 mmol, 0.1 equiv). The vial was sealed, and the resulting solution was heated to 110 °C overnight. The reaction mixture was then diluted with DCM and water. The organic layer was separated, washed with water and brine, dried over MgSO_4_, filtered, and concentrated *in vacuo*. The crude residue was purified by flash chromatography on silica gel (gradient DCM/MeOH 100/0 to 90/10) to deliver **10** (8 mg, 17%). ^1^H NMR (400 MHz, CDCl_3_) δ ppm ^1^H NMR (400 MHz, DMSO-*d*_6_) δ 8.85 (t, *J* = 6.4 Hz, 1H), 8.53 (s, 1H), 7.62 (d, *J* = 8.9 Hz, 1H), 7.26 (d, *J* = 2.3 Hz, 1H), 7.12 (dd, *J* = 9.0, 2.3 Hz, 1H), 6.97 (s, 2H), 4.01 (qd, *J* = 9.7, 6.3 Hz, 2H), 3.83 (s, 6H), 3.81–3.73 (m, 4H), 3.16–3.09 (m, 4H). LC-MS: *m*/*z* = 465.2 [M+H].

#### Methyl 4-(7-bromoimidazo[1,2-*a*]pyridin-3-yl)-2,6-dimethoxy-benzoate (**36**)

To 7-bromo-3-iodo-imidazo[1,2-*a*] pyridine **34** (6.97 g, 21.58 mmol, 1 equiv), methyl 2,6-dimethoxy-4-(4,4,5,5-tetramethyl-1,3,2-dioxaborolan-2-yl)benzoate **35** (8.6 g, 25.90 mmol, 1.2 equiv), Cs_2_CO_3_ (16 g, 43.16 mmol, 2 equiv), and dioxane/water solvent mixture (4/1, 210 mL) degassed with N_2_, Pd(dppf)Cl_2_ (1.77 g, 2.16 mmol, 0.1 equiv) was added, and the mixture was purged with N_2_ then stirred at 90 °C for 1 h. Dioxane was evaporated, saturated aqueous NaHCO_3_ was added, and the mixture was extracted with EtOAc. The combined organic layers were washed with water and brine, dried over anhydrous MgSO_4_, filtered, and concentrated *in vacuo*. The crude was purified by flash chromatography on silica gel (eluting with a gradient of 10% to 30% EtOAc in CH_2_Cl_2_) to afford **36** (5.78 g, 68%). ^1^H NMR (400 MHz, DMSO-*d*_6_) δ ppm 8.66 (dd, *J* = 7.4, 0.8 Hz, 1H), 8.03–7.98 (m, 1H), 7.88 (s, 1H), 7.10 (dd, *J* = 7.4, 2.1 Hz, 1H), 6.98 (s, 2H), 3.85 (s, 6H), 3.79 (s, 3H). LC-MS: *m*/*z* = 391.4–393.4 [M+H].

#### 4-(7-Bromoimidazo[1,2-*a*]pyridin-3-yl)-2,6-dimethoxy-benzoic acid (**37**)

Intermediate **36** (2.4 g, 6.13 mmol, 1 equiv) was dissolved in MeOH (35 mL) and THF (35 mL). NaOH 2N (12.3 mL, 24.54 mmol, 4 equiv) was added. The mixture was stirred at 70 °C for 18 h. Another portion of NaOH 2N (6.15 mL, 12.27 mmol, 2 equiv) was added. The mixture was stirred at 70 °C for 18 h and acidified with HCl 2N to pH = 4. Organic solvents were removed by evaporation under reduced pressure, and the precipitate in the remaining water was filtered to afford **37** (2.2 g, 96%). ^1^H NMR (400 MHz, DMSO-*d*_6_) δ ppm 12.87 (s, 1H), 8.63 (d, *J* = 7.4 Hz, 1H), 8.00 (d, *J* = 2.0 Hz, 1H), 7.86 (s, 1H), 7.10 (dd, *J* = 7.4, 2.1 Hz, 1H), 6.95 (s, 2H), 3.85 (s, 6H). LC-MS: *m*/*z* = 377.4–379.3 [M+H].

#### 4-(7-Bromoimidazo[1,2-*a*]pyridin-3-yl)-2,6-dimethoxy-*N*-(2,2,2-trifluoroethyl)benzamide (**38**)

To **37** (5.0 g, 13.25 mmol, 1 equiv) in DMF (98 mL) were added DIPEA (6.6 mL, 39.76 mmol, 3 equiv) and HATU (5.0 g, 13.25 mmol, 1 equiv). The mixture was stirred at room temperature for 20 min. 2,2,2-Trifluoroethylamine hydrochloride (3.6 g, 26.51 mmol, 2 equiv) was added. The mixture was stirred at room temperature for 18 h. The reaction mixture was concentrated *in vacuo*. Saturated aqueous NaHCO_3_ was added, and the mixture was extracted with CH_2_Cl_2_. The combined organic layers were washed with water and brine, dried over anhydrous Na_2_SO_4_, filtered, and concentrated *in vacuo*. The crude product was recrystallized in CH_3_CN twice. The solid was filtered and was then triturated in aqueous NaHCO_3_ and filtered to afford **38** (3.47 g, 57%). ^1^H NMR (400 MHz, DMSO-*d*_6_) δ ppm 8.82 (t, *J* = 6.4 Hz, 1H), 8.59 (d, *J* = 7.4 Hz, 1H), 8.03–7.98 (m, 1H), 7.86 (s, 1H), 7.12 (dd, *J* = 7.4, 2.1 Hz, 1H), 6.95 (s, 2H), 4.07–3.94 (m, 2H), 3.82 (s, 6H). LC-MS: *m*/*z* = 458.5–460.4 [M+H].

#### 2,6-Dimethoxy-4-(7-morpholinoimidazo[1,2-*a*]pyridin-3-yl)-*N*-(2,2,2-trifluoroethyl)benzamide (**11**)

Intermediate **38** was reacted with morpholine according to general procedure A, to afford **11** (18 mg, 36%). ^1^H NMR (400 MHz, CDCl_3_) δ 8.07 (d, *J* = 0.6 Hz, 1H), 7.43 (s, 1H), 6.77 (d, *J* = 2.4 Hz, 1H), 6.61 (s, 2H), 6.59–6.54 (m, 1H), 6.23 (t, *J* = 6.4 Hz, 1H), 4.14–4.01 (m, 2H), 3.85–3.76 (m, 10H), 3.20–3.13 (m, 4H). LC-MS: *m*/*z* = 465.3 [M+H].

#### 4-[7-(3,3-Difluoroazetidin-1-yl)imidazo[1,2-*a*]pyridin-3-yl]-2,6-dimethoxy-*N*-(2,2,2-trifluoroethyl)benzamide (**13**)

Intermediate **38** was reacted with difluoroazetidine hydrochloride salt according to general procedure A, to afford **13** (43 mg, 83%). ^1^H NMR (400 MHz, CDCl_3_) δ ppm 8.16 (d, *J* = 0.7 Hz, 1H), 7.54 (s, 1H), 6.71–6.64 (m, 3H), 6.35 (dd, *J* = 7.5, 2.4 Hz, 1H), 6.23 (t, *J* = 6.4 Hz, 1H), 4.39 (t, *J* = 11.6 Hz, 4H), 4.22–4.09 (m, 2H), 3.88 (s, 6H). LC-MS: *m*/*z* = 471.3 [M+H].

#### 2,6-Dimethoxy-4-[7-(4-methylpiperazin-1-yl)imidazo[1,2-*a*]pyridin-3-yl]-*N*-(2,2,2-trifluoroethyl)benzamide (**15**)

Intermediate **38** was reacted with 1-methylpiperazine according to general procedure A, to afford **15** (37 mg, 71%). ^1^H NMR (400 MHz, methanol-*d*_4_) δ ppm 8.48–8.40 (m, 2H), 7.71 (s, 1H), 7.11 (dd, *J* = 7.9, 2.5 Hz, 1H), 6.92 (s, 2H), 6.89 (d, *J* = 2.5 Hz, 1H), 4.08 (q, *J* = 9.3 Hz, 2H), 3.89 (s, 6H), 3.55 (t, *J* = 5.1 Hz, 4H), 2.83 (t, *J* = 5.1 Hz, 4H), 2.53 (s, 3H). LC-MS: *m*/*z* = 478.3 [M+H].

#### *N*-Cyclopropyl-2-hydroxy-6-methoxy-benzamide (**40**)

At room temperature, 6-methoxysalicylic acid **39** (10 g, 59.5 mmol, 1 equiv) was dissolved in DMF (50 mL), HATU (33.93 g, 89.2 mmol, 1.5 equiv) was added, followed 15 min later by cyclopropylamine (10.18 g, 178.6 mmol, 3 equiv) and DIPEA (34.55 g, 260 mmol, 4.3 equiv). The reaction mixture was stirred at room temperature for 18 h. HATU (22.63 g, 59.5 mmol, 1 equiv), cyclopropylamine (6.74 g, 118.4 mmol, 2 equiv), and DIPEA (15.73 g, 118.4 mmol, 2 equiv) were added. The reaction mixture was stirred at room temperature for 48 h. The reaction mixture was concentrated *in vacuo*. Purification by flash chromatography on silica gel (eluting with heptane/EtOAc 100/0 to 50/50) and then trituration in MeOH/diethyl ether to get rid of an insoluble byproduct (repeated twice) were done. The filtrate was concentrated *in vacuo* to afford **40** (3.5 g, 28%). ^1^H NMR (400 MHz, CDCl_3_) δ ppm 8.32 (s, 1H), 7.21 (t, *J* = 8.4 Hz, 1H), 6.57 (dd, *J* = 8.4, 1.0 Hz, 1H), 6.34 (dd, *J* = 8.4, 1.1 Hz, 1H), 3.87 (s, 3H), 2.84 (tq, *J* = 7.3, 3.8 Hz, 1H), 0.87–0.81 (m, 2H), 0.61–0.55 (m, 2H). LC-MS: *m*/*z* = 208.4 [M+H].

#### *N*-Cyclopropyl-2-(difluoromethoxy)-6-methoxy-benzamide (**41**)

Under an inert atmosphere, **40** (2.80 g, 13.5 mmol, 1 equiv) was dissolved in acetonitrile (20 mL) and cooled to −20 °C. KOH (7.57 g, 135 mmol, 10 equiv) solubilized with water (20 mL) was added, and the mixture was stirred for 10 min, then bromodifluoromethyl diethylphosphonate (10.9 g, 40 mmol, 3 equiv) was added slowly. The reaction mixture was stirred at −20 °C for 30 min then at room temperature for an additional 30 min. Water was added, and 3 extractions with EtOAc were performed. The organic layers were dried with Na_2_SO_4_, filtered, and concentrated *in vacuo*. Purification by flash chromatography on silica gel (eluting with a gradient heptane/EtOAc 100/0 to 0/100) afforded **41** (2.86 g, 79%). ^1^H NMR (400 MHz, DMSO-*d*_6_) δ ppm 8.26 (d, *J* = 4.5 Hz, 1H), 7.38 (t, *J* = 8.4 Hz, 1H), 7.05 (t, *J* = 72.0 Hz, 1H), 6.97–6.92 (m, 1H), 6.78 (dd, *J* = 8.4, 0.9 Hz, 1H), 3.77 (s, 3H), 2.76 (ddt, *J* = 11.4, 7.3, 4.0 Hz, 1H), 0.65 (td, *J* = 7.0, 4.7 Hz, 2H), 0.46–0.41 (m, 2H). LC-MS: *m*/*z* = 258.4 [M+H].

#### *N*-Cyclopropyl-2-(difluoromethoxy)-6-methoxy-4-(4,4,5,5-tetramethyl-1,3,2-dioxaborolan-2-yl)benzamide (**42**)

At room temperature, **41** (47 g, 183 mmol, 1 equiv) and bis(pinacolato)diboron (96 g, 366 mmol, 2 equiv) were dissolved in dry THF (400 mL). The mixture was purged with N_2_ for 15 min. (1,5-Cyclooctadiene)(methoxy)iridium(I) dimer (6.2 g, 9.15 mmol, 0.05 equiv) was added, followed by addition of 2-(4-*tert*-butyl)bipyridyl (2.6 g, 9.15 mmol, 0.05 equiv). The mixture was refluxed and stirred for 6 h then left overnight at room temperature. The solvent was removed under reduced pressure. The residue was dissolved in DCM (300 mL) and quenched slowly with water (300 mL, ***Caution:*** exothermic). The organic layer was separated and concentrated to dryness under reduced pressure. The crude residue was purified by flash chromatography on silica gel (eluting with cyclohexane/EtOAc 80/20 to 60/40). The collected fractions were combined and concentrated *in vacuo*. The solid obtained was further triturated in diisopropyl ether, stirred for 45 min, filtered, and dried *in vacuo* to afford **42**. (34.1 g, 49%). ^1^H NMR (400 MHz, CDCl_3_) δ ppm 7.26–7.18 (m, 2H), 6.61 (t, *J* = 74.5 Hz, 1H), 5.88 (s, 1H), 3.90 (s, 3H), 2.92 (tq, *J* = 7.1, 3.6 Hz, 1H), 1.37 (s, 12H), 0.94–0.80 (m, 2H), 0.69–0.60 (m, 2H). LC-MS: *m*/*z* = 384.2 (M+H of boronic ester), *m*/*z* = 302.1 (M+H of boronic acid).

#### 4-(7-Bromoimidazo[1,2-*a*]pyridin-3-yl)-*N*-cyclopropyl-2-(difluoromethoxy)-6-methoxy-benzamide (**43a**)

A flask was charged with 7-bromo-3-iodo-imidazo[1,2-*a*]pyridine **34** (5.0 g, 15.4 mmol, 1 equiv), **42** (6.5 g, 17.0 mmol, 1.1 equiv), Cs_2_CO_3_ (11.0 g, 30.9 mmol, 2 equiv), and degassed with N_2_ in dioxane/water solvent mixture (4/1, 80 mL). Then, Pd(PPh_3_)_4_ (1.8 g, 0.77 mmol, 0.05 equiv) was added. The mixture was purged with N_2_ and stirred at 90 °C for 20 h. Dioxane was evaporated, aqueous NaHCO_3_ sat was added, and the mixture was extracted with EtOAc. The combined organic layers were washed with water and brine, dried over anhydrous MgSO_4_, filtered, and concentrated *in vacuo*. The solid obtained was triturated with CH_3_CN and filtered to afford **43a** (7 g, 96%). ^1^H NMR (400 MHz, DMSO-*d*_6_) δ ppm 8.56 (d, *J* = 7.3 Hz, 1H), 8.36 (d, *J* = 4.5 Hz, 1H), 8.01 (d, *J* = 2.0 Hz, 1H), 7.88 (s, 1H), 7.22 (t, *J* = 72.0 Hz, 1H), 7.21 (s, 1H), 7.15 (dd, *J* = 7.3, 2.1 Hz, 1H), 7.06 (s, 1H), 3.87 (s, 3H), 2.79 (td, *J* = 7.3, 3.7 Hz, 1H), 0.68 (td, *J* = 7.0, 4.7 Hz, 2H), 0.50–0.44 (m, 2H). LC-MS: *m*/*z* = 450.1–452.1 [M - H].

#### 4-(7-Aminoimidazo[1,2-*a*]pyridin-3-yl)-*N*-cyclopropyl-2-(difluoromethoxy)-6-methoxy-benzamide (**43b**)

To 3-bromoimidazo[1,2-*a*]pyridin-7-amine (200 mg, 0.94 mmol, 1 equiv), **42** (397 mg, 1.03 mmol, 1.1 equiv), Cs_2_CO_3_ (717 mg, 1.88 mmol, 2 equiv) in dioxane/water solvent mixture (4/1, 5 mL) degassed with N_2_ was added Pd(dppf)Cl_2_ (77 mg, 0.1 mmol, 0.1 equiv), and the mixture was purged with N_2_ then stirred at 90 °C for 1 h. Dioxane was evaporated, aqueous NaHCO_3_ sat was added, and the mixture was extracted with EtOAc. The combined organic layers were washed with water and brine, dried over anhydrous MgSO_4_, filtered, and concentrated *in vacuo*. The crude residue was purified by flash chromatography on silica gel (eluting with a gradient of 0 to 10% MeOH in CH_2_Cl_2_) to afford **43b** (240 mg, 65%). LC-MS: *m*/*z* = 389.3 [M+H].

#### *N*-Cyclopropyl-2-(difluoromethoxy)-4-(7-fluoroimidazo[1,2-*a*]pyridin-3-yl)-6-methoxy-benzamide (**43c**)

A solution of 7-fluoroimidazo[1,2-*a*]pyridine (2.0 g, 14.7 mmol, 1 equiv) in dry DMAC (40 mL) was degassed with N_2_, and then **42** (6.0 g, 17.6 mmol, 1.2 equiv), KOAc (4.3 g, 44.1 mmol, 3 equiv), and Pd(dppf)Cl_2_·DCM (600 mg, 0.73 mmol, 0.05 equiv) were added. The mixture was degassed with N_2_ for 10 min and was then stirred at 110 °C for 4 h. The reaction mixture was filtered, and the filtrate was concentrated. The crude material was purified by flash chromatography on silica gel (eluting with a gradient of 0 to 2% MeOH in EtOAc) to give a solid that was triturated in EtOH to afford **43c** (4.4 g, 77%). ^1^H NMR (400 MHz, DMSO-*d*_6_) δ ppm 8.67 (ddd, *J* = 7.6, 5.8, 0.7 Hz, 1H), 8.35 (d, *J* = 4.5 Hz, 1H), 7.84 (s, 1H), 7.57–7.53 (m, 1H), 7.23 (t, *J* = 72.0 Hz, 1H), 7.21 (d, *J* = 1.3 Hz, 1H), 7.07–7.03 (m, 2H), 3.87 (s, 3H), 2.83–2.75 (m, 1H), 0.68 (td, *J* = 7.0, 4.8 Hz, 2H), 0.49–0.44 (m, 2H). LC-MS: *m*/*z* = 392.2 [M+H].

#### *N*-Cyclopropyl-2-(difluoromethoxy)-6-methoxy-4-(7-morpholinoimidazo[1,2-*a*]pyridin-3-yl)benzamide (**12**)

Intermediate **43a** was reacted with morpholine according to general procedure A, to afford **12** (29 mg, 48%). ^1^H NMR (400 MHz, CDCl_3_) δ ppm 8.41 (d, *J* = 4.2 Hz, 1H), 7.68 (s, 1H), 7.17 (d, *J* = 1.3 Hz, 1H), 7.06 (dd, *J* = 9.0, 1.8 Hz, 2H), 6.91 (t, *J* = 73.7 Hz, 1H), 6.84 (d, *J* = 2.6 Hz, 1H), 3.94 (s, 3H), 3.91–3.82 (m, 4H), 3.39 (dd, *J* = 5.9, 3.9 Hz, 4H), 2.87 (tt, *J* = 7.4, 3.9 Hz, 1H), 0.92–0.76 (m, 2H), 0.63 (ddd, *J* = 7.0, 5.1, 3.9 Hz, 2H). LC-MS: *m*/*z* = 459.2 [M+H].

#### *N*-Cyclopropyl-4-(7-(3,3-difluoroazetidin-1-yl)imidazo[1,2-*a*]pyridin-3-yl)-2-(difluoromethoxy)-6-methoxybenzamide (**14**)

Intermediate **43a** was reacted with 3,3-difluoroazetidine hydrochloride according to general procedure A, to afford **14** (18 mg, 35%). ^1^H NMR (400 MHz, DMSO-*d*_6_) δ ppm 8.50 (d, *J* = 7.4 Hz, 1H), 8.34 (d, *J* = 4.5 Hz, 1H), 8.14 (s, 1H), 7.68 (s, 1H), 7.22 (t, *J* = 74 Hz, 1H), 7.13 (d, *J* = 1.3 Hz, 1H), 6.97 (s, 1H), 6.60–6.51 (m, 2H), 4.43 (t, *J* = 12.2 Hz, 4H), 3.87 (s, 3H), 2.84–2.73 (m, 1H), 0.73–0.61 (m, 2H), 0.50–0.38 (m, 2H). LC-MS: *m*/*z* = 465.4 [M+H].

#### *N*-Cyclopropyl-2-(difluoromethoxy)-6-methoxy-4-(7-(4-methylpiperazin-1-yl)imidazo[1,2-*a*]pyridin-3-yl)benzamide (**16**)

Intermediate **43a** was reacted with *N*-methylpiperazine according to general procedure A, to afford **16** (20 mg, 19%). ^1^H NMR (400 MHz, methanol-*d*_4_) δ 8.37 (d, *J* = 7.8 Hz, 1H), 7.59 (s, 1H), 7.13 (d, *J* = 1.3 Hz, 1H), 7.04–7.00 (m, 1H), 6.97 (dd, *J* = 7.8, 2.5 Hz, 1H), 6.91 (t, *J* = 75 Hz, 1H), 6.80 (d, *J* = 2.5 Hz, 1H), 3.93 (s, 3H), 3.40 (t, *J* = 5.1 Hz, 4H), 2.86 (hept, *J* = 3.9 Hz, 1H), 2.67 (t, *J* = 5.1 Hz, 4H), 2.40 (s, 3H), 0.87–0.76 (m, 2H), 0.67–0.58 (m, 2H). LC-MS: *m*/*z* = 472.4 [M+H].

#### 4-(7-(4-Acetylpiperazin-1-yl)imidazo[1,2-*a*]pyridin-3-yl)-*N*-cyclopropyl-2-(difluoromethoxy)-6-methoxybenzamide (**17**)

To a solution of **43a** (50 mg, 0.11 mmol, 1 equiv) in dioxane (1 mL) was added 1-piperazin-1-ylethanone (17 mg, 0.133 mmol, 1.2 equiv), RuPhos (10 mg, 0.011 mmol, 0.1 equiv), RuPhos Pd G2 (13 mg, 0.022 mmol, 0.2 equiv), and K_3_PO_4_ (117 mg, 0.553 mmol, 5 equiv). The reaction mixture was stirred at 90 °C for 7 h then filtered, and the filtrate was purified by preparative LC-MS. The recovered material was crystallized in hot ACN then rinsed with ACN and Et_2_O to give **17** (20 mg, 19%). ^1^H NMR (400 MHz, CDCl_3_) δ ppm 8.12 (d, *J* = 7.7 Hz, 1H), 7.55 (s, 1H), 6.98–6.94 (m, 1H), 6.92–6.88 (m, 2H), 6.73–6.67 (m, 1H), 6.63 (t, *J* = 74.3 Hz, 1H), 6.10 (d, *J* = 3.1 Hz, 1H), 3.89 (s, 3H), 3.80 (t, *J* = 5.2 Hz, 2H), 3.66 (t, *J* = 5.2 Hz, 2H), 3.33–3.24 (m, 4H), 2.93 (tq, *J* = 7.2, 3.7 Hz, 1H), 2.16 (s, 3H), 0.91–0.84 (m, 2H), 0.68–0.60 (m, 2H). LC-MS: *m*/*z* = 500.5 [M+H].

#### *N*-Cyclopropyl-2-(difluoromethoxy)-4-(7-(4-(2-hydroxyethyl)piperazin-1-yl)imidazo[1,2-*a*]pyridin-3-yl)-6-methoxybenzamide (**18**)

To a solution of **43a** (50 mg, 0.11 mmol, 1 equiv) in dioxane (1 mL) was added 1-2-(piperazin-1-yl)ethan-1-ol (17 mg, 0.133 mmol, 1.2 equiv), RuPhos (10 mg, 0.011 mmol, 0.1 equiv), RuPhos Pd G2 (13 mg, 0.022 mmol, 0.2 equiv), and K_3_PO_4_ (117 mg, 0.553 mmol, 5 equiv). The reaction mixture was stirred at 90 °C for 7 h then filtered, and the filtrate was purified by preparative HPLC. The recovered material was crystallized in hot ACN then rinsed with ACN and Et_2_O to give **18** (13 mg, 24%). ^1^H NMR (400 MHz, DMSO-*d*_6_) δ ppm 8.39 (d, *J* = 7.8 Hz, 1H), 8.33 (d, *J* = 4.5 Hz, 1H), 7.64 (s, 1H), 7.22 (t, *J* = 74.0 Hz, 1H), 7.12 (d, *J* = 1.3 Hz, 1H), 6.97 (d, *J* = 1.3 Hz, 1H), 6.93 (dd, *J* = 7.8, 2.6 Hz, 1H), 6.77 (d, *J* = 2.5 Hz, 1H), 4.45 (t, *J* = 5.3 Hz, 1H), 3.86 (s, 3H), 3.54 (q, *J* = 5.9 Hz, 2H), 3.29–3.23 (m, 4H), 2.80–2.74 (m, 1H), 2.57 (t, *J* = 5.1 Hz, 4H), 2.44 (t, *J* = 6.2 Hz, 2H), 0.70–0.64 (m, 2H), 0.50–0.42 (m, 2H). LC-MS: *m*/*z* = 502.3 [M+H].

#### *N*-Cyclopropyl-2-(difluoromethoxy)-4-[7-[4-(dimethylamino)-1-piperidyl]imidazo[1,2-*a*]pyridin-3-yl]-6-methoxy-benzamide (**19**)

Intermediate **43a** was reacted with *N,N-*dimethylpiperidin-4-amine according to general procedure A, to afford **19** (7 mg, 14%). ^1^H NMR (400 MHz, DMSO-*d*_6_) δ 8.38 (d, *J* = 7.8 Hz, 1H), 8.33 (d, *J* = 4.5 Hz, 1H), 7.63 (s, 1H), 7.22 (t, *J* = 74.0 Hz, 1H), 7.13 (d, *J* = 1.3 Hz, 1H), 6.97 (d, *J* = 1.2 Hz, 1H), 6.92 (dd, *J* = 7.8, 2.6 Hz, 1H), 6.77 (d, *J* = 2.4 Hz, 1H), 3.87 (s, 5H), 2.80 (ddt, *J* = 11.2, 7.2, 2.7 Hz, 3H), 2.28 (tt, *J* = 10.9, 3.6 Hz, 1H), 2.19 (s, 6H), 1.90–1.81 (m, 2H), 1.47 (qd, *J* = 12.0, 3.8 Hz, 2H), 0.68 (td, *J* = 7.1, 4.8 Hz, 2H), 0.50–0.43 (m, 2H). LC-MS: *m*/*z* = 500.4 [M+H].

#### *N*-Cyclopropyl-2-(difluoromethoxy)-4-(7-(3-(dimethylamino)azetidin-1-yl)imidazo[1,2-*a*]pyridin-3-yl)-6-methoxybenzamide (**20**)

Intermediate **43a** was reacted with *N,N*-dimethylazetidin-3-amine hydrochloride according to general procedure A, to afford **20** (22 mg, 42%). ^1^H NMR (400 MHz, methanol-*d*_4_) δ ppm 8.32 (dd, *J* = 7.5, 0.7 Hz, 1H), 7.50 (s, 1H), 7.09 (d, *J* = 1.3 Hz, 1H), 6.97 (q, *J* = 1.1 Hz, 1H), 6.97 (d, *J* = 74.5 Hz, 1H), 6.48 (dd, *J* = 7.5, 2.4 Hz, 1H), 6.32 (d, *J* = 2.3 Hz, 1H), 4.16–4.08 (m, 2H), 3.91 (s, 3H), 3.81 (dd, *J* = 8.0, 5.3 Hz, 2H), 3.37–3.32 (m, 1H), 2.89–2.82 (m, 1H), 2.26 (s, 6H), 0.85–0.77 (m, 2H), 0.63–0.58 (m, 2H). LC-MS: *m*/*z* = 472.5 [M+H].

#### *N*-Cyclopropyl-2-(difluoromethoxy)-6-methoxy-4-(7-((3-morpholinophenyl)amino)imidazo[1,2-*a*]pyridin-3-yl)benzamide (**21**)

Under inert atmosphere, to a solution of **43a** (50 mg, 0.11 mmol, 1 equiv) in dioxane (1.5 mL) was added 3-morpholinoaniline (30 mg, 0.166 mmol, 1.5 equiv), Xantphos (19 mg, 0.033 mmol, 0.3 equiv), Pd_2_(dba)_3_ (10 mg, 0.011 mmol, 0.1 equiv), and Cs_2_CO_3_ (108 mg, 0.332 mmol, 3 equiv). The reaction mixture was stirred at 90 °C for 3 h in a sealed vial, then quenched with water, extracted with AcOEt (twice). The combined layers were passed on a phase separator and concentrated under reduced pressure. The residue was purified by preparative HPLC to give **21** (26 mg, 43%). ^1^H NMR (400 MHz, DMSO-*d*_6_) δ ppm 8.58 (s, 1H), 8.47 (d, *J* = 7.5 Hz, 1H), 8.33 (d, *J* = 4.5 Hz, 1H), 7.62 (s, 1H), 7.24 (t, *J* = 73.9 Hz, 1H), 7.20 (t, *J* = 8.0 Hz, 1H), 7.12 (d, *J* = 1.3 Hz, 1H), 6.99–6.95 (m, 2H), 6.77 (dd, *J* = 7.6, 2.4 Hz, 1H), 6.75–6.69 (m, 2H), 6.66–6.60 (m, 1H), 3.88 (s, 3H), 3.77–3.71 (m, 4H), 3.14–3.07 (m, 4H), 2.82–2.74 (m, 1H), 0.70–0.64 (m, 2H), 0.48–0.43 (m, 2H). LC-MS: *m*/*z* = 550.4 [M+H].

#### *N*-Cyclopropyl-2-(difluoromethoxy)-6-methoxy-4-(7-((1-methyl-1*H*-imidazol-4-yl)amino)imidazo[1,2-*a*]pyridin-3-yl)benzamide (**22**)

Under inert atmosphere, to a solution of **35a** (50 mg, 0.11 mmol, 1 equiv) in dioxane(1.5 mL) was added 1-methylimidazol-4-amine (16 mg, 0.166 mmol, 1.5 equiv), Xantphos (19 mg, 0.033 mmol, 0.3 equiv), Pd_2_(dba)_3_ (10 mg, 0.011 mmol, 0.1 equiv), and Cs_2_CO_3_ (108 mg, 0.33 mmol, 3 equiv). The mixture was stirred at 90 °C for 3 h in a sealed vial, then quenched with water, extracted with AcOEt (twice). The combined layers were passed on a phase separator and concentrated under reduced pressure. The residue was purified by preparative HPLC to give **22** as formic acid salt (18 mg, 35%). ^1^H NMR (400 MHz, DMSO-*d*_6_) δ ppm 8.81 (s, 1H), 8.40 (d, *J* = 7.5 Hz, 1H), 8.33 (d, *J* = 4.5 Hz, 1H), 8.14 (s, 1H), 7.60 (s, 1H), 7.46 (d, *J* = 1.4 Hz, 1H), 7.22 (t, *J* = 74.0 Hz, 1H), 7.11 (d, *J* = 1.3 Hz, 1H), 7.06 (d, *J* = 2.3 Hz, 1H), 6.96 (dd, *J* = 5.0, 1.3 Hz, 2H), 6.83 (dd, *J* = 7.6, 2.4 Hz, 1H), 3.87 (s, 3H), 3.66 (s, 3H), 2.81–2.75 (m, 1H), 0.72–0.64 (m, 2H), 0.50–0.43 (m, 2H). LC-MS: *m*/*z* = 469.3 [M+H].

#### *N*-(3-(4-(Cyclopropylcarbamoyl)-3-(difluoromethoxy)-5-methoxyphenyl)imidazo[1,2-*a*]pyridin-7-yl)tetrahydro-2*H*-pyran-4-carboxamide (**23**)

To a solution of **43a** (60 mg, 0.13 mmol, 1 equiv) in dioxane (1 mL) was added tetrahydropyran-4-carboxamide (34 mg, 0.26 mmol, 2 equiv) Pd(OAc)_2_ (3 mg, 0.013 mmol, 0.1 equiv), Xantphos (15 mg, 0.026 mmol, 0.2 equiv), and Cs_2_CO_3_ (100 mg, 0.26 mmol, 2 equiv). The reaction mixture was heated in a microwave oven at 150 °C for 1 h, then quenched with saturated NaHCO_3_, extracted with AcOEt (3 times). The combined layers were dried over Na_2_SO_4_, filtered, and concentrated under reduced pressure. The residue was purified by preparative HPLC to give **23** (30 mg, 45%). ^1^H NMR (400 MHz, DMSO-*d*_6_) δ ppm 9.42 (s, 1H), 7.74 (dd, *J* = 7.5, 0.9 Hz, 1H), 7.52 (d, *J* = 4.5 Hz, 1H), 7.25 (dd, *J* = 2.2, 0.8 Hz, 1H), 6.92 (s, 1H), 6.40 (t, *J* = 73.9 Hz, 1H), 6.32 (d, *J* = 1.3 Hz, 1H), 6.28 (dd, *J* = 7.6, 2.2 Hz, 1H), 6.17 (d, *J* = 1.2 Hz, 1H), 3.13–3.06 (m, 2H), 3.04 (s, 3H), 2.58–2.52 (m, 2H), 1.99–1.90 (m, 1H), 1.85–1.76 (m, 1H), 0.95–0.78 (m, 4H), −0.13 – −0.21 (m, 2H), −0.34 – −0.41 (m, 2H). LC-MS: *m*/*z* = 501.3 [M+H].

#### *N*-Cyclopropyl-2-(difluoromethoxy)-6-methoxy-4-(7-(3-methoxypropanamido)imidazo[1,2-*a*]pyridin-3-yl)benzamide (**24**)

To a solution of **43b** (30 mg, 0.07 mmol, 1 equiv) in DCM (1 mL) were added 3-methoxypropionic acid (16 mg, 0.15 mmol, 2 equiv) and Ghosez’s reagent (31 mg, 0.23 mmol, 3 equiv). The mixture was stirred at room temperature for 4 days. The mixture was quenched with NaHCO_3_ sat and extracted with DCM. The combined layers were dried over Na_2_SO_4_, filtered, and evaporated under reduced pressure. The residue was purified by flash chromatography on silica gel eluting with a gradient DCM/MeOH (99/1 to 96/4) to afford **24** (14 mg, 38%). ^1^H NMR (400 MHz, methanol-*d*_4_) δ ppm δ 8.48 (d, *J* = 7.6 Hz, 1H), 8.17–8.11 (m, 1H), 7.69 (s, 1H), 7.18 (dd, *J* = 7.5, 1.8 Hz, 1H), 7.15 (d, *J* = 1.3 Hz, 1H), 7.03 (q, *J* = 1.0 Hz, 1H), 6.91 (t, *J* = 73.7 Hz, 1H), 3.93 (s, 3H), 3.74 (t, *J* = 6.0 Hz, 2H), 3.37 (s, 3H), 2.85 (tt, *J* = 7.3, 3.8 Hz, 1H), 2.67 (t, *J* = 6.0 Hz, 2H), 0.82 (td, *J* = 7.1, 5.1 Hz, 2H), 0.64–0.57 (m, 2H). LC-MS: *m*/*z* = 475.3 [M+H].

#### *N*-Cyclopropyl-2-(difluoromethoxy)-6-methoxy-4-(7-((2-morpholinoethyl)amino)imidazo[1,2-*a*]pyridin-3-yl)benzamide (**25**)

In a sealed tube under inert atmosphere, to a solution of **43a** (50 mg, 0.11 mmol, 1 equiv) in dioxane (1.5 mL) were added 2-morpholinoethanamine (22 mg, 0.17 mmol, 1.5 equiv), Cs_2_CO_3_ (108 mg, 0.33 mmol, 3 equiv), Pd_2_(dba)_3_ (10 mg, 0.011 mmol, 0.1 equiv), and XantPhos (19 mg, 0.033 mmol, 0.3 equiv). The reaction mixture was stirred at 90 °C until completion of the reaction (monitored by HPLC). The reaction mixture was evaporated and partitioned between DCM and water, the aqueous phase was extracted with DCM on a phase separator, and the organic layer was concentrated under reduced pressure. The residue was purified by preparative HPLC to afford **25** (14 mg, 25%). ^1^H NMR (400 MHz, DMSO-*d*_6_) δ ppm 8.33–8.26 (m, 2H), 7.51 (s, 1H), 7.21 (t, *J* = 74.0 Hz, 1H), 7.07 (d, *J* = 1.3 Hz, 1H), 6.91 (d, *J* = 1.2 Hz, 1H), 6.58 (dd, *J* = 7.6, 2.3 Hz, 1H), 6.34 (d, *J* = 2.3 Hz, 1H), 6.26 (t, *J* = 5.3 Hz, 1H), 3.86 (s, 3H), 3.60 (t, *J* = 4.6 Hz, 4H), 3.23–3.18 (m, 2H), 2.81–2.73 (m, 1H), 2.54 (t, *J* = 6.6 Hz, 2H), 2.44 (t, *J* = 4.6 Hz, 4H), 0.70–0.63 (m, 2H), 0.48–0.43 (m, 2H). LC-MS: *m*/*z* = 502.5 [M+H].

#### *N*-Cyclopropyl-2-(difluoromethoxy)-6-methoxy-4-(7-(2-morpholinoethoxy)imidazo[1,2-*a*]pyridin-3-yl)benzamide (**26**)

To a solution of 2-morpholin-4-ylethanol (150 μL, 1.02 mmol, 10 equiv) in DMF (1 mL) was added NaH 60% in oil (16 mg, 0.41 mmol, 4 equiv). The reaction was stirred for 10 min at room temperature then **43c** (40 mg, 0.10 mmol, 1 equiv) was added. The mixture was stirred at room temperature overnight. The solvent was evaporated. The residue was purified by flash chromatography on silica gel eluting with a gradient DCM/MeOH (99/1 to 93/7 to afford **26** (38 mg, 74%). ^1^H NMR (400 MHz, CDCl_3_) δ ppm 8.10 (d, *J* = 7.6 Hz, 1H), 7.56 (s, 1H), 6.99–6.95 (m, 2H), 6.91–6.89 (m, 1H), 6.66–6.63 (m, 1H), 6.63 (d, *J* = 74.0 Hz, 1H), 6.06 (d, *J* = 3.2 Hz, 1H), 4.20 (t, *J* = 5.5 Hz, 2H), 3.89 (s, 3H), 3.79–3.72 (m, 4H), 2.97–2.90 (m, 1H), 2.87 (t, *J* = 5.5 Hz, 2H), 2.64–2.58 (m, 4H), 0.92–0.83 (m, 2H), 0.69–0.60 (m, 2H). LC-MS: *m*/*z* = 503.4 [M+H].

#### *N*-Cyclopropyl-2-(difluoromethoxy)-6-methoxy-4-[7-(2-tetrahydropyran-4-ylethoxy)imidazo[1,2-*a*]pyridin-3-yl]benzamide (**27**)

To a solution of 2-tetrahydropyran-4-ylethanol (50 mg, 0.38 mmol, 5 equiv) in DMF (1 mL) was added NaH 60% in oil (15 mg, 0.38 mmol, 5 equiv). The reaction was stirred for 10 min at room temperature then **43c** (30 mg, 0.077 mmol, 1 equiv) was added. The mixture was stirred at room temperature overnight. The solvent was evaporated. The residue was purified by preparative HPLC to afford **27** (23 mg, 60%). ^1^H NMR (400 MHz, DMSO-*d*_6_) δ 8.85 (t, *J* = 6.4 Hz, 1H), 8.53 (s, 1H), 7.62 (d, *J* = 8.9 Hz, 1H), 7.26 (d, *J* = 2.3 Hz, 1H), 7.12 (dd, *J* = 9.0, 2.3 Hz, 1H), 6.97 (s, 2H), 4.01 (qd, *J* = 9.7, 6.3 Hz, 2H), 3.83 (s, 6H), 3.81–3.73 (m, 4H), 3.16–3.09 (m, 4H). LC-MS: *m*/*z* = 502.5 [M+H].

#### 4-(2-Imidazo[1,2-*a*]pyridin-7-yloxyethyl)morpholine (**45**)

To a solution of 2-morpholinoethanol (1.6 mL, 13.2 mmol, 6 equiv) in DMF (22 mL) was added NaH (60% dispersion in mineral oil, 529 mg, 13.2 mmol, 6 equiv) portionwise. After stirring for 30 min at room temperature, 7-fluoroimidazo[1,2-*a*]pyridine (**44**, 300 mg, 2.2 mmol, 1 equiv) was added, and the mixture was stirred at room temperature for 20 h. The reaction mixture was quenched with a saturated aqueous NaHCO_3_ solution, diluted with EtOAc, and stirred at room temperature for 15 min. The solid was filtered, and the filtrate was concentrated under reduced pressure. The obtained residue was triturated with pentane 3 times. The solid obtained was purified by flash chromatography on silica gel (eluting with a gradient MeOH: 0 to 10% in DCM) to afford **45** (545 mg, 100%). ^1^H NMR (400 MHz, methanol-*d*_4_) δ ppm 8.27 (dd, *J* = 7.5, 0.7 Hz, 1H), 7.65 (dd, *J* = 1.5, 0.7 Hz, 1H), 7.40 (d, *J* = 1.5 Hz, 1H), 6.89 (d, *J* = 2.5 Hz, 1H), 6.66 (dd, *J* = 7.4, 2.5 Hz, 1H), 4.23 (t, *J* = 5.4 Hz, 2H), 3.77–3.66 (m, 4H), 2.87 (t, *J* = 5.4 Hz, 2H), 2.66–2.59 (m, 4H). LC-MS: *m*/*z* = 248.2 [M+H].

#### 5-Bromo-*N*-cyclopropyl-3-methoxy-pyridine-2-carboxamide (**47a**)

To a solution of 5-bromo-3-methoxypicolinic acid **46** (60 mg, 0.26 mmol, 1 equiv) in anhydrous DMF (1 mL) were added DIPEA (68 μL, 0.39 mmol, 1.5 equiv) and HATU (108 mg, 0.28 mmol, 1.1 equiv). The reaction medium was stirred at room temperature for 1 h, and cyclopropylamine (22 μL, 0.31 mmol, 1.2 equiv) was added. The reaction mixture was stirred at room temperature for 20 h, then solvents were evaporated. The residue was diluted with DCM, washed with a 1N aqueous solution of NaOH, and passed through a phase separator. The filtrate was concentrated *in vacuo*, and the crude was purified by flash chromatography on silica gel (eluting with heptane/EtOAc 8/2 to 1/9) to afford **47a** (46 mg, 65%). LC-MS *m*/*z* = 271.1–273.2 [M+H].

#### Methyl 5-bromo-3-(difluoromethoxy)picolinate (**49**)

Methyl 5-bromo-3-hydroxypicolinate **48** (50 mg, 0.215 mmol, 1 equiv), sodium chlorodifluoroacetate (35 mg, 0.26 mmol, 1.2 equiv), and K_2_CO_3_ (60 mg, 0.63 mmol, 3 equiv) were mixed in CH_3_CN (1.5 mL), and the reaction mixture was stirred at reflux for 2 h. The reaction mixture was quenched with a saturated aqueous NaHCO_3_ solution and ice. The mixture was extracted with DCM and then EtOAc. The combined organic layers are dried over Na_2_SO_4_, filtered, and concentrated. The crude material was purified by flash chromatography on silica gel (eluting with EtOAc in heptane) to afford **49** (34 mg, 56%). ^1^H NMR (400 MHz, CDCl_3_) δ ppm 8.65 (d, *J* = 1.9 Hz, 1H), 7.87 (dt, *J* = 1.9, 1.0 Hz, 1H), 6.65 (t, *J* = 73.1 Hz, 1H), 4.00 (s, 3H). LC-MS *m*/*z* = 282.1–284.1 [M+H].

#### 5-Bromo-3-(difluoromethoxy)pyridine-2-carboxylic acid (**50**)

To a solution of intermediate **49** (30 mg, 0.11 mmol, 1 equiv) in THF (1 mL) and water (1 mL) was added LiOH·H_2_O (14 mg, 0.32 mmol, 3 equiv), and the reaction mixture was stirred at room temperature for 2 h. THF was evaporated, and the suspension was diluted with water. The mixture was acidified to pH 2 with a 2N HCl aqueous solution and was then extracted with EtOAc. The combined organic layers were dried over Na_2_SO_4_, filtered, and concentrated to afford **50** (24 mg, 85%). ^1^H NMR (400 MHz, CDCl_3_) δ ppm 8.60 (d, *J* = 1.8 Hz, 1H), 7.99 (dt, *J* = 1.9, 0.9 Hz, 1H), 6.79 (t, *J* = 73.0 Hz, 1H). LC-MS *m*/*z* = 268.0–270.0 [M+H].

#### 5-Bromo-*N*-cyclopropyl-3-(difluoromethoxy)pyridine-2-carboxamide (**47b**)

To a solution of **50** (24 mg, 0.09 mmol, 1 equiv) in DMF (0.5 mL) were added HATU (38 mg, 0.10 mmol, 1.1 equiv) and DIPEA (22 μL, 0.13 mmol, 1.5 equiv), and the mixture was stirred at room temperature for 15 min. Then, cyclopropylamine (6 mg, 0.11 mmol, 1.2 equiv) was added, and the reaction mixture was stirred at room temperature for 17 h. The reaction was hydrolyzed with a saturated aqueous NaHCO_3_ solution and extracted with EtOAc. The combined organic layers were dried over Na_2_SO_4_, filtered, and concentrated. The residue was purified by flash chromatography on silica gel (eluting with heptane/EtOAc 9/1 to 7/3) to give **47b** (15 mg, 54%). ^1^H NMR (400 MHz, CDCl_3_) δ ppm 8.49 (d, *J* = 1.9 Hz, 1H), 7.84 (dt, *J* = 1.8, 0.8 Hz, 1H), 7.74 (s, 1H), 6.86 (t, *J* = 75.4 Hz, 1H), 2.88 (tq, *J* = 7.4, 3.8 Hz, 1H), 0.95–0.79 (m, 2H), 0.73–0.57 (m, 2H). LC-MS *m*/*z* = 307.1–309.1 [M+H].

#### 5-Bromo-3-methoxy-*N*-(2,2,2-trifluoroethyl)pyridine-2-carboxamide (**47c**)

To a solution of **46** (500 mg, 2.15 mmol, 1 equiv) in anhydrous DMF (8.3 mL) were added DIPEA (563 μL, 3.23 mmol, 1.5 equiv) and HATU (901 mg, 2.37 mmol, 1.1 equiv). The mixture was stirred at room temperature for 30 min, and 2,2,2-trifluoroethanamine hydrochloride (350 mg, 2.59 mmol) was added. The reaction mixture was stirred at room temperature for 20 h and was then evaporated to dryness. The residue was diluted with DCM, and a precipitate was formed. The solid was filtered, and the filtrate was concentrated *in vacuo* and purified by flash chromatography on silica gel (eluting with heptane/EtOAc 1/0 to 1/1) to afford **47c** (562 mg, 83%). ^1^H NMR (400 MHz, CDCl_3_) δ ppm 8.27 (d, *J* = 1.7 Hz, 1H), 8.07 (s, 1H), 7.58 (d, *J* = 1.7 Hz, 1H), 4.11 (qd, *J* = 9.1, 6.6 Hz, 2H), 4.00 (s, 3H). LC-MS: *m*/*z* = 313.2–315.2 [M+H].

#### *N*-Cyclopropyl-3-methoxy-5-[7-(2-morpholinoethoxy)imidazo[1,2-*a*]pyridin-3-yl]pyridine-2-carboxamide (**28**)

Under an inert atmosphere, **45** (45 mg, 0.18 mmol, 1.1 equiv), **47a** (45 mg, 0.17 mmol, 1 equiv), KOAc (33 mg, 0.33 mmol, 2 equiv), and Pd(dppf)Cl_2_·DCM adduct (7 mg, 0.008 mmol, 0.05 equiv) were suspended in dry DMAC (1.7 mL). The mixture was degassed with N_2_ and stirred at 110 °C for 1.5 h. Then, additional Pd(dppf)Cl_2_·DCM adduct (7 mg, 0.008 mmol, 0.05 equiv) was added, and the reaction mixture was stirred at 110 °C overnight. The reaction mixture was concentrated, and the crude residue was purified by flash chromatography on silica gel (eluting with MeOH, 0 to 10% in AcOEt) and preparative HPLC to afford **28** (17 mg, 23%). ^1^H NMR (400 MHz, methanol-*d*_4_) δ ppm 8.48 (dd, *J* = 7.6, 0.7 Hz, 1H), 8.41 (d, *J* = 1.7 Hz, 1H), 7.79 (d, *J* = 1.7 Hz, 1H), 7.73 (s, 1H), 7.00 (dd, *J* = 2.6, 0.7 Hz, 1H), 6.79 (dd, *J* = 7.6, 2.5 Hz, 1H), 4.27 (t, *J* = 5.4 Hz, 2H), 4.01 (s, 3H), 3.76–3.69 (m, 4H), 2.95–2.84 (m, 3H), 2.65–2.60 (m, 4H), 0.89–0.81 (m, 2H), 0.69–0.62 (m, 2H). LC-MS *m*/*z* = 438.3 [M+H].

#### *N*-Cyclopropyl-3-(difluoromethoxy)-5-[7-(2-morpholinoethoxy)imidazo[1,2-*a*]pyridin-3-yl]pyridine-2-carboxamide (**29**)

Under an inert atmosphere, **45** (12 mg, 0.018 mmol, 1 equiv) and **47b** (15 mg, 0.048 mmol, 1 equiv) were suspended in dry DMAC (0.5 mL), the mixture was degassed under N_2_, and KOAc (14 mg, 0.14 mmol, 3 equiv) and Pd(dppf)Cl_2_·DCM adduct (2 mg, 0.0024 mmol, 0.05 equiv) were added. Then, the medium was degassed again with N_2_, and the mixture was stirred at 110 °C for 2 h. The reaction medium was concentrated, and the crude residue was purified by flash chromatography on silica gel (eluting with AcOEt/MeOH 99/1 to 80/20) to afford **29** (12 mg, 55%). ^1^H NMR (400 MHz, methanol-*d*_4_) δ ppm 8.75 (d, *J* = 1.8 Hz, 1H), 8.46 (d, *J* = 7.6 Hz, 1H), 8.02–7.95 (m, 1H), 7.77 (s, 1H), 7.04 (t, *J* = 74.1 Hz, 1H), 7.02 (d, *J* = 2.5 Hz, 1H), 6.82 (dd, *J* = 7.6, 2.5 Hz, 1H), 4.28 (t, *J* = 5.4 Hz, 2H), 3.77–3.70 (m, 4H), 2.93–2.85 (m, 3H), 2.70–2.61 (m, 4H), 0.91–0.83 (m, 2H), 0.73–0.64 (m, 2H). LC-MS *m*/*z* = 474.2 [M+H].

#### 3-Methoxy-5-[7-(2-morpholinoethoxy)imidazo[1,2-*a*]pyridin-3-yl]-*N*-(2,2,2-trifluoroethyl)pyridine-2-carboxamide (**30**)

Under an inert atmosphere, **45** (32 mg, 0.13 mmol, 1 equiv), **47c** (40 mg, 0.13 mmol, 1 equiv), KOAc (25 mg, 0.26 mmol, 2 equiv), and Pd(dppf)Cl_2_·DCM adduct (10 mg, 0.013 mmol, 0.1 equiv) were suspended in dry DMAC (1.3 mL). The mixture was degassed with N_2_ and was stirred at 110 °C overnight. The reaction mixture was concentrated, and the crude residue was purified by preparative HPLC and by flash chromatography on silica gel (eluting with MeOH, 0 to 3% in DCM) to afford **30** (20 mg, 33%). ^1^H NMR (400 MHz, methanol-*d*_4_) δ ppm 8.51 (d, *J* = 7.6 Hz, 1H), 8.48 (d, *J* = 1.7 Hz, 1H), 7.84 (d, *J* = 1.7 Hz, 1H), 7.77 (s, 1H), 7.01 (d, *J* = 2.5 Hz, 1H), 6.80 (dd, *J* = 7.5, 2.5 Hz, 1H), 4.27 (t, *J* = 5.4 Hz, 2H), 4.14 (q, *J* = 9.3 Hz, 2H), 4.03 (s, 3H), 3.76–3.70 (m, 4H), 2.88 (t, *J* = 5.4 Hz, 2H), 2.67–2.59 (m, 4H). LC-MS *m*/*z* = 480.4 [M+H].

#### 6-Bromo-2-cyclopropyl-8-fluoro-3,4-dihydroisoquinolin-1-one (**52**)

To a solution of 6-bromo-8-fluoro-3,4-dihydroisoquinolin-1(2H)-one **51** (500 mg, 2.05 mmol, 1 equiv) in THF (20.5 mL) were added TEA (1.43 mL, 10.24 mmol, 5 equiv), pyridine (1.33 mL, 16.39 mmol, 8 equiv) Cu(OAc)_2_ (818 mg, 4.10 mmol, 2 equiv), and cyclopropyl boronic acid (528 mg, 6.15 mmol, 3 equiv). The reaction mixture was stirred at 70 °C for 18 h. Cyclopropyl boronic acid (409 mg, 2.05 mmol, 1 equiv) was added, and the mixture was stirred at 70 °C for 2 h. The mixture was quenched with a saturated aqueous NaHCO_3_ solution and extracted with AcOEt. The combined organic layers were dried over MgSO_4_, filtered, and concentrated *in vacuo*. The crude was purified by flash chromatography gradient heptane/AcOEt (1/0 to 4/6) to afford **52** (207 mg, 36%). LC-MS *m*/*z* = 284.2–286.2 [M+H].

#### 6-Bromo-2-cyclopropyl-8-methoxy-3,4-dihydroisoquinolin-1-one (**53a**)

To a solution of **52** (58 mg, 0.25 mmol, 1 equiv) in THF (0.58 mL) at room temperature was added dropwise MeONa (25% in MeOH, 65 μL, 0.29 mmol, 1.2 equiv), and the suspension was stirred for 1.5 h. The reaction was quenched with a saturated aqueous NH_4_Cl solution, and the THF was evaporated *in vacuo*. The aqueous phase was extracted with CH_2_Cl_2_, the organic layer was dried over MgSO_4_, filtered, and concentrated to afford **53a** (54 mg, 77%). ^1^H NMR (400 MHz, methanol-*d*_4_) δ ppm 7.17 (d, *J* = 1.8 Hz, 1H), 7.06 (dt, *J* = 1.7, 0.8 Hz, 1H), 3.87 (s, 3H), 3.50 (dd, *J* = 7.0, 5.8 Hz, 2H), 2.88 (dtd, *J* = 8.1, 6.7, 3.9 Hz, 3H), 0.89 (tdd, *J* = 6.7, 4.8, 0.7 Hz, 2H), 0.87–0.69 (m, 2H). LC-MS *m*/*z* = 296.3–298.2 [M+H].

#### 6-Bromo-8-methoxy-2-(2,2,2-trifluoroethyl)-3,4-dihydroisoquinolin-1-one (**53b**)

To a stirred solution of 6-bromo-8-methoxy-3,4-dihydroisoquinolin-1(2*H*)-one **54** (10 g, 39.05 mmol, 1 equiv) in THF (240 mL) at 0 °C was added dropwise a solution of LiHMDS 1N in THF (59 mL, 58.57 mmol, 1.5 equiv). The resulting mixture was stirred for 45 min at 0 °C, and 2,2,2-trifluoroethyl trifluoromethanesulfonate (8.44 mL, 58.57 mmol, 1.5 equiv) was added at 0 °C. The reaction mixture was allowed to warm to room temperature and stirred for 22 h. The reaction mixture was quenched with water, THF was evaporated, and the aqueous layer was extracted with EtOAc. The combined organic layers were washed with brine, dried over anhydrous Na_2_SO_4_, filtered, and concentrated *in vacuo*. The residue was purified by flash chromatography on silica gel (eluting with DCM/MeOH 100/0 to 99/1) to afford **53b** (9.98 g, 76%). ^1^H NMR (400 MHz, DMSO-*d*_6_) δ ppm 7.20 (d, *J* = 1.8 Hz, 1H), 7.13 (d, *J* = 1.9 Hz, 1H), 4.30 (q, *J* = 9.7 Hz, 2H), 3.80 (s, 3H), 3.56 (t, *J* = 6.2 Hz, 2H), 2.90 (t, *J* = 6.1 Hz, 2H). LC-MS *m*/*z* = 338.2–340.2 [M+H].

#### 2-Cyclopropyl-6-(7-fluoroimidazo[1,2-*a*]pyridin-3-yl)-8-methoxy-3,4-dihydroisoquinolin-1-one (**55a**)

Under inert atmosphere, 7-fluoroimidazo[1,2-*a*]pyridine **44** (30 mg, 0.22 mmol, 1.3 equiv), **53a** (50 mg, 0.17 mmol, 1 equiv), KOAc (33 mg, 0.34 mmol, 2 equiv), and Pd(dppf)Cl_2_·DCM adduct (7 mg, 0.008 mmol, 0.05 equiv) were suspended in dry DMAC (1.7 mL). The mixture was degassed with N_2_ and stirred at 120 °C for 2 h. The reaction mixture was concentrated, and the crude residue was purified by flash chromatography on silica gel (0 to 10% MeOH in DCM) to afford **55a** (55 mg, 93%). LC-MS *m*/*z* = 352.4 [M+H].

#### 6-(7-Fluoroimidazo[1,2-*a*]pyridin-3-yl)-8-methoxy-2-(2,2,2-trifluoroethyl)-3,4-dihydroisoquinolin-1-one (**55b**)

To a solution of **53b** (16.6 g, 49 mmol, 1 equiv) in dry and degassed DMAC (225 mL) were added 7-fluoroimidazo[1,2-*a*]pyridine **44** (7 g, 51.42 mmol, 1.05 equiv), KOAc (12 g, 122.5 mmol, 2 equiv), and Pd(dppf)Cl_2_·DCM (2.8 g, 3.43 mmol, 0.07 equiv). The reaction mixture was placed in a preheated bath at 125 °C and was stirred at this temperature for 3 h. The solvent was concentrated *in vacuo*, and the crude material was purified by flash chromatography on silica gel (eluting with heptane/EtOAc 7/3 to 0/1 then EtOAc/MeOH 100/0 to 95/5) to afford **55b** (14.9 g, 77%). ^1^H NMR (400 MHz, DMSO-*d*_6_) δ ppm 8.85–8.77 (m, 1H), 7.93 (s, 1H), 7.63–7.55 (m, 1H), 7.27 (d, *J* = 1.6 Hz, 1H), 7.21 (d, *J* = 1.5 Hz, 1H), 7.13–7.04 (m, 1H), 4.44–4.32 (m, 2H), 3.92 (s, 3H), 3.66 (t, *J* = 6.1 Hz, 2H), 3.02 (t, *J* = 6.1 Hz, 2H). LC-MS *m*/*z* = 394.2 [M+H].

#### 2-Cyclopropyl-8-methoxy-6-[7-(2-morpholinoethoxy)imidazo[1,2-*a*]pyridin-3-yl]-3,4-dihydroisoquinolin-1-one (**31**)

To a solution of 2-morpholinoethanol (95 μL, 0.78 mmol, 5 equiv) in dry DMF (2.1 mL) was added NaH (60% dispersion in mineral oil, 19 mg, 0.78 mmol, 5 equiv), and the mixture was stirred at room temperature for 5 min. Then, **55a** (55 mg, 0.16 mmol, 1 equiv) was added, and the reaction mixture was stirred at room temperature for 3 h. Then, 2-morpholinoethanol (95 μL, 0.78 mmol, 5 equiv) and NaH (60% dispersion in mineral oil, 19 mg, 0.78 mmol, 5 equiv) were added, and the reaction mixture was stirred at room temperature for 2 h. The reaction was quenched with a saturated aqueous NaHCO_3_ solution and extracted with EtOAc. The combined organic layers were dried over MgSO_4_, filtered, and concentrated. The crude material was purified by flash chromatography on silica gel (eluting with a gradient of 0 to 10% MeOH in DCM). The obtained residue was dissolved in DCM and washed with a 1N aqueous NaOH solution. The organic layer was passed through a phase separator and concentrated to afford **31** (34 mg, 47%). ^1^H NMR (400 MHz, methanol-*d*_4_) δ ppm 8.48 (dd, *J* = 7.6, 0.7 Hz, 1H), 7.64 (s, 1H), 7.16 (d, *J* = 1.6 Hz, 1H), 7.09 (d, *J* = 1.5 Hz, 1H), 6.97 (d, *J* = 2.5 Hz, 1H), 6.75 (dd, *J* = 7.6, 2.5 Hz, 1H), 4.26 (t, *J* = 5.4 Hz, 2H), 3.93 (s, 3H), 3.78–3.71 (m, 4H), 3.55 (t, *J* = 6.2 Hz, 2H), 2.98 (t, *J* = 6.3 Hz, 2H), 2.94–2.83 (m, 3H), 2.68–2.58 (m, 4H), 0.94–0.87 (m, 2H), 0.81–0.70 (m, 2H). LC-MS *m*/*z* = 463.7 [M+H].

#### 8-Methoxy-6-[7-(2-morpholinoethoxy)imidazo[1,2-*a*]pyridin-3-yl]-2-(2,2,2-trifluoroethyl)-3,4-dihydroisoquinolin-1-one (**32**)

To a solution of 2-morpholinoethanol (7.80 mL, 69.55 mmol, 5 equiv) in dry DMF (200 mL) at 0 °C was added NaH (60% dispersion in mineral oil, 2.00 g, 50.84 mmol, 4 equiv) portionwise, and the mixture was stirred at 0 °C for 10 min. At this temperature, **55b** (5.00 g, 12.71 mmol, 1 equiv) was added, and the reaction mixture was warmed up to room temperature and stirred for 3 h. The reaction mixture was cooled down to 0 °C and quenched with a saturated aqueous NaHCO_3_ solution and water. The mixture was extracted with EtOAc. The combined organic layers were washed with brine, dried over Na_2_SO_4_, filtered, and concentrated. The crude residue was purified by flash chromatography on silica gel (eluting with a gradient of 0 to 8% MeOH in DCM). The obtained material was dissolved in DCM and washed with a 2N aqueous NaOH solution twice. The phases were passed through a phase separator, and the filtrate was concentrated to afford **32** (5.77 g, 90%). ^1^H NMR (400 MHz, methanol-*d*_4_) δ ppm 8.51 (dd, *J* = 7.6, 0.7 Hz, 1H), 7.67 (s, 1H), 7.21 (d, *J* = 1.6 Hz, 1H), 7.13 (d, *J* = 1.5 Hz, 1H), 6.98 (d, *J* = 2.5 Hz, 1H), 6.76 (dd, *J* = 7.6, 2.5 Hz, 1H), 4.34 (q, *J* = 9.3 Hz, 2H), 4.26 (t, *J* = 5.4 Hz, 2H), 3.94 (s, 3H), 3.76–3.69 (m, 6H), 3.07 (t, *J* = 6.2 Hz, 2H), 2.88 (t, *J* = 5.4 Hz, 2H), 2.66–2.59 (m, 4H). LC-MS *m*/*z* = 505.4 [M+H].

### Methodology

#### ADP-Glo Kinase Assay with SIKs

1.11 to 2.23 nM of SIK1, 0.11 to 0.48 nM of SIK2, or 0.45 nM of SIK3 was incubated with 45 μM AMARA peptide and 5 μM ATP in 25 mM Tris pH 7.5, 0.5 mM EGTA, 0.01% Triton X-100, 5 mM MgCl_2_, and 2.5 mM DTT at rt for 120 min in the presence or absence of compound. To determine IC_50_ values, compounds were tested in a 10-point dose–response with 1/5 serial dilution starting from a top concentration of 20 μM. The kinase reaction was stopped after addition of an equal volume of ADP-Glo reagent and was incubated at rt for 40 min to remove all the remaining ATP. Afterward, a double volume of kinase detection reagent was added and incubated for a minimum of 30 min at rt before luminescence signal was measured with an Envision PerkinElmer plate reader.

#### SIK1, SIK2, SIK3 NanoBRET Assay

Briefly, 14 million HEK293 cells were transiently transfected with 70 μL JetPEI, 2.33 μg NanoLuc-SIK1 Fusion DNA (or NanoLuc-SIK2 or NanoLuc-SIK3), and 21 μg pBlueScript and reseeded in a T175 culture flask. One day later, the transfected HEK293 cells were harvested by trypsinization and resuspended in Opti-MEM without phenol red containing NanoBRET Kinase Tracer-04 (0.5 μM for SIK1 and SIK2, 1 μM for SIK3) and 30 μM Extracellullar NanoLuc inhibitor. Serial dilutions of test compounds and references (staurosporin) were prepared in PBS to obtain final highest concentrations of 30 μM in 0.3% DMSO. Cells were seeded on top of compound at 8,000 cells per 384 well. After 2 h incubation at 37 °C, 5% CO_2_, NanoBRET Nano-Glo substrate was added, and BRET readout was done with an Envision PerkinElmer plate reader within 10 min after addition of the substrate by recording donor (450 nm) and acceptor (610–630 nm) emissions. The ratio of the acceptor/donor emission was calculated and multiplied by 1,000 using the Envision software to obtain data in mBRET units.

#### CRTC3 Translocation Assay

Briefly, 1 million U2OS cells (ATCC, HTB-96) were thawed in a T175 culture flask in Dulbecco’s Modified Eagle’s Medium (DMEM) supplemented with 10% heat-inactivated fetal bovine serum (FBS) and 1% penicillin/streptomycin and incubated at 37 °C, 5% CO_2_. Three days after thawing, U2OS cells were reseeded in the same medium in the 384-well plate at a cell density of 2,500 cells per well and incubated for 16 to 20 h at 37 °C, 5% CO_2_. One day after seeding, serial dilutions of test compounds and references (forskolin) were prepared in DMEM and added on top of the cells to obtain final highest concentrations of 20 μM in 0.2% DMSO. After 1 h of compound incubation on the cells at 37 °C, 5% CO_2_, cells were fixed with 4% formaldehyde, followed by CRTC3 immunostaining using a rabbit monoclonal antibody to human CRTC3 and an Alexa Fluor 594-labeled goat anti-rabbit secondary antibody and by nuclei staining using Hoechst 33342. Stained cells were imaged on the IN Cell Analyzer 2200, and nuclear translocation was calculated using a specific Translocation Algorithm defined in the specific High-Content Imaging Software. At the same time, nuclei staining was analyzed.

#### *In Vitro* LPS-Triggered Human Primary Monocytes Assay

Activity of **32** was evaluated on LPS-stimulated cytokine release in monocytes. Peripheral blood mononuclear cells (PBMCs) were first isolated from blood using lymphoprep-based separation, a method which is based on the lower buoyant density of mononuclear cells (monocytes and lymphocytes) compared to other blood cell types such as erythrocytes and polymorphonuclear leukocytes (granulocytes). From these PBMCs, CD14+ monocytes were selected using antibody-coated magnetic beads (Miltenyi Biotec). CD14+ monocytes were seeded in 96-well plates and preincubated with a serial dilution of **32** for 1 h before LPS triggering (Sigma-Aldrich; 100 ng/mL final concentration). TNFα (4 h), IL-12 (20 h), and IL-10 (4 h) were measured in the supernatant at indicated time points after LPS triggering using standard enzyme-linked immunosorbent assay (ELISA)-based read-outs.

#### *In Vitro* LPS-Triggered Human Primary MdM Assay

To evaluate **32** on MdMs, CD14^+^ monocytes (isolated as described above) were differentiated toward macrophages using macrophage-colony stimulating factor (M-CSF [Immunotools]); 100 ng/mL final concentration) during 10 days. Differentiated MdMs were preincubated with a serial dilution of **32** for 1 h before LPS triggering (100 ng/mL final concentration). Supernatant was collected at 2 h for IL-10 and 20 h for TNFα after LPS triggering and measured using ELISA-based read-outs.

#### Human Whole Blood Activity

Blood was collected from healthy volunteers into lithium heparin tubes. 200 μL of blood was dispensed into a polypropylene 96-well microplate and preincubated in duplicate with DMSO 0.1% or **32** at different concentrations (from 30 to 0.003 μM, 3-fold dilutions to get 0.1% DMSO at the final) for 30 min at 37 °C. After this preincubation, blood was triggered with LPS (100 ng/mL) or vehicle (RPMI) for 2 h at 37 °C. Plates were centrifuged at 750*g* for 10 min at 4 °C, and around 100 μL of plasma was collected and frozen at −80 °C within 30 min. The quantification of TNFα was performed on plasma diluted 6 times using the human TNF-alpha Quantikine according to the manufacturer’s instructions. The optical density (OD) was determined at 450 nm on the Spectramax i3 or Ensight. The quantification of IL-10 was done without any dilution using the V-PLEX Human IL-10 Kit, and the electrochemiluminescence (ECL) was determined on the MESO QUICKPLEX SQ 120.

#### Mouse Pharmacokinetics

One group of six male CD1 mice was dosed intravenously with a dose level of 1 mg/kg, and one group of three male CD1 mice was dosed orally via a single gavage with a dose level of 5 mg/kg. The mice were fasted before the oral administration. For the iv route, compound was formulated as a solution in polyethylene glycol (PEG) 200 and water for injection (60/40; v/v). For the oral route, compound was formulated as a homogeneous suspension in Solutol/methyl cellulose (MC) 0.5% (2/98; v/v). Blood was sampled by retro-orbital puncture under light gaseous anesthesia into polypropylene tubes containing lithium heparin, and plasma was prepared. **32** was quantified in plasma using LC-MS/MS.

#### Rat Pharmacokinetics

One group of three male Sprague–Dawley rats was dosed intravenously with **32** at a dose level of 1 mg/kg, and one group of three male Sprague–Dawley rats was dosed orally via a single gavage with a dose level of 5 mg/kg. The rats were fasted before the oral administration. For the iv route, the compound was used as dihydrochloride salt formulated in saline NaCl 0.9%. For the oral route, the compound was formulated in Solutol/MC 0.5% (2/98; v/v). Blood was sampled under light gaseous anesthesia into polypropylene tubes containing lithium heparin, and an aliquot was used to prepare plasma. **32** was quantified in blood and in plasma using LC-MS/MS.

#### Dog Pharmacokinetics

One group of three male beagle dogs was dosed intravenously as a slow bolus with **32** at a dose level of 1 mg/kg, and one group of three male beagle dogs was dosed orally via a single gavage with a dose level of 5 mg/kg. The dogs were fasted before the intravenous and the oral administrations. For the iv route, the compound was used as dihydrochloride salt formulated in saline NaCl 0.9%. For the oral route, the compound was formulated in Solutol/MC 0.5% (2/98; v/v). Blood was sampled from the jugular vein into polypropylene tubes containing lithium heparin, and an aliquot was used to prepare plasma. **32** was quantified in blood and in plasma using LC-MS/MS.

#### *In Vivo* Mouse LPS Challenge

**32** was prepared in MC 0.5% the day before administration and gently mixed overnight at room temperature in the dark. The next day, **32** was administered orally to Balb/cN female mice at 1, 3, 5, 10, 30, and 60 mg/kg. Fifteen minutes later (corresponding to the *T*_max_ of the pharmacokinetic of **32**), 100 μg of LPS (in 0.2 mL of H_2_O) was injected intraperitoneally to mice. A control group was included with MC 0.5% po without LPS challenge. Mice were sacrificed 1.5 h after LPS challenge, and blood was collected by carotid exsanguination in EDTA tubes. Plasma samples were obtained by centrifugation for 15 min, 2,000*g* at +4 °C and frozen at −80 °C before cytokine quantifications and bioanalysis. IL-10 and TNFα were quantified by alphaLISA according to the manufacturer’s instructions. Optical densities were determined using Ensight (PerkinElmer). Statistical analysis was performed on raw data or log transformed data (a group with another compound was removed from the graph and for statistical analysis). The normality of residuals and equality of variances for a parametric analysis were checked. Statistical analysis of plasma TNFα levels was performed with a Kruskal–Wallis and Dunn’s post-test: *p < 0.05; ***p < 0.001. Statistical significance of plasma IL-10 levels was calculated using ANOVA and Dunnett’s multiple comparison test: ***p < 0.001. Statistics were done versus LPS + vehicle group (***: *p* < 0.001; **: *p* < 0.01; *: *p* < 0.05).

#### Dextran Sodium Sulfate (DSS)-Induced Colitis Model

Balb/cJ female mice (Janvier, Le Genest-Sant-Isle, France) were housed in a dedicated in-house animal facility under specific pathogen-free conditions according to the Federation for Laboratory Animal Science Association guidelines. The study as performed according to ethical guidelines approved by the animal Institutional Care and Use Committee of Galapagos controlled by the French Authorities (Agreement No. 93-063-06, DDPP, Seine Sant Denis). Animals were housed in filter top cages, provided with filtered tap water and standard chow ad libitum, and maintained at 22 ± 2 °C in 55 ± 10% humidity on a 12 h light/dark cycle. The randomization of mice in the different groups was based on BW at study start. A model of chronic colitis was induced in 6- to 8-week-old mice with two DSS cycles, consisting of 4% DSS administered in drinking water for 4 days followed by 3 days of regular drinking water followed by 4 days of DSS 4%. The mice (*n* = 10 per group) were orally administered vehicle (methyl cellulose 0.5%) or 3, 10, or 30 mg/kg of **32** b.i.d. from day 1 of DSS administration and throughout the dosing period. Steady-state PK was performed on day 9. For each group treated with **32**, each mouse was sampled once at one of the selected time points: 0 h (*n* = 3), 0.25 h (*n* = 3), 1.5 h (*n* = 2), 6 h (*n* = 3); then the average plasma concentration at each time point was used to perform PK analysis. Clinical parameters were measured every other day. DAI (a combined score of BW loss, stool consistency, and fecal blood loss) was recorded daily (except on weekends). Each criterion was graded from 0 to 4. Animals were sacrificed by cervical dislocation after isoflurane anesthesia at day 12 of the experiment. For histological analysis, colon tissues were harvested, fixed in buffered formalin, and embedded in paraffin. Sections of 4 μm were cut and stained with PAS using a standard procedure. To evaluate colon lesion by histology, MCHI scoring was performed. Eight histological components were assessed: inflammatory infiltrate, goblet cell loss, crypt hyperplasia, crypt density, muscle thickness, submucosal infiltration, ulcerations, and crypt abscesses (all categorized from 0 to 3). A total MCHI score was obtained by summing up the scores from the eight different components.^35^ Statistical analysis was done with a one-way ANOVA performed on the Log(Y) AUC of DAI data transformation without intact vehicle group. Dunnett’s post hoc multiplicity correction procedure was applied to the AUC of DAI data. For MCHI, statistical analysis was performed using a one-way ANOVA without intact vehicle group followed by a Dunnett’s posthoc multiplicity correction procedure.

#### CYP Inhibition Assay

A 5 mM stock solution of **32** was prepared in methanol. This stock was further serially diluted 1:3 in methanol and then added to a mixture containing 50 mM potassium phosphate buffer pH 7.4, human liver microsomes, and probe substrate. After prewarming for 5 min at 37 °C, the reaction was started by adding cofactor mix (7.65 mg/mL glucose-6-phosphate, 1.7 mg/mL NADP, 6 U/mL of glucose-6-phosphate dehydrogenase), resulting in seven final concentrations of **32** in the range 0.137–100 μM (2% methanol). Final concentrations of cofactor mix components were as follows: 1.56 mg/mL glucose-6-phosphate, 0.34 mg/mL NADP, 1.2 U/mL of glucose-6-phosphate dehydrogenase. After incubation at 37 °C for 5–15 min, the reaction (aliquot of 50 μL) was terminated with 150 μL acetonitrile/methanol (2/1) solution with internal standard (warfarin for 2C9, diclofenac for all other tested isoforms). Samples were centrifuged and the supernatant fractions analyzed by LC-MS/MS. The instrument responses (ratio of probe substrate and internal standard peak areas) were referenced to those for solvent controls (assumed as 100%) in order to determine the percentage reduction in probe metabolism.

#### CYP Time-Dependent Inhibition

A 5 mM stock solution of compound to be assessed was prepared in methanol. This stock was serially diluted 1:3 in methanol and then added in duplicates to mixture containing 50 mM potassium phosphate buffer pH 7.4 and human liver microsomes. A total of 7 different concentrations (0.14–100 μM in final reaction mixture; 2% methanol) of compound were prepared in duplicates. After prewarming for 5 min at 37 °C, the 20 min preincubation was started by adding a probe substrate (midazolam or testosterone) to the first concentration range (first replica) and a cofactor mix (7.65 mg/mL glucose-6-phosphate, 1.7 mg/mL NADP, 6 U/mL of glucose-6-phosphate dehydrogenase) to the second replica. Final concentrations of cofactor mix components were as follows: 1.56 mg/mL glucose-6-phosphate, 0.34 mg/mL NADP, 1.2 U/mL of glucose-6-phosphate dehydrogenase. After preincubation completion, the reaction was finally started by adding the cofactor mix to the first replica and probe substrate to second replica (opposite to preincubation step). After incubation at 37 °C for 5–15 min, the reaction (aliquot of 50 μL) was terminated with 150 μL acetonitrile/methanol (2/1) solution with internal standard (diclofenac). Samples were centrifuged and the supernatant fractions analyzed by LC-MS/MS.

The instrument responses (ratio of probe substrate and internal standard peak areas) were referenced to those for solvent controls (assumed as 100%) in order to determine the percentage reduction in probe metabolism.

#### CYP Induction

Cryopreserved human hepatocytes from a single donor are seeded on a 96-well collagen coated plate so that the final seeding density is 0.1 × 106 cells/well (final volume per well 0.1 mL). The cells are then incubated in seeding medium at 37 °C, 95% humidity, 5% CO_2_ to allow the cells to attach. After 4 h, the seeding medium is replaced with 0.1 mL of prewarmed serum-free Williams E medium (William’s E containing 100 IU/mL penicillin, 100 μg/mL streptomycin, 10 μg/mL insulin, 2 mM l-glutamine, and 0.1 μM hydrocortisone). The next day, cells are dosed with test compound in assay medium (final test compound concentration 10 μM; final DMSO concentration 0.1%). Positive control inducer, rifampicin for CYP3A4, is incubated alongside the test compound. Negative control wells are included where the test compound is replaced by vehicle solvent (typically 0.1% DMSO in assay medium). Each test or control compound is dosed in triplicate at a single concentration. The cells are exposed to the solutions for 72 h with fresh solution added every 24 h. For mRNA assessment, all media is removed from each of the wells, and the cells were washed once with 0.1 mL of prewarmed assay medium. The cells are lysed by adding 100 μL of lysis buffer to each well. Total RNA is then isolated from the hepatocyte lysates. Reverse transcription is performed, and quantitative PCR analysis is performed on the resulting cDNA, using gene-specific primer probe sets for CYP3A4 target cDNA and endogenous control. Samples are analyzed using an ABI 7900 HT real-time PCR system. For mRNA assessment, relative fold mRNA expression is determined based on the threshold cycle (CT) data of target gene relative to endogenous control for each reaction and normalized to negative control using the 2-ΔΔCT. Data are expressed as fold activation relative to the vehicle control and, as a percent, to the 10 μM rifampicin using the following formula:
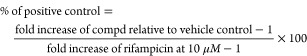


#### hERG Channel Test (Manual Patch Clamp Assay)

The effects of **32** at the nominal concentrations of 0.1, 1, 10, and 100 μM on the delayed rectifier potassium current (IKr) encoded by hERG were studied at a stimulation frequency of 6 pulses/min (0.1 Hz) in 6 different stably transfected HEK293 cells. A vehicle group (four superfusion periods with 0.3% DMSO in extracellular solution) was included in the study for comparison, and terfenadine (nominal concentration of 1 μM) was used as reference substance.

#### Mutagenicity Test

Compound **32** was assayed for mutation in five histidine-requiring strains (TA98, TA100, TA1535, TA1537, and TA102) of *Salmonella typhimurium*, both in the absence and in the presence of metabolic activation by an Aroclor 1254-induced rat liver postmitochondrial fraction (S9), in a single experiment. Treatments of all tested strains were performed using final concentrations of **32** of 5, 16, 50, 160, 500, 1,600, and 5,000 μg/plate, plus vehicle and positive controls.

Without metabolic activation: the following procedure was followed for each strain used in the test. Each bacterial suspension was from a culture that was agitated overnight at ca. 37 °C. Five to 20 μL of the test item at the relevant concentrations, 0.1 mL of the bacterial suspension, and 95 μL of PBS were successively added. The plates were incubated at ca. 37 °C under stirring for 90 min. Then, the content of each well was transferred in 2 mL of top agar, supplemented with 10% of 0.5 mM biotin histidine solution, maintained in a state of superfusion at ca. 45 °C. The contents of each tube were agitated, and then spread out in Petri plates containing 20 mL of minimum agar. For each strain and incubation condition, each dose was tested in three plates: after incubation at ca. 37 °C for about 68 h, the number of revertant colonies was determined for each plate.

With metabolic activation: The method was the same as described above, except that the PBS was replaced by 95 μL of the S9-mix metabolic activation system.

#### Modeling

All calculations were carried out using Schrödinger software suite, release 2017–3.^36^

#### Ligand Docking

All docked compounds were built and protonated using LigPrep software,^37^ whereas ionization states at pH 7 ± 2 were calculated with Epik.^38,39^

For the internal SIK3 X-ray structure, hydrogen atoms were added to the protein through the Protein Preparation Wizard tool.^40^ In order to optimize the H-bond network, the most probable protonation state of the residues was carefully selected by visual inspection, and hydrogen atoms were minimized using OPLS3 force field.^41^ Before running the docking procedure, all water molecules present in the structure were removed.

Docking of the ligands was carried out with Glide.^42−44^ A docking grid was generated using SIK3 prepared structure. The cocrystallized ligand was selected as the center of the grid, a H-bond constraint with the hinge H-bond donor (Ala145 NH for SIK3) was created, whereas the rest of the settings were kept as default. For the docking run, the flexible docking standard precision (SP) option was selected, together with an enhanced sampling protocol (four times) of the ligands. The constraint was applied to all docking runs, whereas the number of poses to return was set as three for each ligand.

The binding modes were then selected based on spatial geometries of the ligand within the binding cavity, complementarity with the pocket (shape and electrostatic complementarity), H-bond geometries of the protein–ligand interactions, and docking score.

#### Homology Models

Homology models for SIK1 and SIK2 were built using the kinase domain of the internal X-ray structure of SIK3 as template (residues 59 to 339) using Prime^45^ as part of the Schrödinger suite. The native ligand was kept in the active site, and only the side chains of non-matching residues were rebuilt. A single knowledge-based model was requested. The resulting structure was further refined using Prime’s Refine Protein–Ligand Complex tool: the residues within 5 Å of the ligand and the ligand were submitted to an implicit-solvent minimization using a local optimization sampling algorithm. The variable-dielectric generalized Born model (VSGB) for water was selected together with a dielectric constant of 80. The OPLS3 force field was used.

#### Molecular Dynamics Simulations

Molecular dynamics simulations were run using the Desmond^46^ package included in the Schrödinger suite. The UBA domain of the internal SIK3 X-ray structure was removed to keep only the kinase domain (residues 59 to 339). The protein structures obtained by homology modeling were used for SIK1 and SIK2. Ligand geometries were obtained from the docking studies and were parametrized using OPLS3 force field. The complexes generated were initially solvated in a cubic box with SPC water molecules leaving at least 10 Å between the solute atoms and the border of the box. The systems were then neutralized with 7 Cl^–^ counterions (8 in the case of positively charged ligands), and NaCl salt concentration of 0.15 M was added. The systems generated were equilibrated and gradually heated from 0 to 300 K using Desmond default equilibration protocol. After the equilibration, the systems were subjected to 50 ns MD simulations in the NPT ensemble at 1.01325 bar (by Martyna–Tobias–Klein barostat) and 300 K (by Nosé–Hoover chain thermostat), setting a cutoff of 9 Å for the short-range non-bonded interactions. Trajectories were saved every 100 ps, and for each system two replicas, with different starting velocities (random), were run. Analysis of the simulation was based on protein and ligands RMSD values, H-bond occupancy along 100 ns simulation, and visual inspection of the binding geometries.

## Supporting Information Available

Table with individual percentage of kinase inhibition by **32** at 1 μM, additional selectivity data, and NMR spectra and HPLC traces of synthesized compounds (PDF)
Molecular formula strings with associated data (CSV)

## Abbreviations Used

AMPK: adenosine monophosphate-activated protein kinase
BMDC: bone marrow-derived dendritic cell
BMDM: bone marrow-derived macrophage
BMMC: bone marrow-derived mast cell
BW: body weight
CV: coefficient of variation
CREB: cAMP-response element binding protein
CRTC: CREB-regulated transcriptional coactivator
DAI: disease activity index
dd: doublet of doublets
ddd: doublet of doublet of doublets
DMAC: *N,N*-dimethylacetamide
dq: doublet of quartets
DSS: dextran sodium sulfate
dt: doublet of triplets
dtd: doublet of triplet of doublets
dioxane: 1,4-dioxane
DIPEA: *N,N*-diisopropylethylamine
DDI: drug–drug interaction
Et_2_O: diethyl ether
EtOAc: ethyl acetate
EtOH: ethanol
FLDM: fetal liver-derived macrophage
HATU: *O*-(7-azabenzotriazol-1-yl)-*N,N,N′,N′*-tetramethyluronium hexafluorophosphate
HDAC: histone deacetylase
IBD: inflammatory bowel disease
IL: interleukin
LKB1: liver kinase B1
LPS: lipopolysaccharide
MC: methyl cellulose
MCHI: mouse colitis histology index
MdM: monocyte-derived macrophage
MeCN: acetonitrile
MeOH: methanol
PAS: periodic acid–Schiff
PBMC: peripheral blood mononuclear cell
ND: not determined
Pd(PPh_3_)_4_: tetrakis(triphenylphosphine)palladium(0)
Pd(dppf)Cl_2_·DCM: [1,1′-bis(diphenylphosphino)ferrocene]dichloropalladium(II) (1:1)
PEG: polyethylene glycol
PKA: protein kinase A
RuPhos: dicyclohexyl(2′,6′-diisopropoxy-[1,1′-biphenyl]-2-yl)phosphine
SEM: standard error of the mean
SIK: salt-inducible kinase
SNAr: nucleophilic aromatic substitution
tBuXPhos: 2-di-*tert*-butylphosphino-2′,4′,6′-triisopropylbiphenyl
TDI: time-dependent inhibition
TNFα: tumor necrosis factor alpha
Xantphos: 4,5-bis(diphenylphosphino)-9,9-dimethylxanthene
