# Supplementary material for: Discovery of Clinical Candidate GLPG3970: A Potent and Selective Dual SIK2/SIK3 Inhibitor for the Treatment of Autoimmune and Inflammatory Diseases
Source: J Med Chem. 2024 Mar 29;67(7):5233–58. doi: 10.1021/acs.jmedchem.3c02246 (PMC11017251; doi:10.1021/acs.jmedchem.3c02246)
Supplement: Supplementary file 1 — jm3c02246_si_001.pdf [file jm3c02246_si_001.pdf]

Supporting Information

Discovery of Clinical Candidate GLPG3970: A Potent and Selective Dual SIK2/SIK3 Inhibitor for the Treatment of Autoimmune and Inflammatory Diseases

Christophe Peixoto,[1]* Agnes Joncour,[1] Taouès Temal-Laib,[1] Amynata Tirera,[1] Aurélie Dos Santos,[1] Hélène Jary,[1] Denis Bucher,[1] Wendy Laenen,[2] Anna Pereira Fernandes,[2] Stephanie Lavazais,[1] Carole Delachaume,[1] Didier Merciris,[1] Corinne Saccomani,[1] Michael Drennan,[2] Miriam López-Ramos,[1] Emanuelle Wakselman,[1] Sonia Dupont,[1] Monica Borgonovi,[1] Carlos Roca Magadan,[1] Alain Monjardet,[1] Reginald Brys,[2] Steve De Vos,[2] Martin Andrews,[2] Juan-Miguel Jimenez,[2] David Amantini,[1] Nicolas Desroy[1]*

*Corresponding authors

Christophe Peixoto – NovAlix, 102 Avenue Gaston Roussel, 93230 Romainville, France Email: cpeixoto@novalix.com; ORCID: 0009-0009-0421-9361

Nicolas Desroy – Galapagos SASU, 102 Avenue Gaston Roussel, 93230 Romainville, France; Email: Nicolas.Desroy@glpg.com; ORCID: 0000-0002-4879-3439

[1]Galapagos SASU, 93230 Romainville, France

[2]Galapagos NV, 2800 Mechelen, Belgium

Contents

Table S1. .... S2

Additional Selectivity Data for Compound 32. .... S10

LCMS Traces. S11

NMR Traces. S54

Table S1. Percentage of kinase inhibition by **32** at 1 µM (372 kinases), assays performed at Eurofins

Kinase Name	Percentage of Inhibition
AAK1	16
ABL1	58
ABL2	29
ACVR1	39
ACVR1B	23
ACVR2A	-24
ACVRL1	5
AKT1	10
AKT2	-5
AKT3	0
ALK	1
ARAF	22
ATM	-11
AURKA	23
AURKB	20
AURKC	4
AXL	7
BLK	46
BMP2K	7
BMPR1B	26
BMPR2	25
BMX	-5
BRAF	3
BRSK1	-6
BRSK2	20
BTK	12
CAMK1	8
CAMK1D	22
CAMK1G	9
CAMK2A	13
CAMK2B	-7
CAMK2D	-1
CAMK2G	10
CAMK4	-5
CAMKK1	-4
CAMKK2	21
CDC42BPA	-6
CDC42BPB	-5
CDC42BPG	6
Cdc7/cyclinB1	16
CDK1/CCNB1	-6
CDK12/CCNK	16

CDK13/CCNK	26
CDK14/CCNY	-6
CDK16/CCNY	-13
CDK17/CCNY	-6
CDK18/CCNY	17
CDK2/CCNA2	9
CDK2/CCNE1	20
CDK3/CCNE1	7
CDK4/CCND3	2
CDK5/p25CDK5R1	-5
CDK5/p35CDK5R1	-20
CDK6/CCND3	1
CDK7/CCNH/MNAT1	9
CDK9/CCNT1	8
CDKL1	-18
CDKL2	23
CDKL3	0
CDKL4	2
CHEK1	0
CHEK2	0
CHUK	-2
CIT	4
CLK1	12
CLK2	9
CLK3	4
CLK4	21
CSF1R (FMS)	31
CSK	-3
CSNK1A1	-13
CSNK1D	-4
CSNK1E	-9
CSNK1G1	-5
CSNK1G2	18
CSNK1G3	14
CSNK2A1	15
CSNK2A1/B	3
CSNK2A2	1
DAPK1	33
DAPK2	-6
DAPK3	5
DCLK1	-6
DCLK2	-6
DCLK3	11
DDR1	37
DDR2	-6
DMPK	-1

DYRK1A	-11
DYRK1B	14
DYRK2	10
DYRK3	6
EEF2K	7
EGFR	5
EIF2AK1	8
EIF2AK2	8
EIF2AK3	24
EIF2AK4	-13
EPHA1	6
EPHA2	-13
EPHA3	30
EPHA4	30
EPHA5	13
EPHA7	9
EPHA8	-33
EPHB1	12
EPHB2	15
EPHB3	8
EPHB4	-3
ERBB2	-1
ERBB4	-3
ERN1	-17
FER	2
FES	-3
FGFR1	7
FGFR2	25
FGFR3	-3
FGFR4	4
FGR	19
FLT1	11
FLT3	-27
FLT4	9
FRK	-2
FYN	23
GRK1	6
GRK2	12
GRK3	-8
GRK5	-6
GRK6	13
GRK7	-6
GSK3A	18
GSK3B	-30
HASPIN	-7
HCK	2

HCK act	16
HIPK1	-11
HIPK2	10
HIPK3	13
HIPK4	-10
ICK	-5
IGF1R	0
IGF1R act	-14
IKBKB	1
IKBKE	16
INSR	7
INSR act	9
INSRR	9
IRAK1	-4
IRAK4	8
ITK	-7
JAK1	28
JAK2	-10
JAK3	11
KDR	8
KIT	12
LATS1	18
LATS2	-3
LCK	17
LCK activated	10
LIMK1	36
LIMK2	-15
LRRK2	9
LTK	-1
LYN	48
MAK	19
MAP2K1	-9
MAP2K2	-4
MAP2K3	13
MAP2K6	11
MAP3K10	-1
MAP3K11	15
MAP3K2	21
MAP3K20	41
MAP3K21	-12
MAP3K3	-30
MAP3K5	-6
MAP3K7	-13
MAP3K9	12
MAP4K1	13
MAP4K2	-11

MAP4K3	3
MAP4K4	17
MAP4K5	17
MAPK1	7
MAPK10	11
MAPK11	1
MAPK12	-16
MAPK13	-13
MAPK14	-28
MAPK3	2
MAPK8	14
MAPK9	22
MAPKAPK2	-17
MAPKAPK3	19
MAPKAPK5	-2
MARK1	0
MARK2	1
MARK3	0
MARK4	-1
MELK	3
MERTK	20
MET	4
MINK1	-1
MKNK2	54
MOK	18
MST1R	2
MTOR	12
MTOR/FKBP1A	-14
MUSK	-1
MYLK	-4
MYLK2	-2
MYO3B	-12
NEK1	24
NEK11	7
NEK2	-6
NEK3	-15
NEK4	12
NEK6	4
NEK7	12
NEK9	-8
NIM1K	5
NLK	17
NTRK1	17
NTRK2	-3
NTRK3	3
NUAK1	0

NUAK2	7
OXSR1	23
PAK1	-2
PAK2	-4
PAK3	1
PAK4	14
PAK5	-1
PAK6	13
PASK	-8
PDGFRA	20
PDGFRB	11
PDK2	13
PDK4	-4
PDPK1	3
PHKG1	1
PHKG2	-14
PIK3C2A	2
PIK3C2G	-8
PIK3CA/PIK3R1(p110a/p85a)	-6
PIK3CB	3
PIK3CD	9
PIK3CG	0
PIM1	12
PIM2	21
PIM3	16
PIP4K2A	-5
PIP5K1A	1
PIP5K1C	-10
PKN1	-4
PKN2	-11
PLK1	-11
PLK2	-13
PLK3	-3
PLK4	18
PNCK	4
PRKAA1	30
PRKAA2	15
PRKACA	12
PRKACB	2
PRKCA	10
PRKCB	0
PRKCB2	8
PRKCD	-17
PRKCE	7
PRKCG	-3
PRKCH	4
PRKCI	-7
PRKCQ	-19

PRKCZ	-16
PRKD1	-10
PRKD2	-9
PRKD3	-14
PRKDC	-29
PRKG1	2
PRKG1b	3
PRKG2	12
PRKX	-4
PRPF4B	16
PTK2	-6
PTK2B	-8
PTK6	-6
RAF1	7
RET	-2
RIPK1	12
RIPK2	79
ROCK1	5
ROCK2	28
ROS1	-9
RPS6KA1	3
RPS6KA2	-5
RPS6KA3	0
RPS6KA4	0
RPS6KA5	-2
RPS6KA6	-40
RPS6KB1	8
SBK1	-1
SGK1	6
SGK2	17
SGKL	14
SIK1	33
SIK2	98
SIK3	93
SLK	0
SNRK	20
SRC	15
SRMS	25
SRPK1	-9
SRPK2	-14
SRPK3	18
STK10	-3
STK11	-3
STK16	20
STK17A	31
STK17B	9
STK24	5

STK25	16
STK26	9
STK3	-5
STK32A	-11
STK32B	-11
STK32C	14
STK33	3
STK35	19
STK38	10
STK38L	6
STK39	-17
STK4	-10
SYK	3
TAF1L	-15
TAOK1	2
TAOK2	-5
TAOK3	-13
TBK1	8
TEC	30
TEK	5
TGFBR1	11
TGFBR2	46
TLK1	-9
TLK2	11
TNIK	30
TNK2	-2
TRIB2	4
TRPM7	-13
TSSK1B	-9
TSSK2	3
TSSK3	13
TSSK4	7
TTBK1	8
TTBK2	-7
TTK	21
TXK	-14
TYK2	23
TYRO3	-3
ULK1	-1
ULK2	9
ULK3	15
VRK1	18
VRK2	-4
WEE1	15
WEE2	13
WNK1	17

WNK2	7
WNK3	-1
WNK4	12
YES1	38
ZAP70	-42

Additional Selectivity Data for Compound 32.

**Kinase, Assay**	**Provider**	**Result**
MLKL (full-length) KINOMEscan^{TM}	Eurofins	Kd > 10,000 nM
RIPK3 enzymatic activity	Reaction Biology	IC_{50} > 10,000 nM

LCMS Traces.

Analysis performed on Acquity UPLC systems from Waters, controlled by Masslynx software. Compound **8** and Compound **9** have been previously described by Temal-Laib et al.[1]

Compound **10**

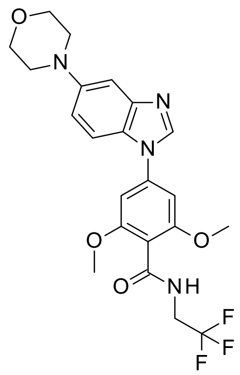

Exact Mass: 464.17
Molecular Formula: $C_{22}H_{23}F_3N_4O_4$

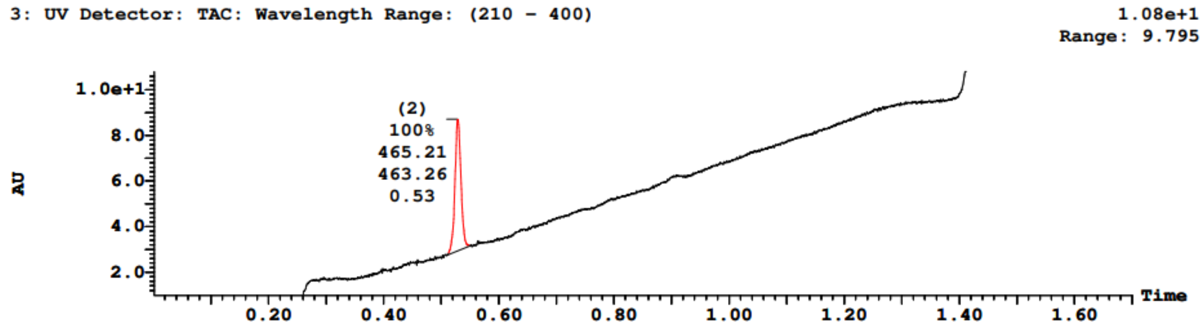

Peak Number	Compound	Time	AreaAbs	Area %Total	Width	Height	Mass Found	BPM
2		0.53	7e+004	100.00	0	6e+006	Not Found	465, 463

Peak ID	Compound	Time	Mass Found
2		0.53	Not Found

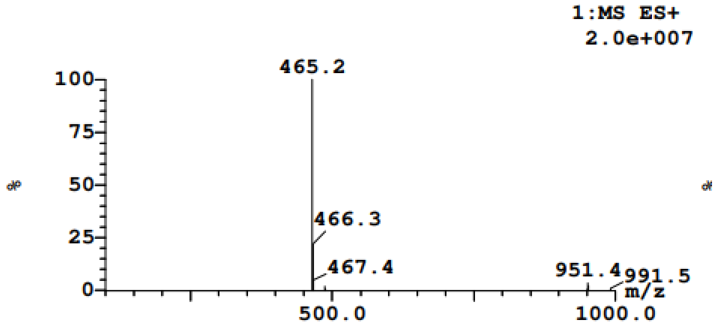

Compound **11**

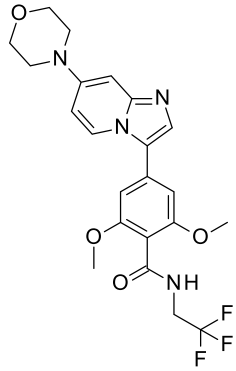

Exact Mass: 464.17
Molecular Formula: $C_{22}H_{23}F_3N_4O_4$

3: UV Detector: TAC: Wavelength Range: (210 - 400)   7.834e+1
Range: 8.724e+1

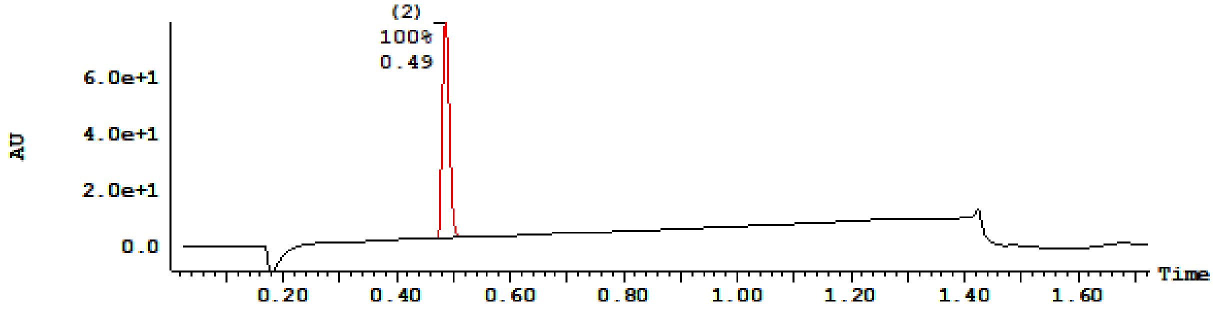

Peak Number	Compound	Time	AreaAbs	Area %Total	Width	Height	Mass Found
2		0.49	1e+006	100.00	0	8e+007	Not Found

Peak ID	Compound	Time	Mass Found
2		0.49	Not Found

2: (Time: 0.49) Combine (117:127-(102:113+130:142))   1:MS ES+
6.0e+007

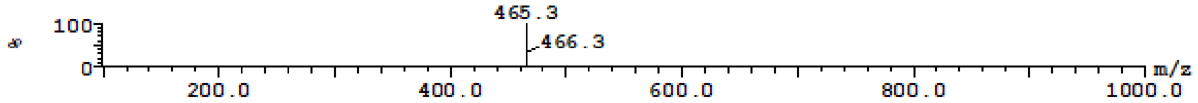

Compound **12**

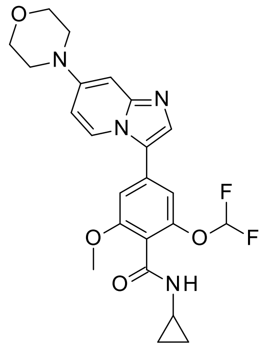

Exact Mass: 458.18
Molecular Formula: $C_{23}H_{24}F_2N_4O_4$

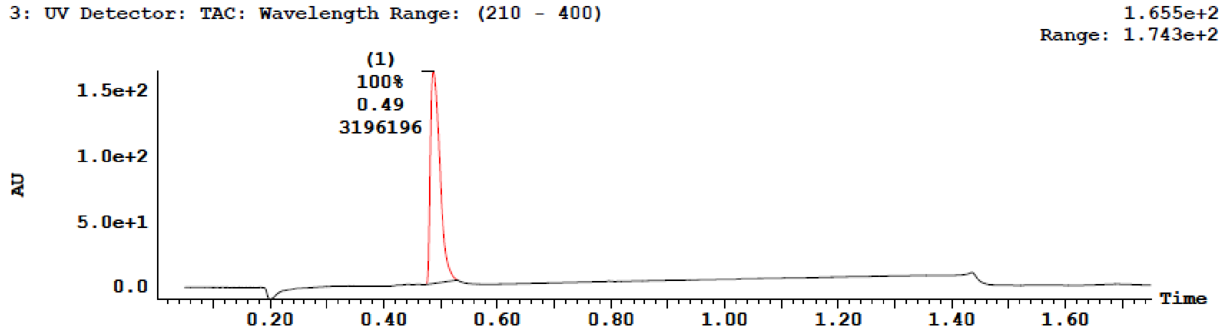

Peak Number	Compound	Time	AreaAbs	Area %Total	Width	Height	Mass Found
1		0.49	3e+006	100.00	0	2e+008	Not Found

Peak ID	Compound	Time	Mass Found
1		0.48	Not Found

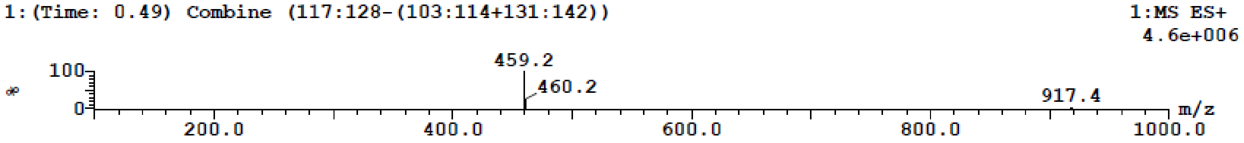

Compound **13**

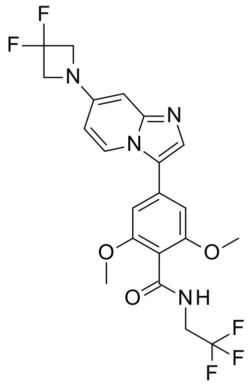

Exact Mass: 470.14
Molecular Formula: $C_{21}H_{19}F_5N_4O_3$

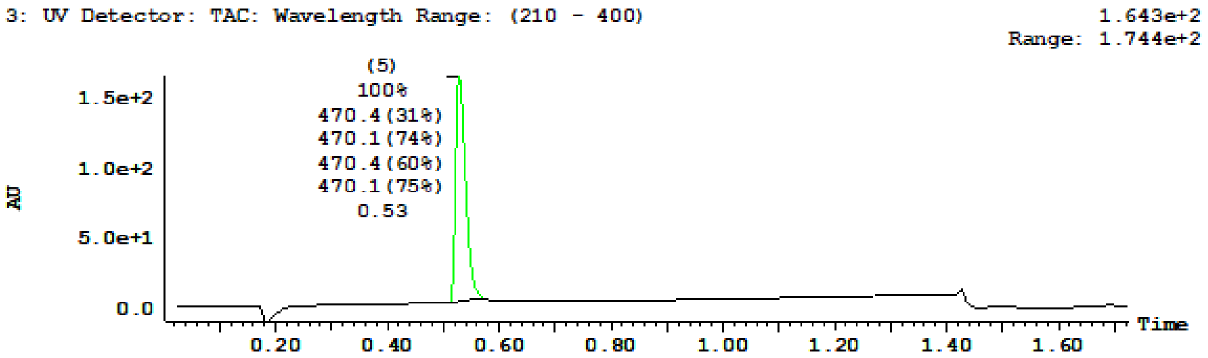

Peak Number	Compound	Time	AreaAbs	Area %Total	Width	Height	Mass Found
5	Found	0.53	3e+006	100.00	0	2e+008	l377, 470.3926

Peak ID	Compound	Time	Mass Found
5	Found	0.53	471,471

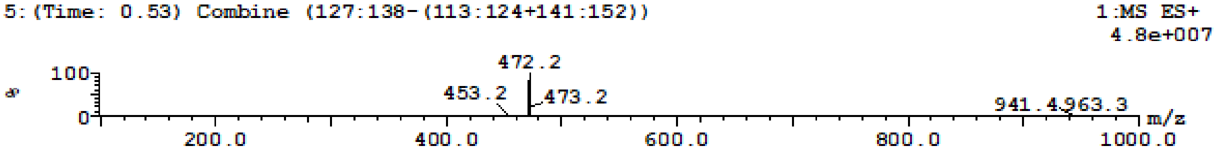

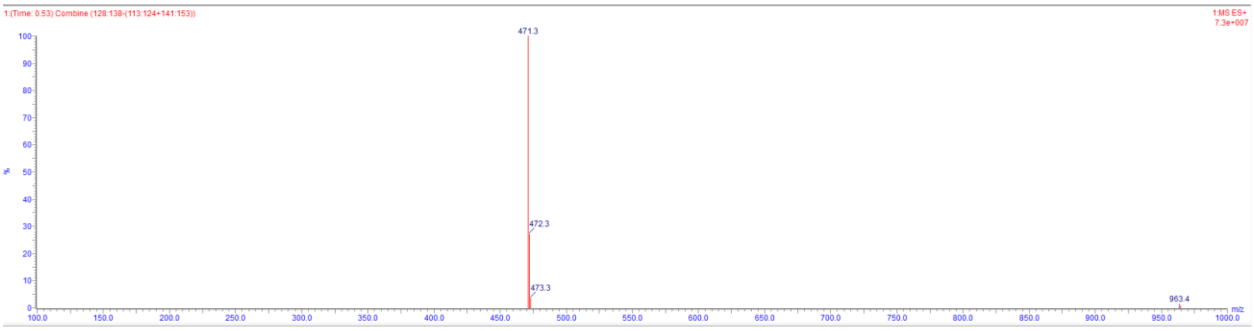

Compound **14**

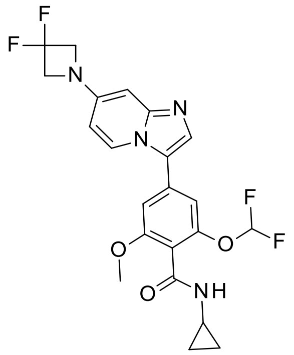

Exact Mass: 464.15
Molecular Formula: $C_{22}H_{20}F_4N_4O_3$

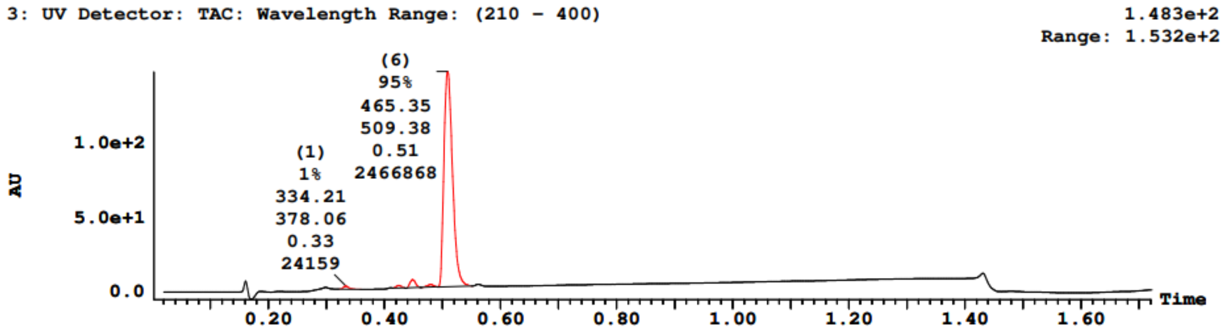

Peak Number	Compound	Time	AreaAbs	Area %Total	Height	BPM
1		0.33	2e+004	0.93	2e+006	334, 378
2		0.43	2e+004	0.64	2e+006	374, 347
4		0.45	6e+004	2.25	5e+006	425, 469
5		0.48	2e+004	0.84	2e+006	467, 511
6		0.51	2e+006	95.34	1e+008	465, 509

Sample Report (continued):

Peak ID	Mass Found	Time
6	Not Found	0.51

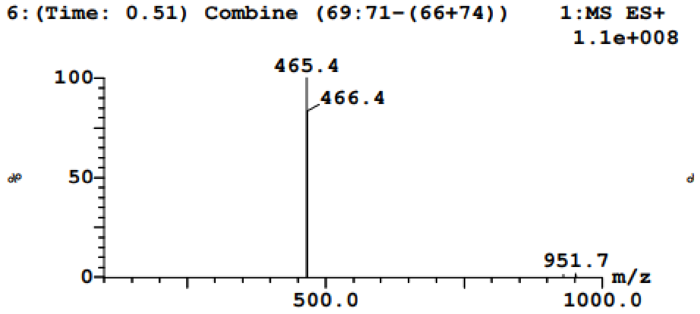

Compound **15**

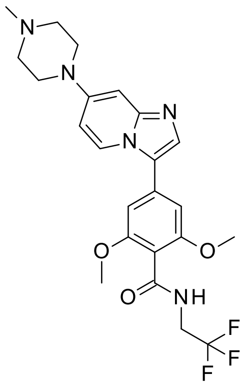

Exact Mass: 477.2
Molecular Formula: $C_{23}H_{26}F_3N_5O_3$

3: UV Detector: TAC: Wavelength Range: (210 - 400)

1.345e+1
Range: 2.181e+1

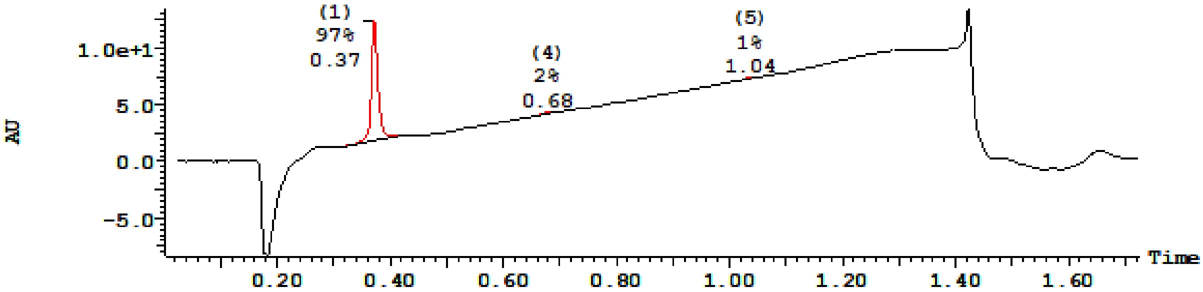

Peak Number	Compound	Time	AreaAbs	Area %Total	Width	Height	Mass Found
1		0.37	1e+005	97.15	0	1e+007	Not Found
4		0.68	3e+003	1.75	0	2e+005	Not Found
5		1.04	2e+003	1.11	0	1e+005	Not Found

Peak ID	Compound	Time	Mass Found
1		0.38	Not Found

1: (Time: 0.37) Combine (88:98-(73:85+102:113))

1:MS ES+
1.1e+007

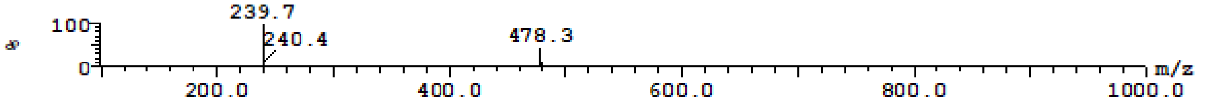

Compound **16**

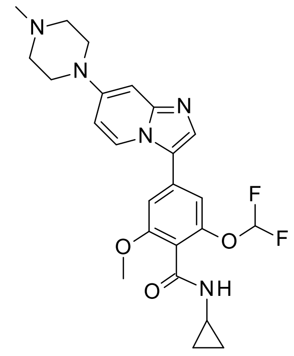

Exact Mass: 471.21
Molecular Formula: $C_{24}H_{27}F_2N_5O_3$

3: UV Detector: TAC: Wavelength Range: (210 - 400)   3.262e+1
Range: 4.177e+1

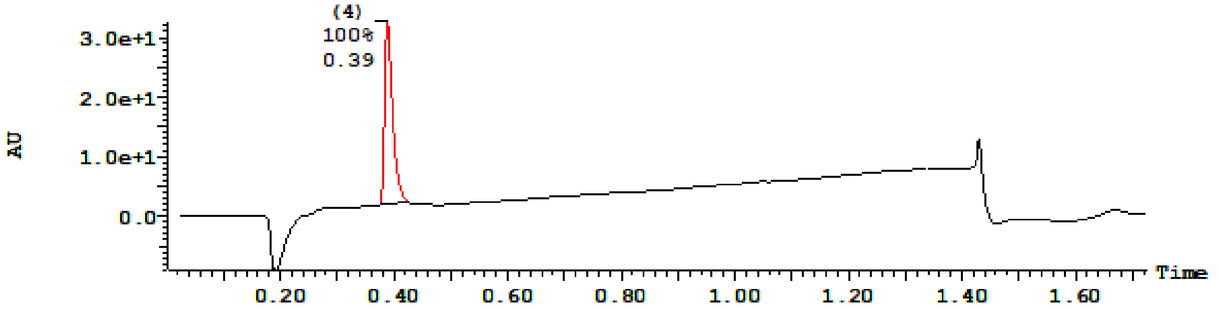

Peak Number	Compound	Time	AreaAbs	Area %Total	Width	Height	Mass Found
4		0.39	5e+005	100.00	0	3e+007	Not Found

Peak ID	Compound	Time	Mass Found
4		0.40	Not Found

4: (Time: 0.39) Combine (92:102-(77:88+105:117))   1:MS ES+
2.0e+007

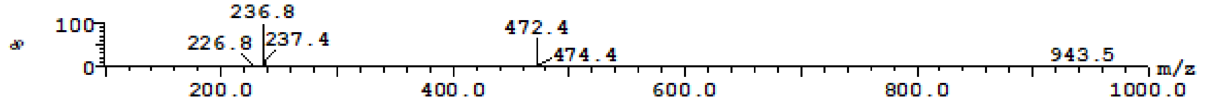

Compound **17**

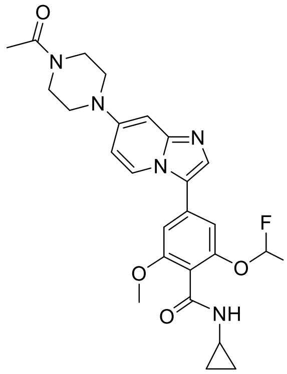

Exact Mass: 499.2

Molecular Formula: $C_{25}H_{27}F_2N_5O_4$

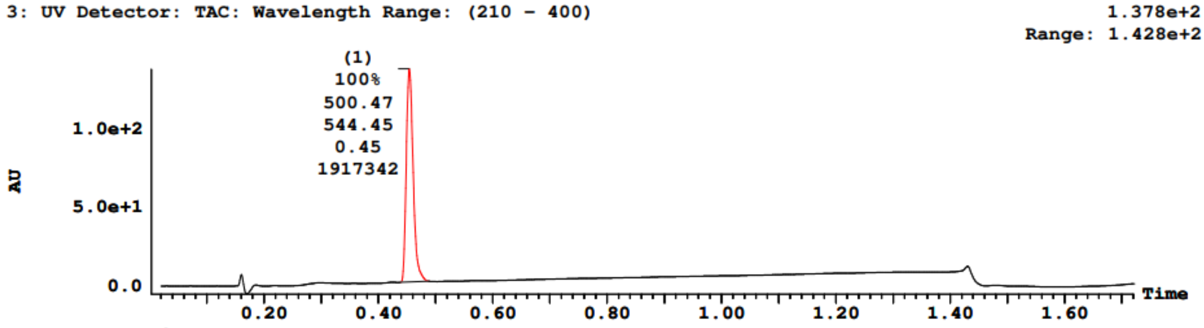

Peak Number	Compound	Time	AreaAbs	Area %Total	Height	BPM
1		0.45	2e+006	100.00	1e+008	500, 544

Peak ID	Mass Found	Time
1	Not Found	0.46

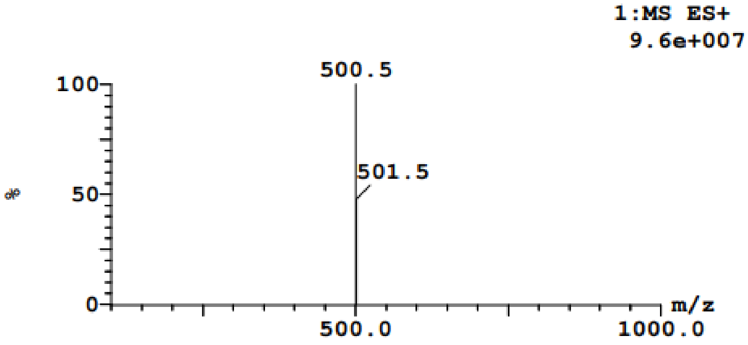

Compound **18**

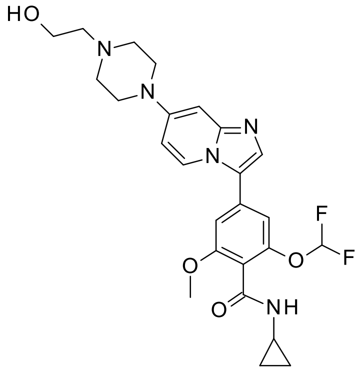

Exact Mass: 501.22
Molecular Formula: $C_{25}H_{29}F_2N_5O_4$

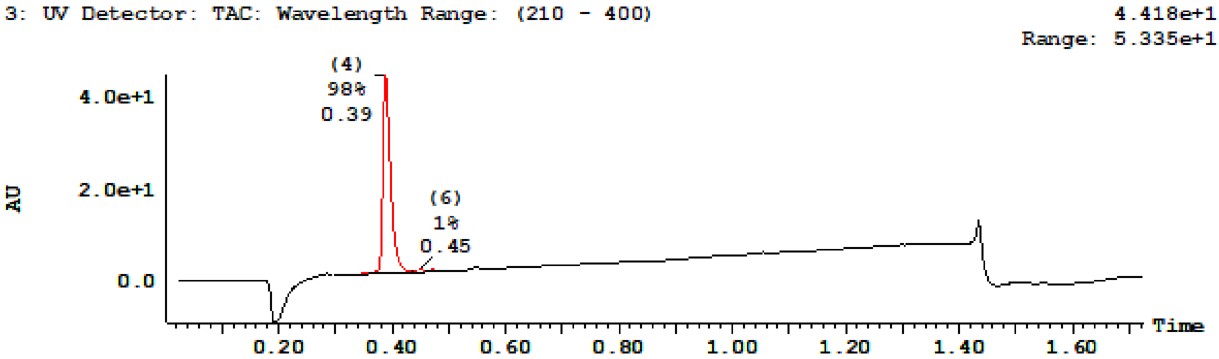

Peak Number	Compound	Time	AreaAbs	Area %Total	Width	Height	Mass Found
4		0.39	7e+005	97.74	0	4e+007	Not Found
6		0.45	1e+004	1.39	0	6e+005	Not Found
7		0.47	6e+003	0.87	0	5e+005	Not Found

Peak ID	Compound	Time	Mass Found
4		0.40	Not Found

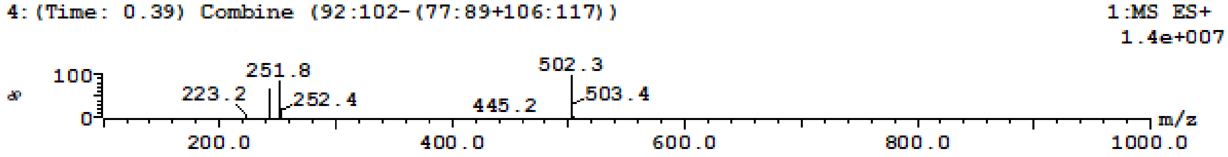

Compound **19**

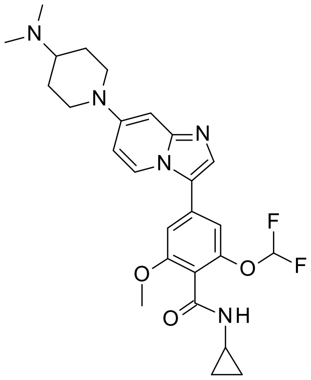

Exact Mass: 499.24
Molecular Formula: $C_{26}H_{31}F_2N_5O_3$

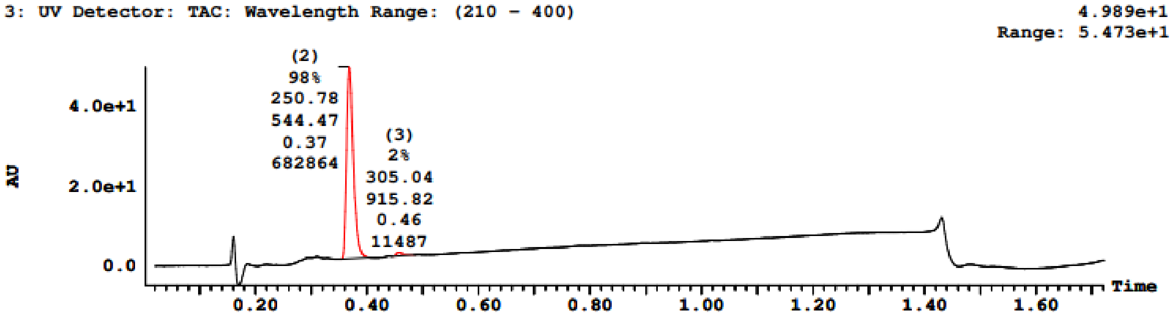

Peak Number	Compound	Time	AreaAbs	Area %Total	Height	BPM
2		0.37	7e+005	98.35	5e+007	251, 544
3		0.46	1e+004	1.65	8e+005	305, 916

Peak ID	Mass Found	Time
2	Not Found	0.38

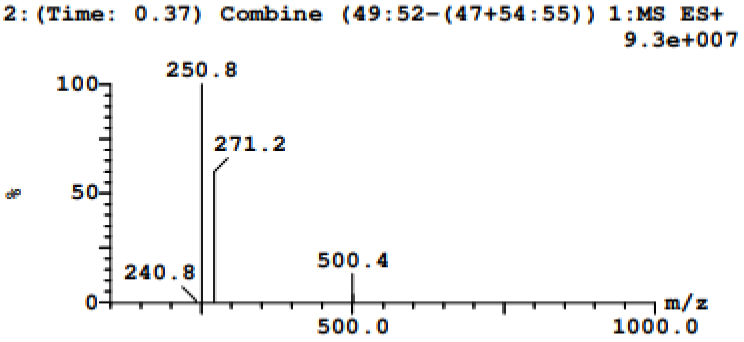

Compound **20**

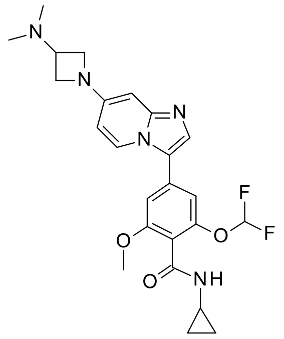

Exact Mass: 471.21
Molecular Formula: $C_{24}H_{27}F_2N_5O_3$

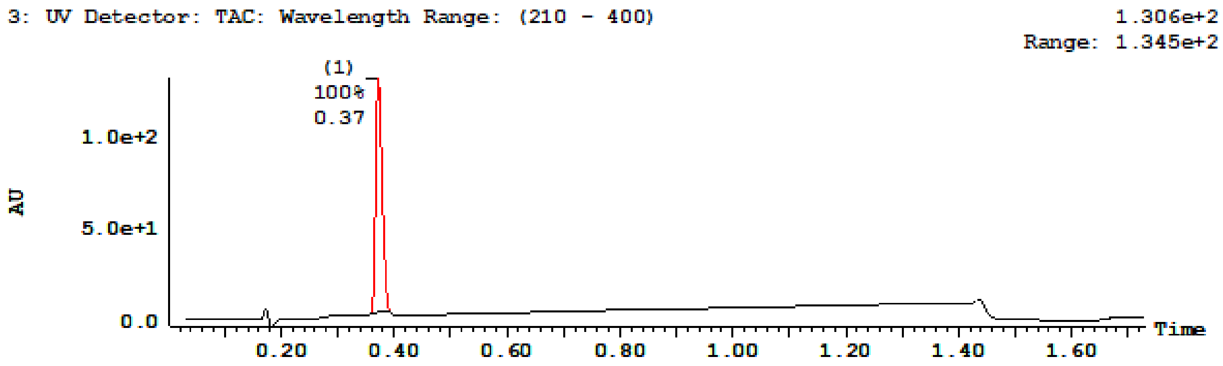

Peak Number	Compound	Time	AreaAbs	Area %Total	Width	Height	Mass Found
1		0.37	2e+006	100.00	0	1e+008	Not Found

Peak ID	Compound	Time	Mass Found
1		0.36	Not Found

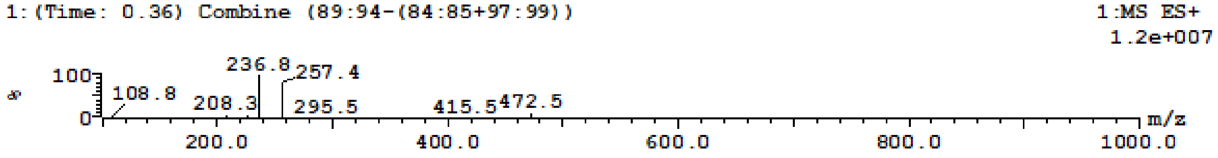

Compound **21**

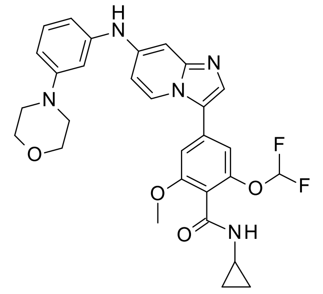

Exact Mass: 549.22
Molecular Formula: $C_{29}H_{29}F_2N_5O_4$

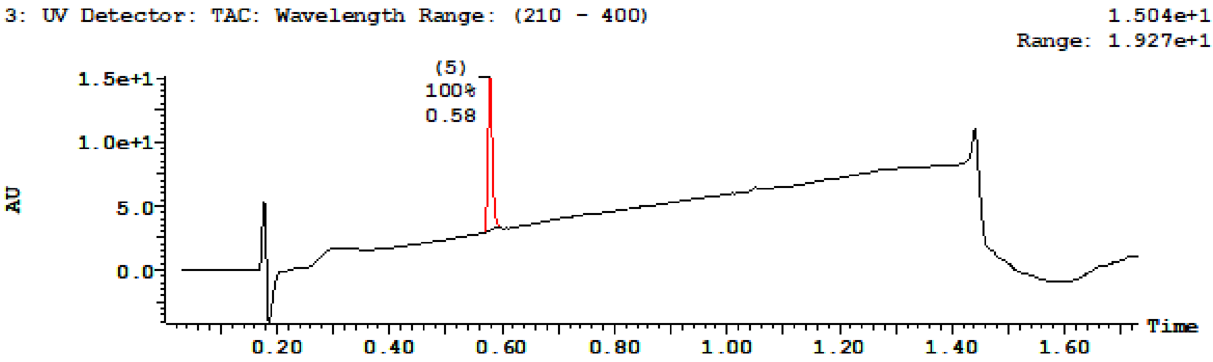

Peak Number	Compound	Time	AreaAbs	Area %Total	Width	Height	Mass Found
5		0.58	1e+005	100.00	0	1e+007	Not Found

Peak ID	Compound	Time	Mass Found
5		0.58	Not Found

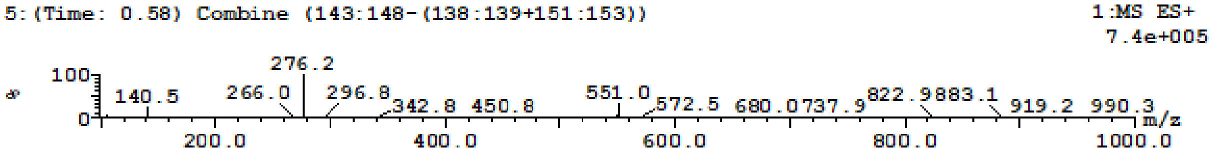

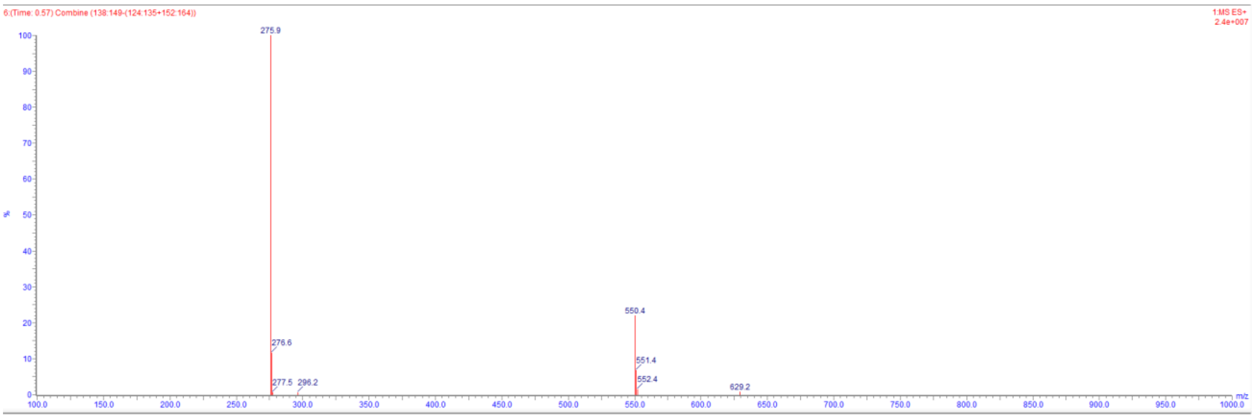

Compound **22**

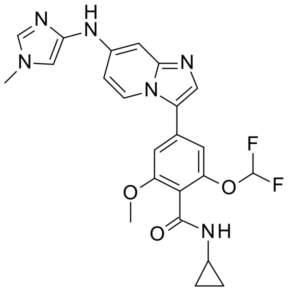

Exact Mass: 468.17
Molecular Formula: $C_{23}H_{22}F_2N_6O_3$

3: UV Detector: TAC: Wavelength Range: (210 - 400) 3.692e+1
Range: 4.597e+1

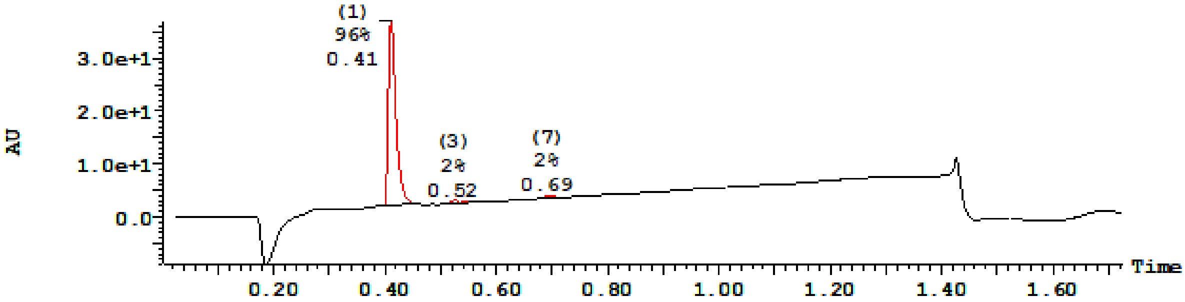

Peak Number	Compound	Time	AreaAbs	Area %Total	Width	Height	Mass Found
1		0.41	6e+005	96.15	0	3e+007	Not Found
3		0.52	1e+004	2.03	0	6e+005	Not Found
7		0.69	1e+004	1.82	0	6e+005	Not Found

Peak ID	Compound	Time	Mass Found
1		0.42	Not Found

1: (Time: 0.41) Combine (97:107-(82:94+111:122)) 1:MS ES+
5.3e+006

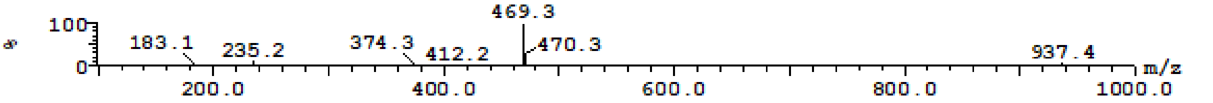

Compound **23**

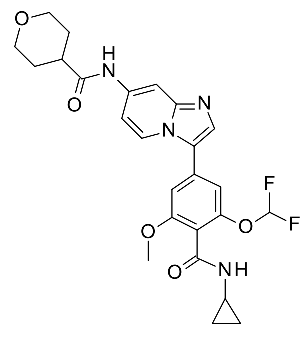

Exact Mass: 500.19
Molecular Formula: $C_{25}H_{26}F_2N_4O_5$

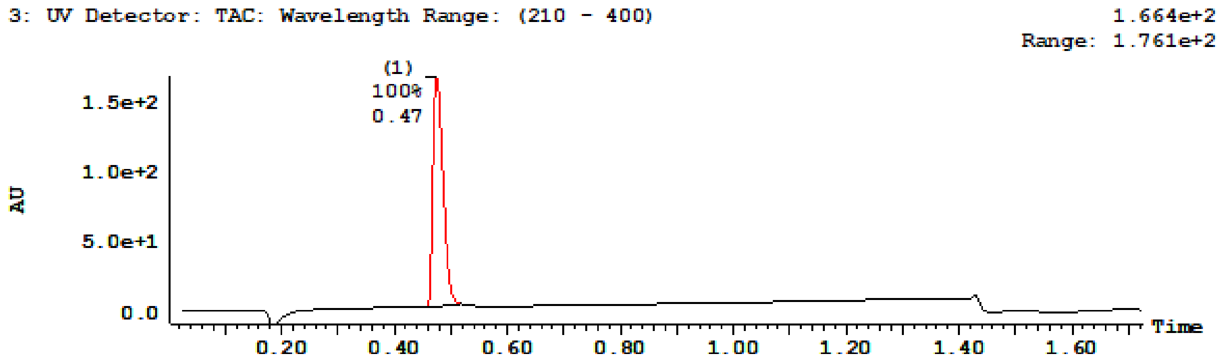

Peak Number	Compound	Time	AreaAbs	Area %Total	Width	Height	Mass Found
1		0.47	3e+006	100.00	0	2e+008	Not Found

Peak ID	Compound	Time	Mass Found
1		0.47	Not Found

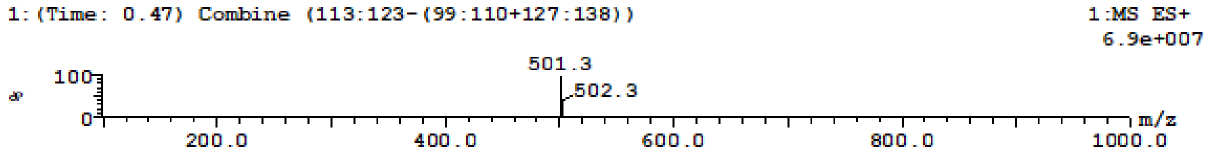

Compound **24**

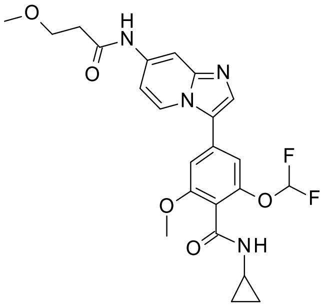

Exact Mass: 474.17
Molecular Formula: $C_{23}H_{24}F_2N_4O_5$

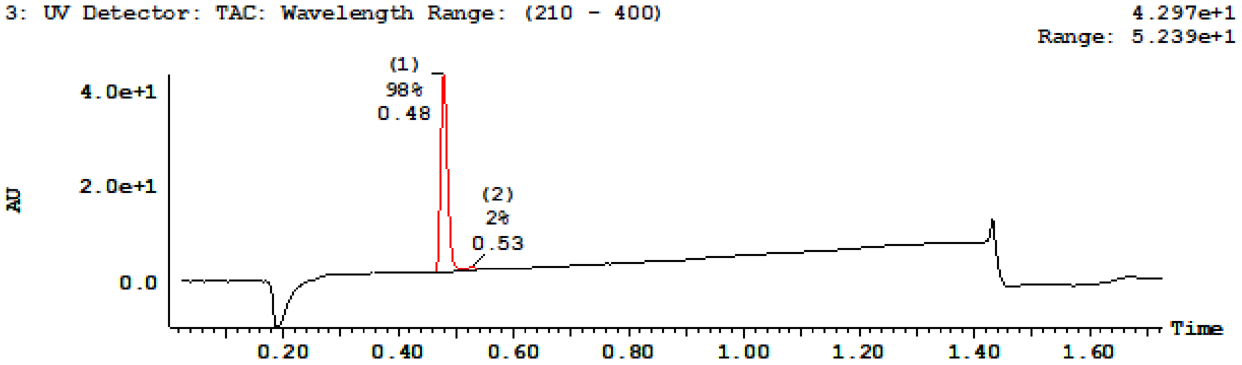

Peak Number	Compound	Time	AreaAbs	Area %Total	Width	Height	Mass Found
1		0.48	6e+005	98.15	0	4e+007	Not Found
2		0.53	1e+004	1.85	0	7e+005	Not Found

Peak ID	Compound	Time	Mass Found
1		0.49	Not Found

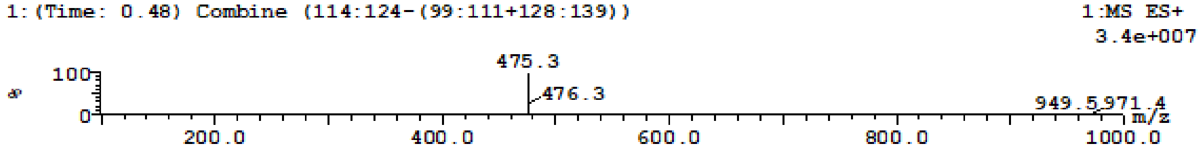

Compound **25**

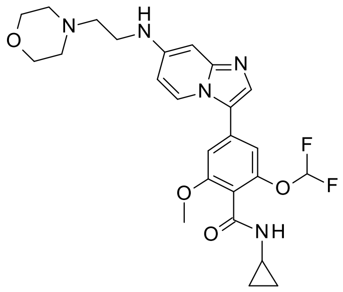

Exact Mass: 501.22
Molecular Formula: $C_{25}H_{29}F_{2}N_{5}O_{4}$

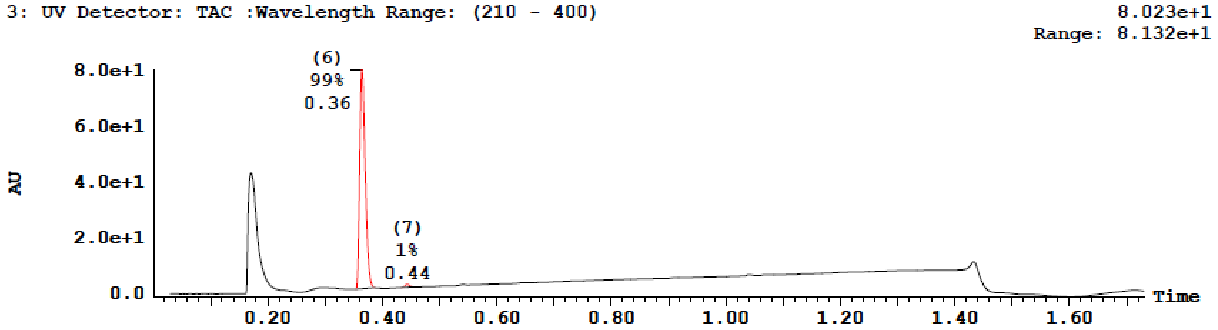

Peak Number	Compound	Time	AreaAbs	Area %Total	Width	Height	Mass Found
6		0.36	9e+005	98.92	0	8e+007	Not Found
7		0.44	1e+004	1.08	0	1e+006	Not Found

Peak ID	Compound	Time	Mass Found
6		0.37	Not Found

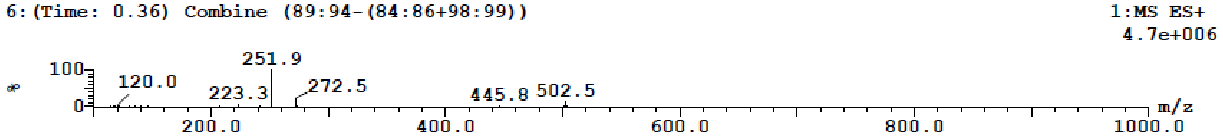

Compound **26**

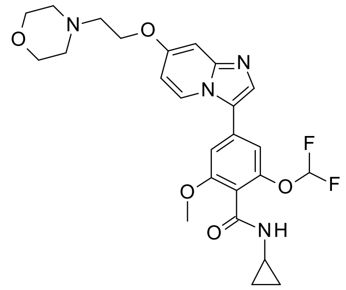

Exact Mass: 502.2
Molecular Formula: $C_{25}H_{28}F_2N_4O_5$

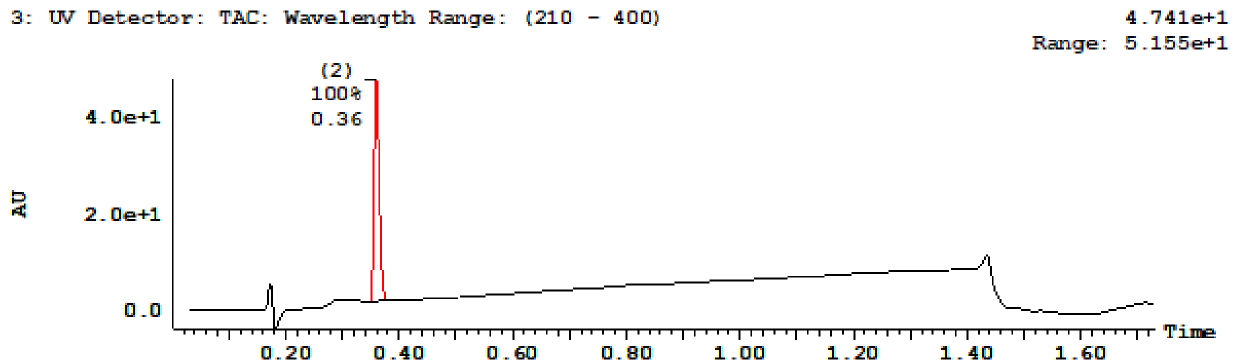

Peak Number	Compound	Time	AreaAbs	Area %Total	Width	Height	Mass Found
2		0.36	5e+005	100.00	0	5e+007	Not Found

Peak ID	Compound	Time	Mass Found
2		0.36	Not Found

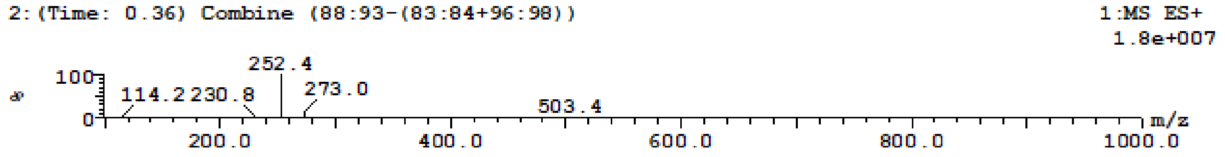

Compound **27**

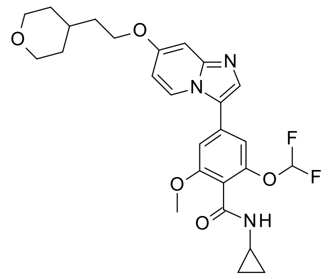

Exact Mass: 501.21
Molecular Formula: $C_{26}H_{29}F_2N_3O_5$

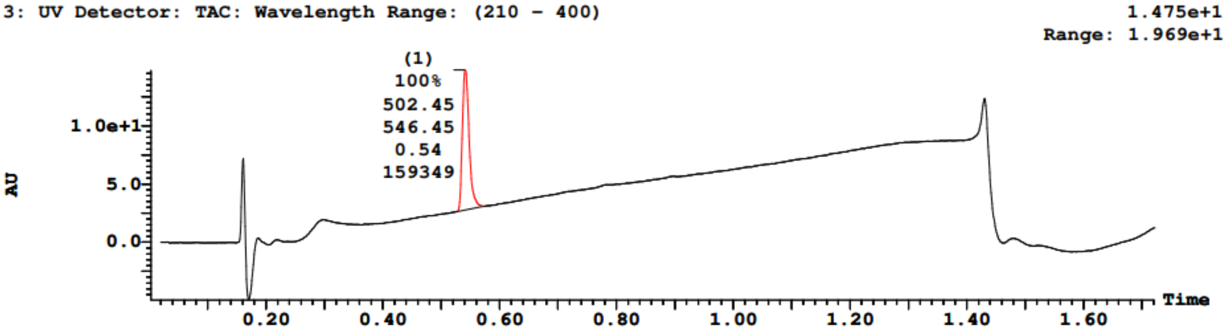

Peak Number	Compound	Time	AreaAbs	Area %Total	Height	BPM
1		0.54	2e+005	100.00	1e+007	502, 546

Peak ID	Mass Found	Time
1	Not Found	0.55

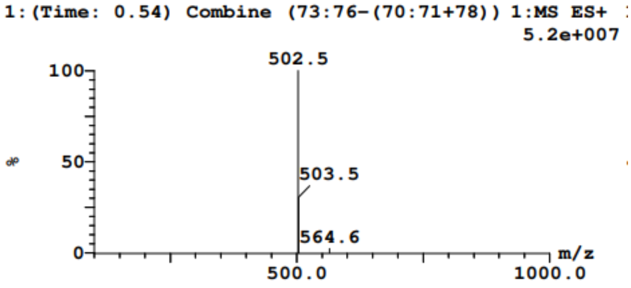

Compound **28**

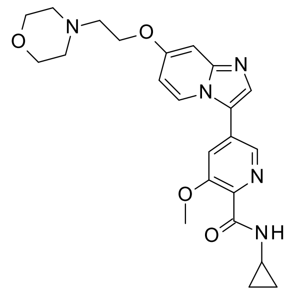

Exact Mass: 437.21
Molecular Formula: $C_{23}H_{27}N_5O_4$

3: UV Detector: TAC: Wavelength Range: (210 - 400)

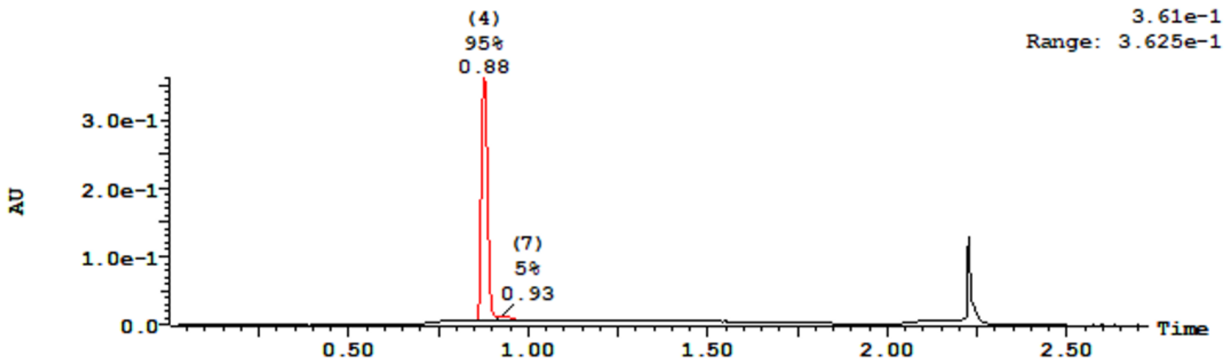

Peak Number	Compound	Time	AreaAbs	Area %Total	Width	Height	Mass Found
4		0.88	7e+003	95.18	0	4e+005	Not Found
7		0.93	4e+002	4.82	0	8e+003	Not Found

Peak ID	Compound	Time	Mass Found
4		0.88	Not Found

4: (Time: 0.88) Combine (214:224-(199:211+228:239))

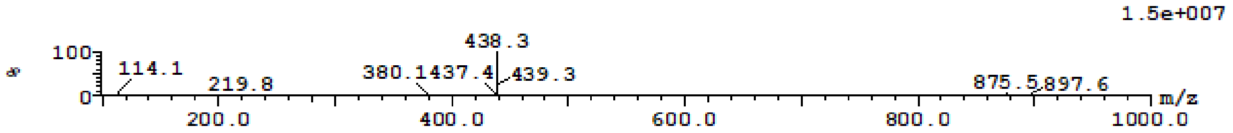

Compound **29**

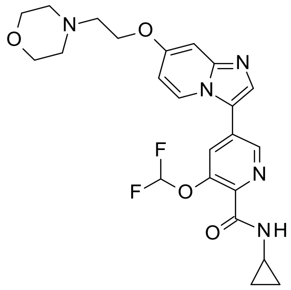

Exact Mass: 473.19
Molecular Formula: $C_{23}H_{25}F_2N_5O_4$

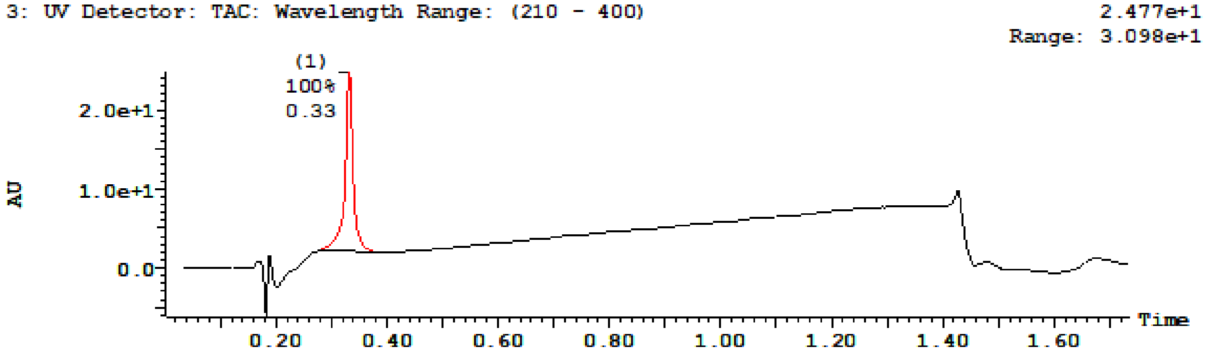

Peak Number	Compound	Time	AreaAbs	Area %Total	Width	Height	Mass Found
1		0.33	4e+005	100.00	0	2e+007	Not Found

Peak ID	Compound	Time	Mass Found
1		0.33	Not Found

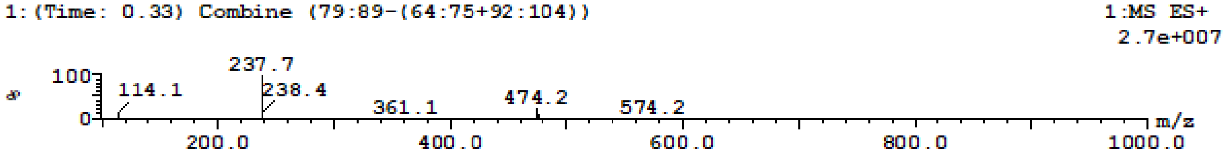

Compound **30**

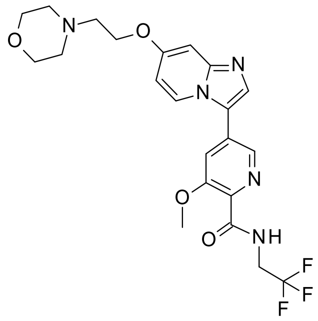

Exact Mass: 479.18
Molecular Formula: $C_{22}H_{24}F_3N_5O_4$

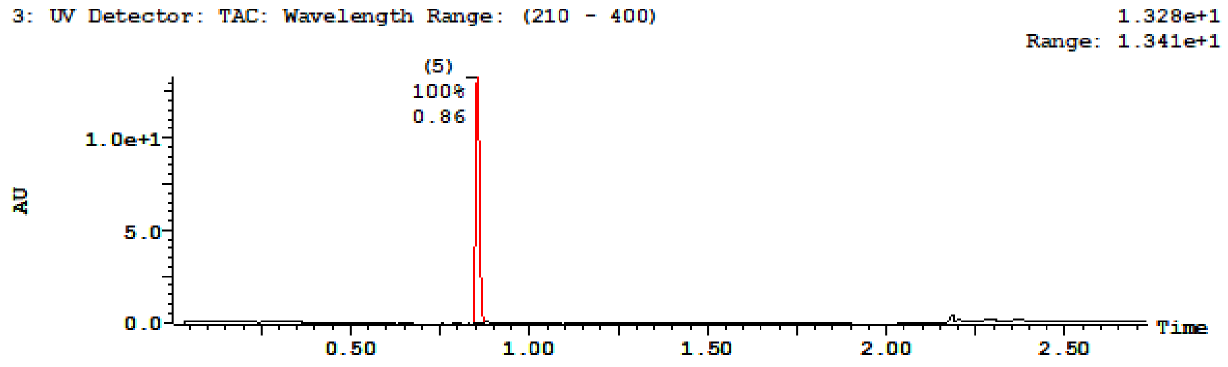

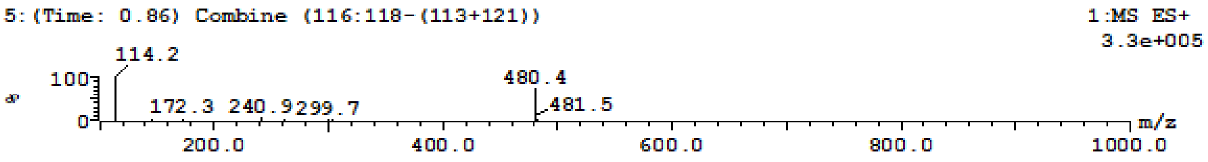

Compound **31**

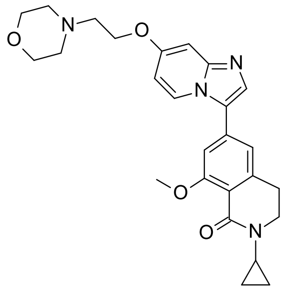

Exact Mass: 462.23
Molecular Formula: $C_{26}H_{30}N_4O_4$

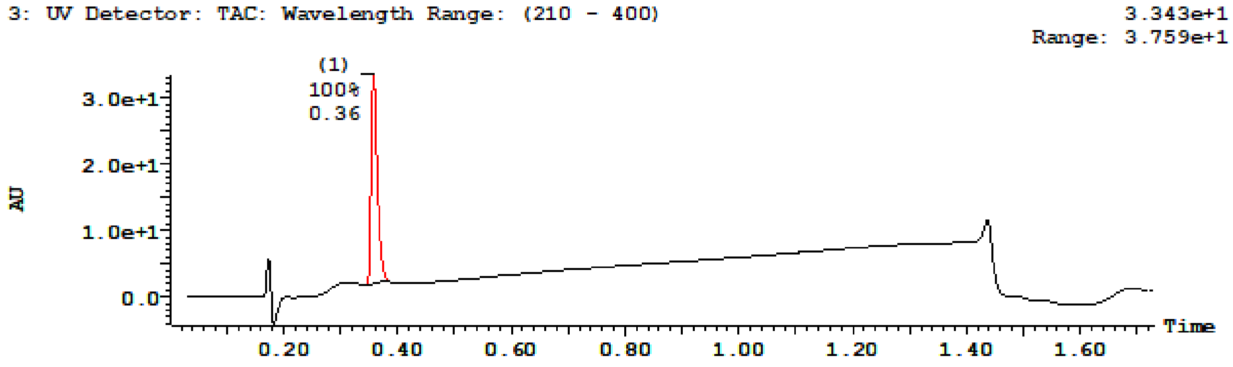

Peak ID	Compound	Time	Mass Found
1		0.36	Not Found

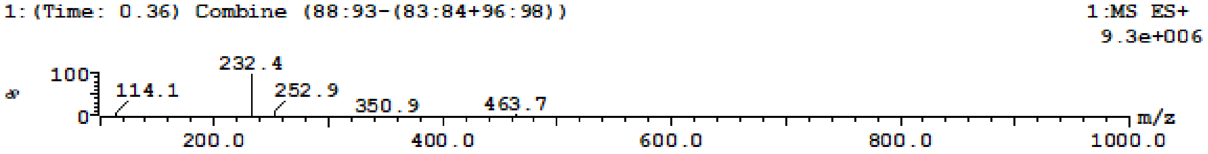

Compound **32**

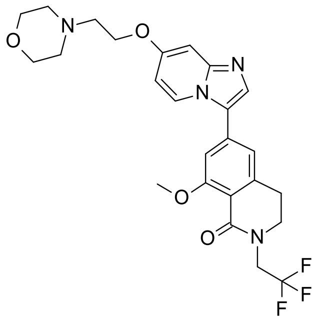

Exact Mass: 504.2
Molecular Formula: $C_{25}H_{27}F_3N_4O_4$

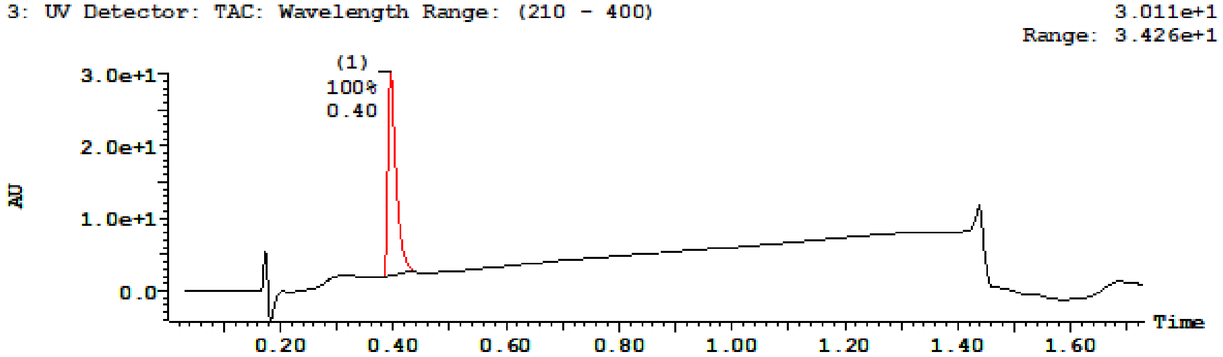

Peak Number	Compound	Time	AreaAbs	Area %Total	Width	Height	Mass Found
1		0.40	5e+005	100.00	0	3e+007	Not Found

Peak ID	Compound	Time	Mass Found
1		0.39	Not Found

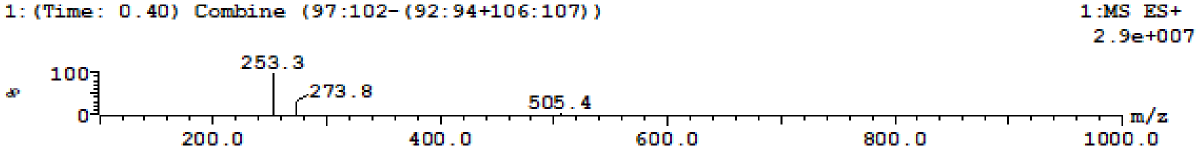

Synthetic intermediates

Compound **36**

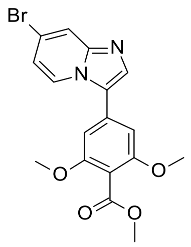

Exact Mass: 390.02
Molecular Formula: $C_{17}H_{15}BrN_2O_4$

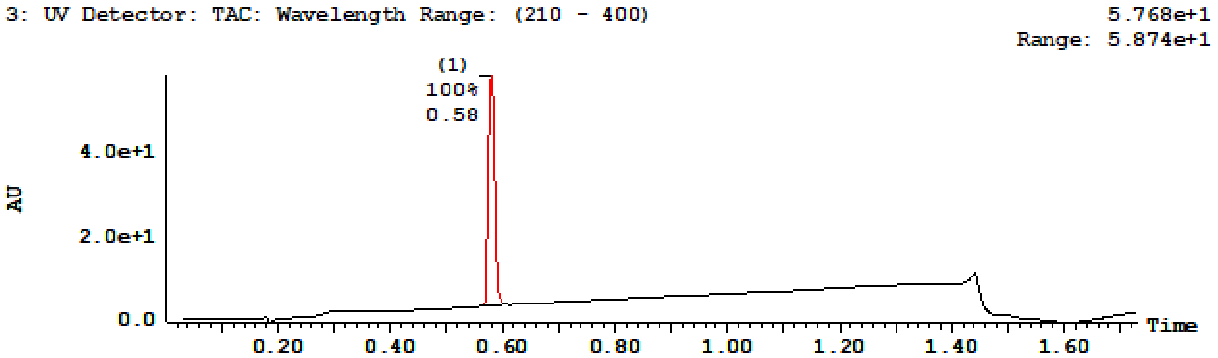

Peak Number	Compound	Time	AreaAbs	Area %Total	Width	Height	Mass Found
1		0.58	7e+005	100.00	0	5e+007	Not Found

Peak ID	Compound	Time	Mass Found
1		0.57	Not Found

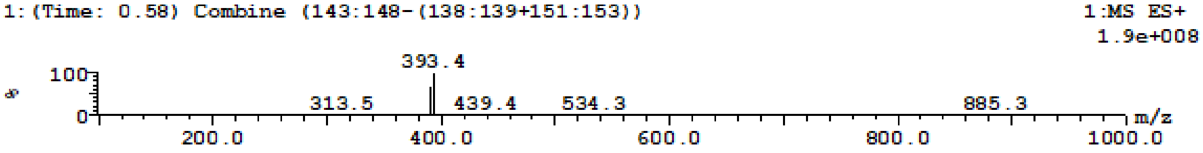

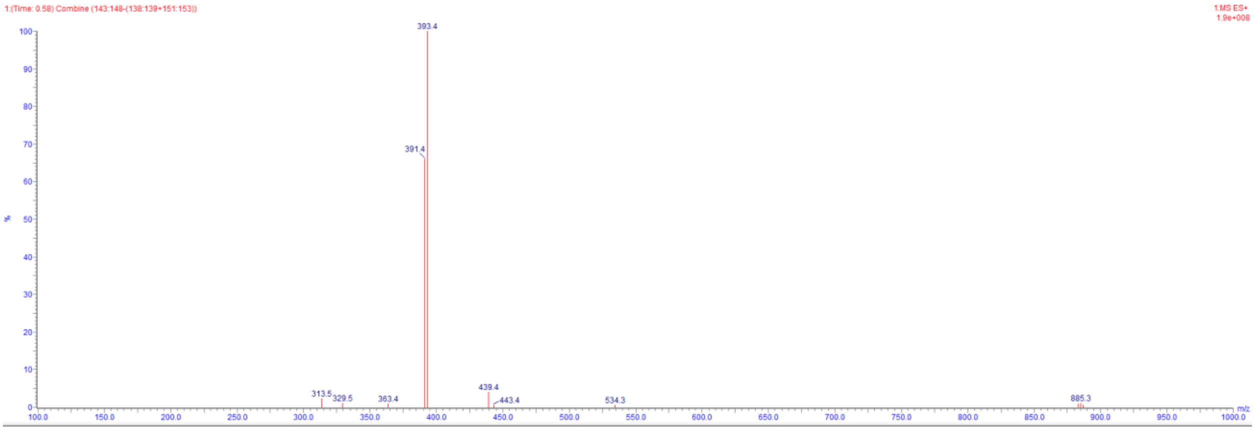

Compound **37**

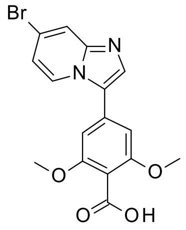

Exact Mass: 376.01
Molecular Formula: $C_{16}H_{13}BrN_2O_4$

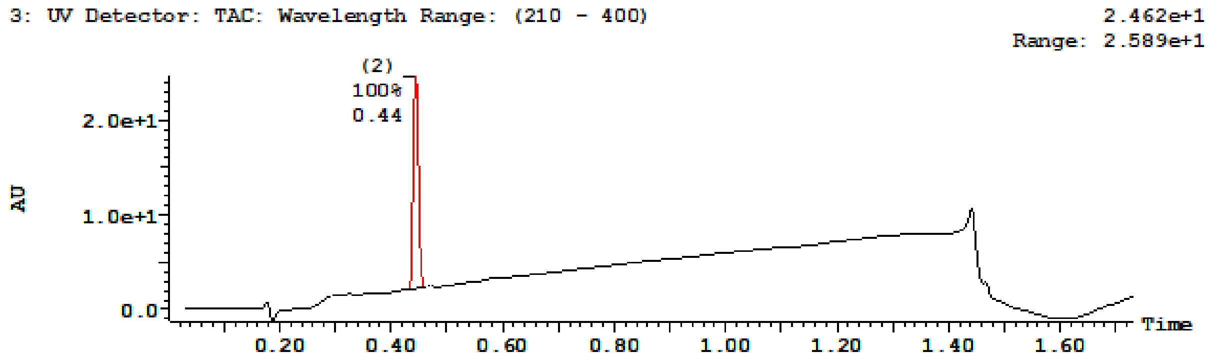

Peak Number	Compound	Time	AreaAbs	Area %Total	Width	Height	Mass Found
2		0.44	2e+005	100.00	0	2e+007	Not Found

Peak ID	Compound	Time	Mass Found
2		0.44	Not Found

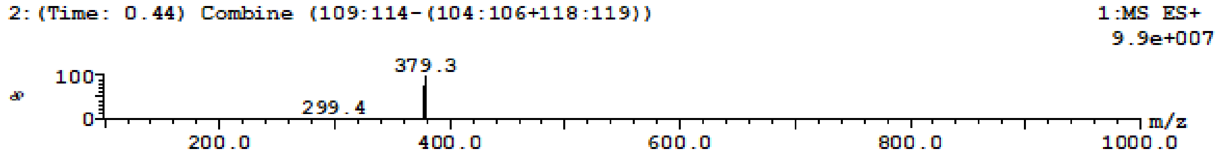

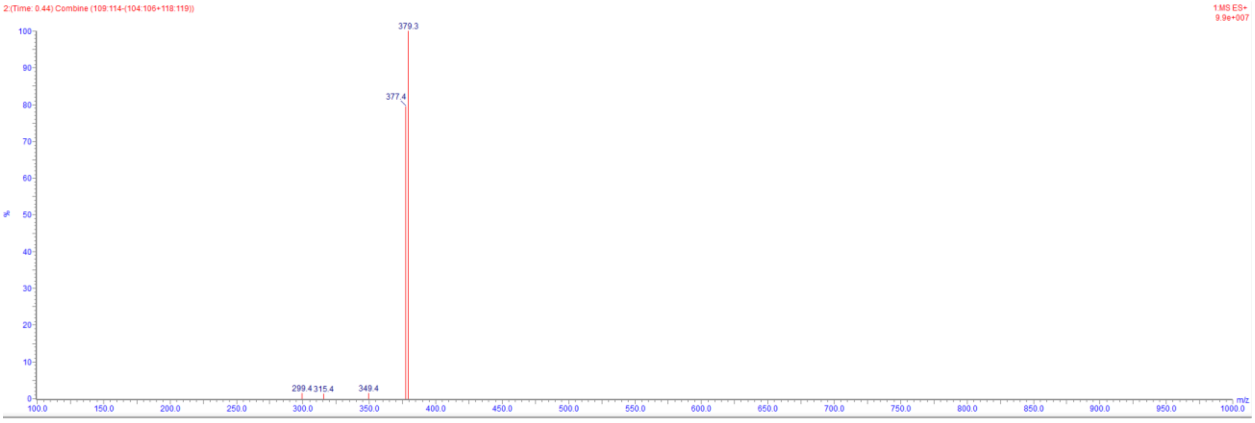

Compound **38**

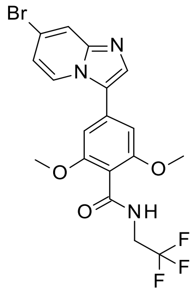

Exact Mass: 457.02
Molecular Formula: $C_{18}H_{15}BrF_3N_3O_3$

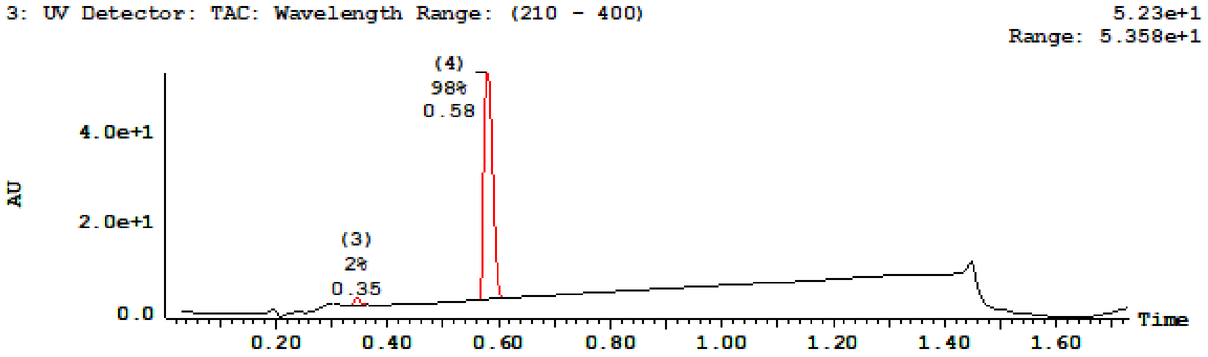

Peak ID	Compound	Time	Mass Found
4		0.58	Not Found

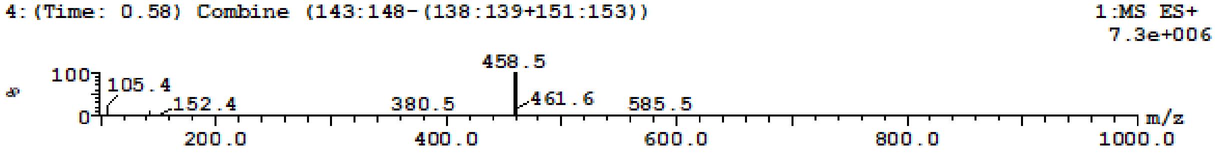

Compound **40**

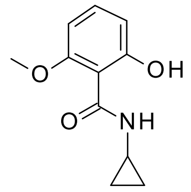

Exact Mass: 207.09
Molecular Formula: $C_{11}H_{13}NO_3$

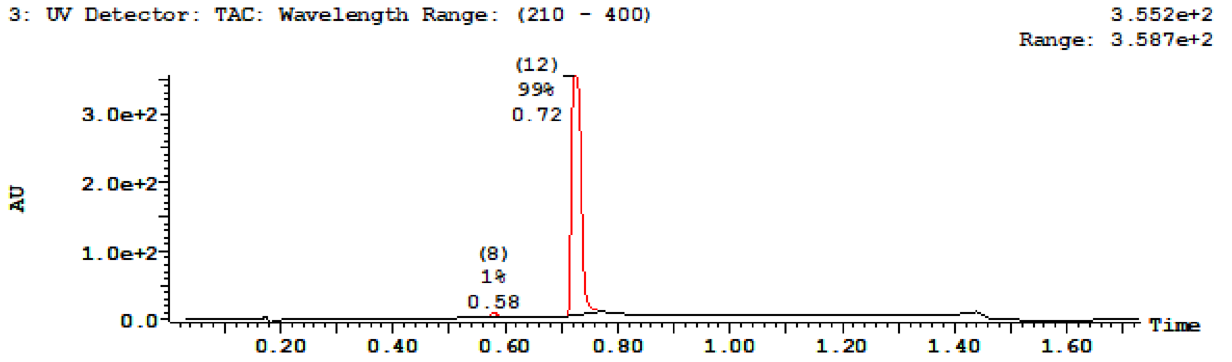

Peak Number	Compound	Time	AreaAbs	Area %Total	Width	Height	Mass Found
8		0.58	9e+004	1.24	0	7e+006	Not Found
12		0.72	7e+006	98.76	0	3e+008	Not Found

Peak ID	Compound	Time	Mass Found
12		0.73	Not Found

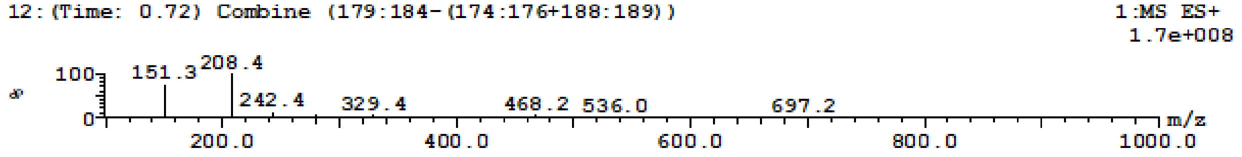

Compound **41**

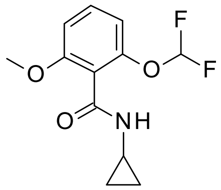

Exact Mass: 257.09
Molecular Formula: $C_{12}H_{13}F_2NO_3$

3: UV Detector: TAC: Wavelength Range: (210 - 400)

4.293e+1
Range: 4.722e+1

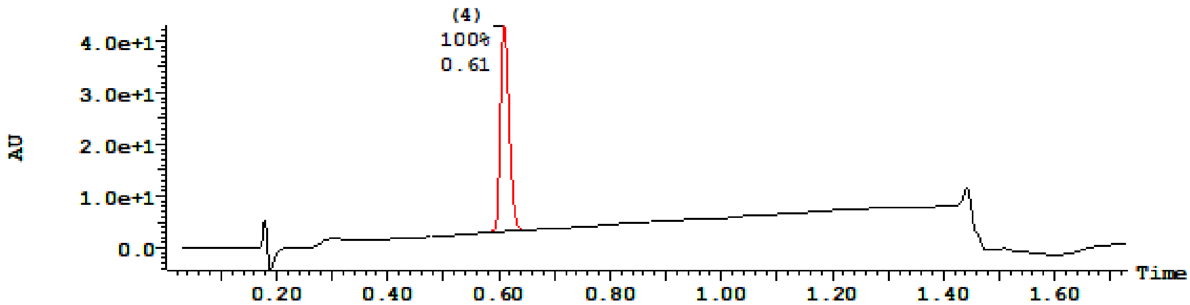

Peak Number	Compound	Time	AreaAbs	Area %Total	Width	Height	Mass Found
4		0.61	7e+005	100.00	0	4e+007	Not Found

Peak ID	Compound	Time	Mass Found
4		0.61	Not Found

4: (Time: 0.61) Combine (150:155-(145:147+159:160))

1:MS ES+
1.9e+008

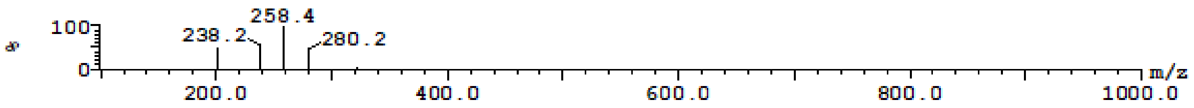

Compound **42**

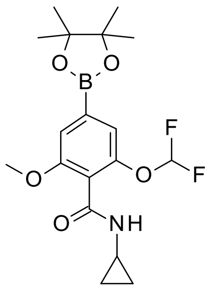

Exact Mass: 383.17
Molecular Formula: $C_{18}H_{24}BF_2NO_5$

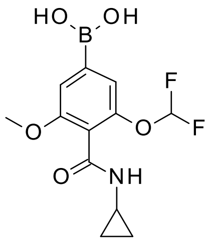

Exact Mass: 301.09
Molecular Formula: $C_{12}H_{14}BF_2NO_5$

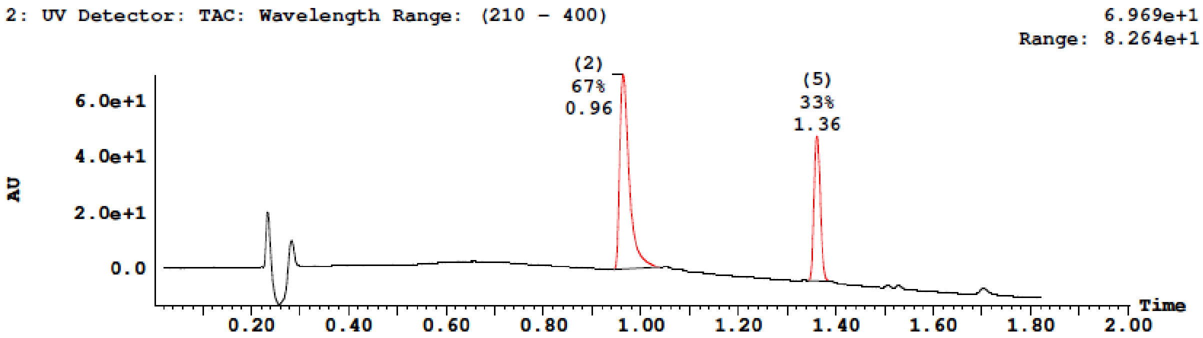

Peak Number	Compound	Time	AreaAbs	Area %Total	Width	Height	Mass Found
2		0.96	2e+006	67.44	0	7e+007	Not Found
5		1.36	8e+005	32.56	0	5e+007	Not Found

Peak ID	Compound	Time	Mass Found
2		0.96	Not Found

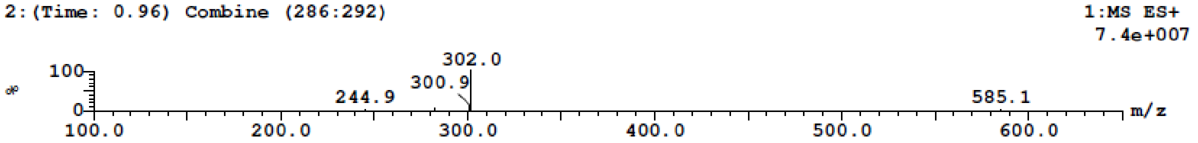

Peak ID	Compound	Time	Mass Found
5		1.36	Not Found

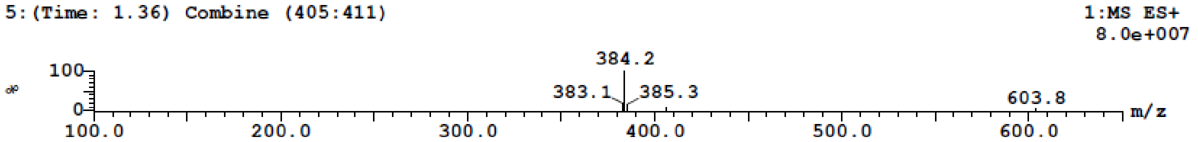

Compound **43a**

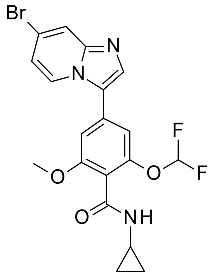

Exact Mass: 451.03
Molecular Formula: $C_{19}H_{16}BrF_2N_3O_3$

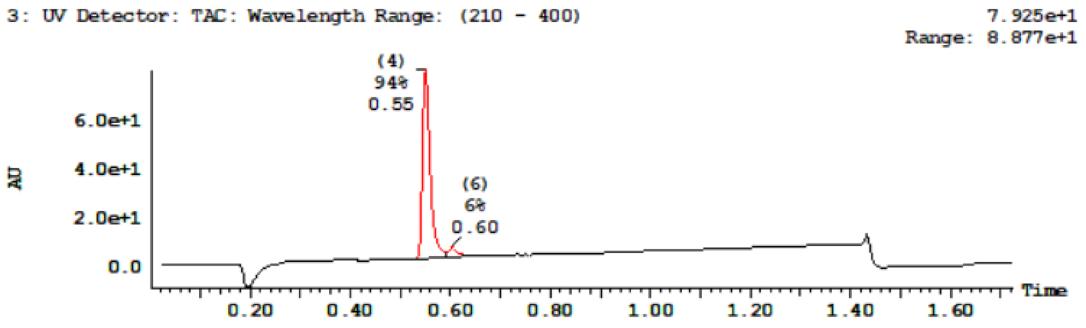

PeakID	Compound	Time	Mass Found
4		0.56	Not Found

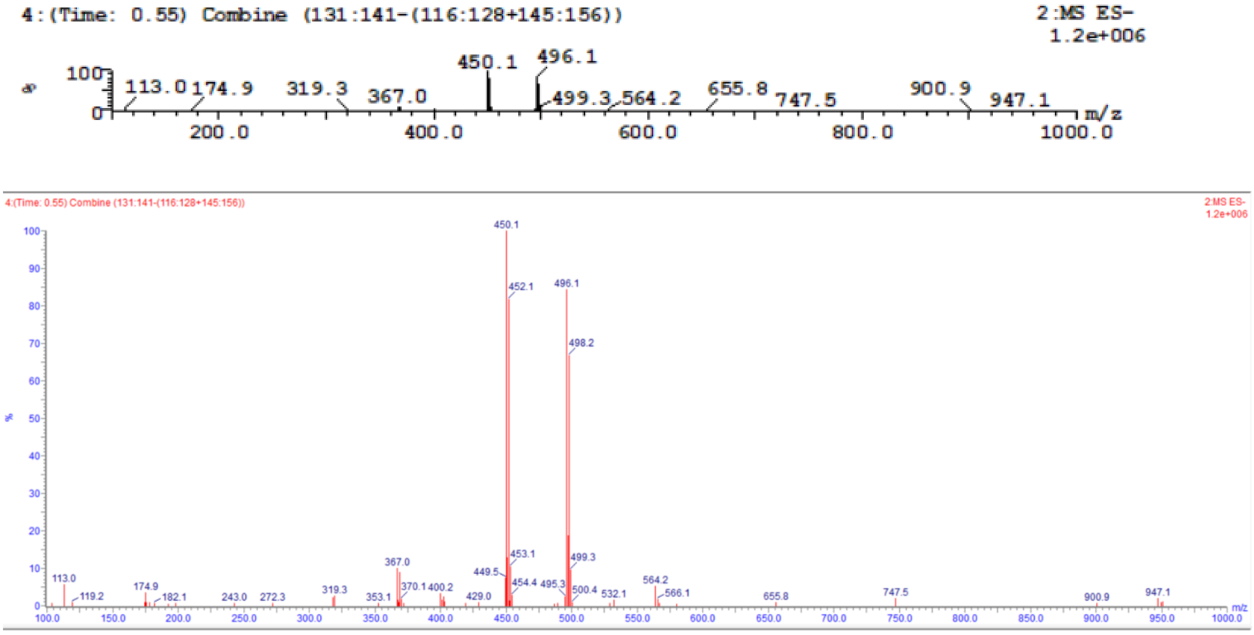

Compound **43b**

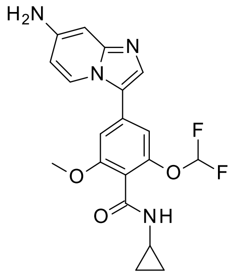

Exact Mass: 388.13
Molecular Formula: $C_{19}H_{18}F_2N_4O_3$

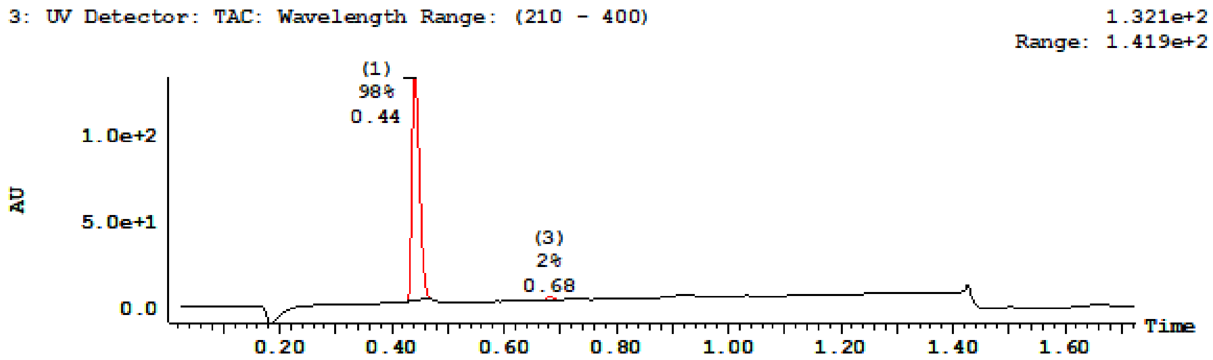

Peak Number	Compound	Time	AreaAbs	Area %Total	Width	Height	Mass Found
1		0.44	2e+006	98.42	0	1e+008	Not Found

Peak ID	Compound	Time	Mass Found
1		0.44	Not Found

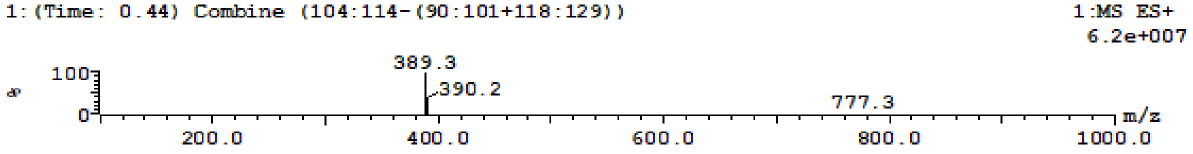

Compound **43c**

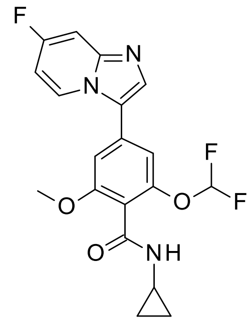

Exact Mass: 391.11
Molecular Formula: $C_{19}H_{16}F_3N_3O_3$

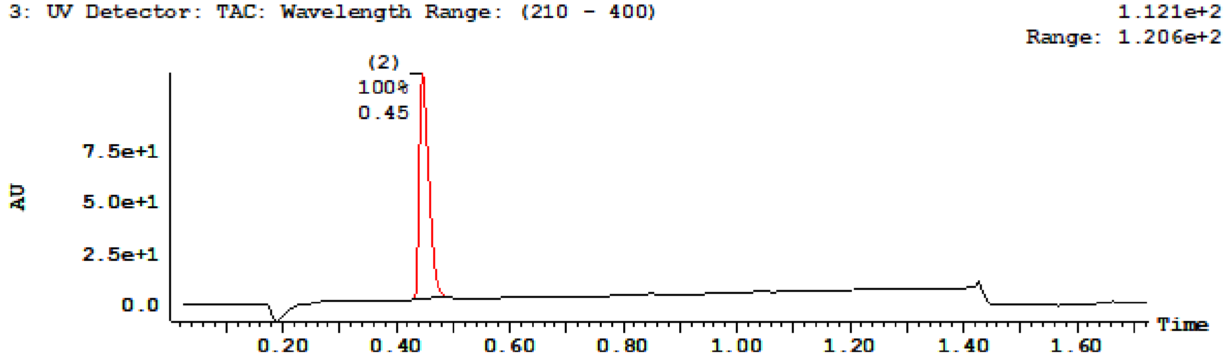

Peak Number	Compound	Time	AreaAbs	Area %Total	Width	Height	Mass Found
2		0.45	2e+006	100.00	0	1e+008	Not Found

Peak ID	Compound	Time	Mass Found
2		0.45	Not Found

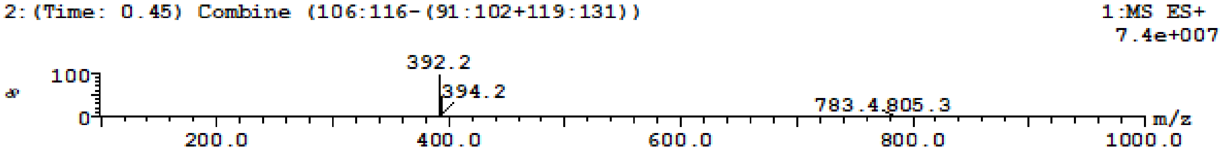

Compound **45**

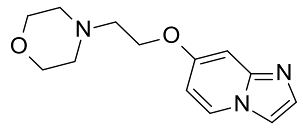

Exact Mass: 247.13
Molecular Formula: $C_{13}H_{17}N_3O_2$

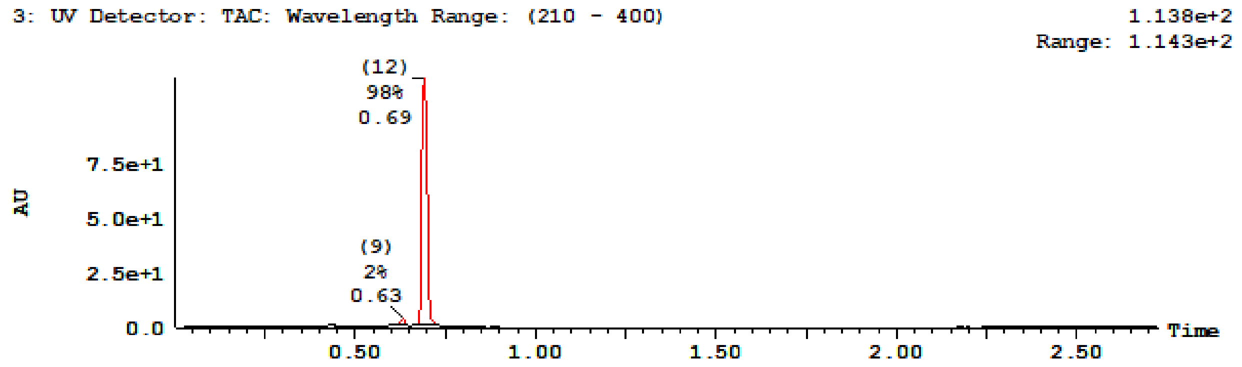

Peak Number	Compound	Time	AreaAbs	Area %Total	Width	Height	Mass Found
9		0.63	4e+004	2.01	0	3e+006	Not Found
12		0.69	2e+006	97.99	0	1e+008	Not Found

Peak ID	Compound	Time	Mass Found
12		0.70	Not Found

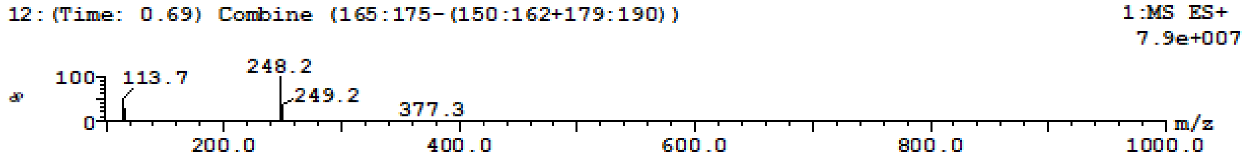

Compound **47a**

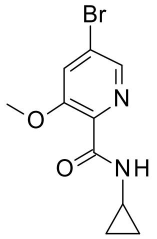

Exact Mass: 270
Molecular Formula: $C_{10}H_{11}BrN_2O_2$

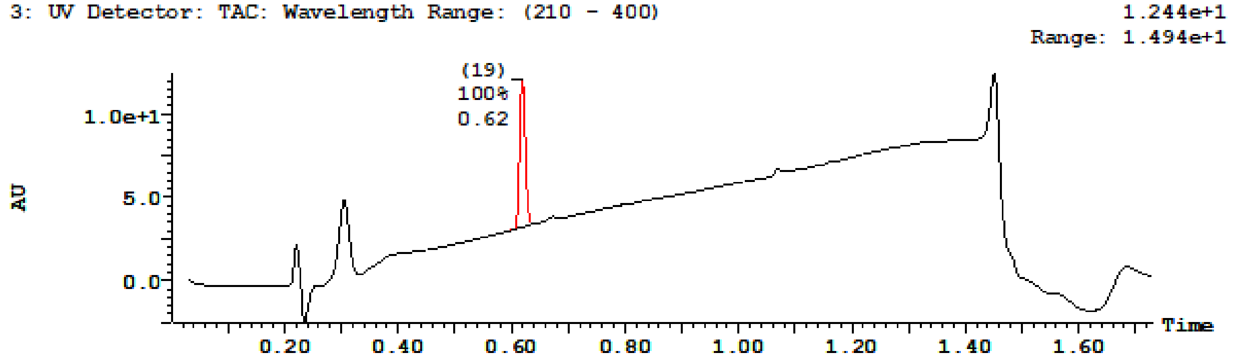

Peak Number	Compound	Time	AreaAbs	Area %Total	Width	Height	Mass Found
19		0.62	1e+005	100.00	0	9e+006	Not Found

Peak ID	Compound	Time	Mass Found
19		0.62	Not Found

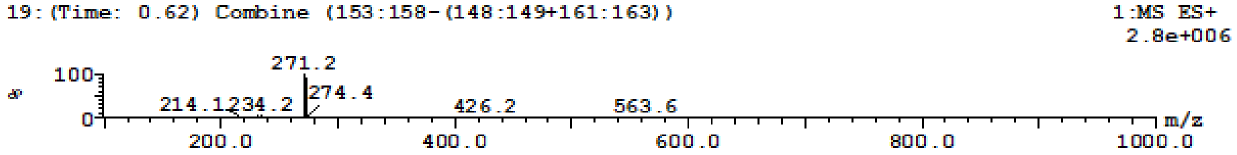

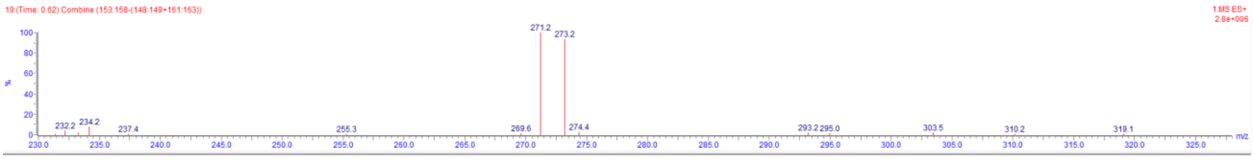

Compound **47b**

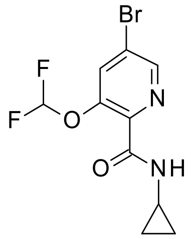

Exact Mass: 305.98

Molecular Formula: $C_{10}H_{9}BrF_{2}N_{2}O_{2}$

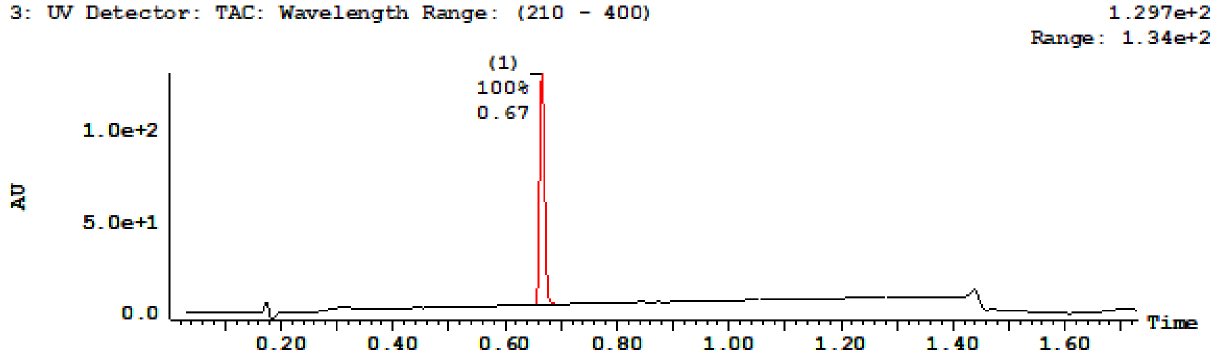

Peak Number	Compound	Time	AreaAbs	Area %Total	Width	Height	Mass Found
1		0.67	1e+006	100.00	0	1e+008	Not Found

Peak ID	Compound	Time	Mass Found
1		0.67	Not Found

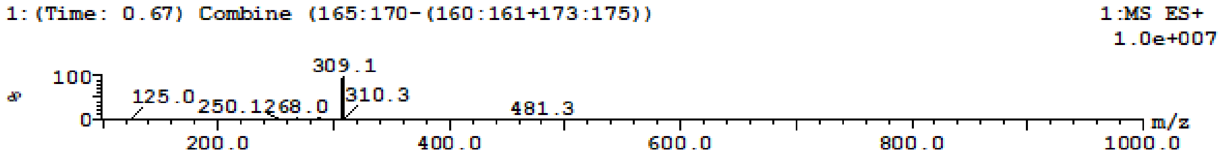

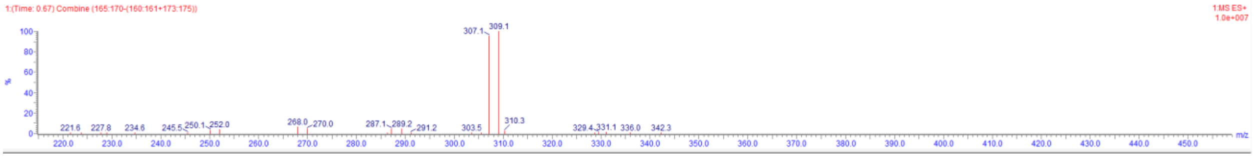

Compound **47c**

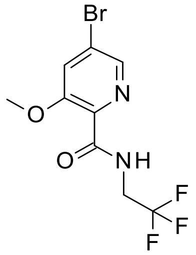

Exact Mass: 311.97
Molecular Formula: $C_9H_8BrF_3N_2O_2$

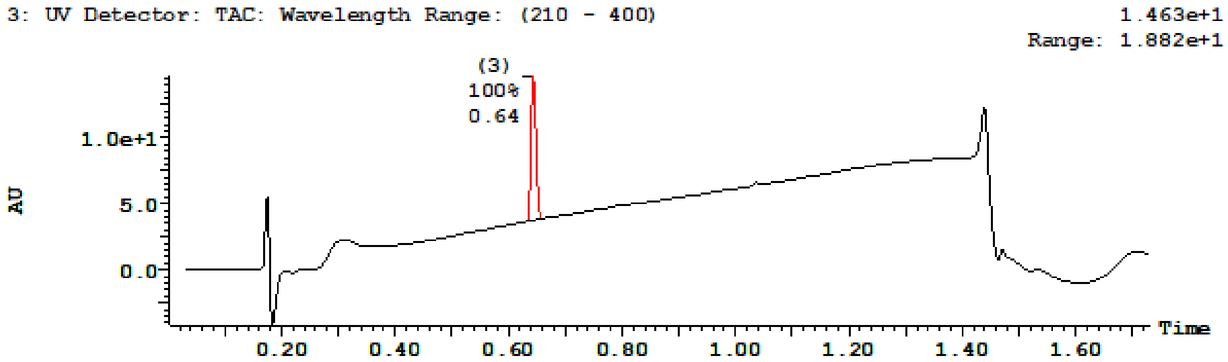

Peak Number	Compound	Time	AreaAbs	Area %Total	Width	Height	Mass Found
3		0.64	1e+005	100.00	0	1e+007	Not Found

Peak ID	Compound	Time	Mass Found
3		0.65	Not Found

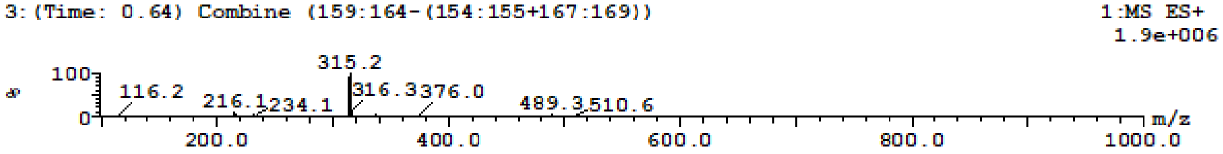

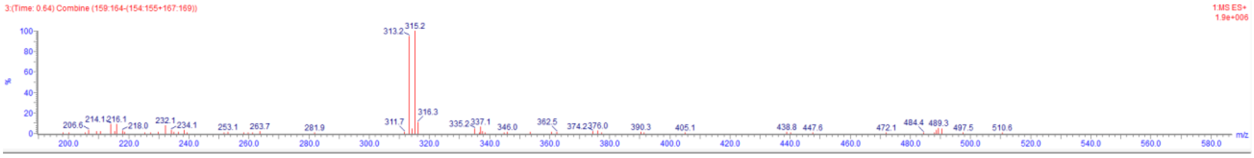

Compound **49**

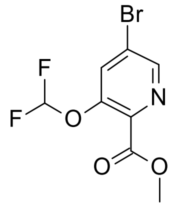

Exact Mass: 280.95
Molecular Formula: $C_8H_6BrF_2NO_3$

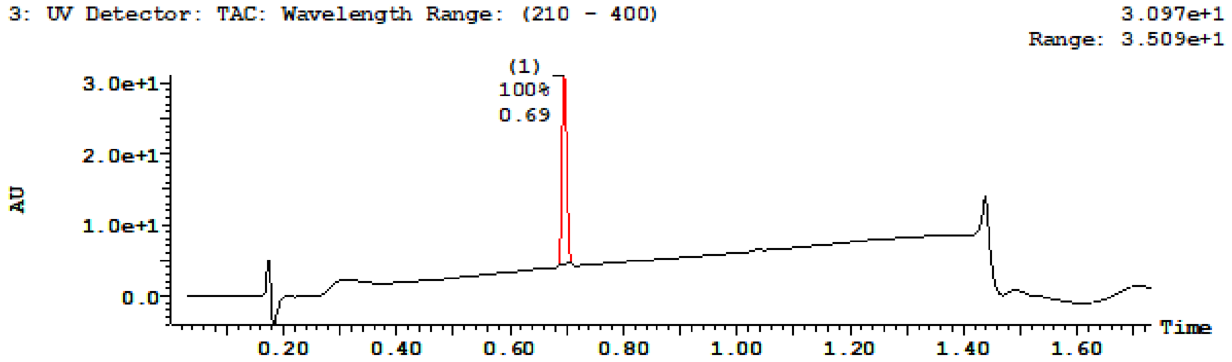

Peak Number	Compound	Time	AreaAbs	Area %Total	Width	Height	Mass Found
1		0.69	3e+005	100.00	0	3e+007	Not Found

Peak ID	Compound	Time	Mass Found
1		0.69	Not Found

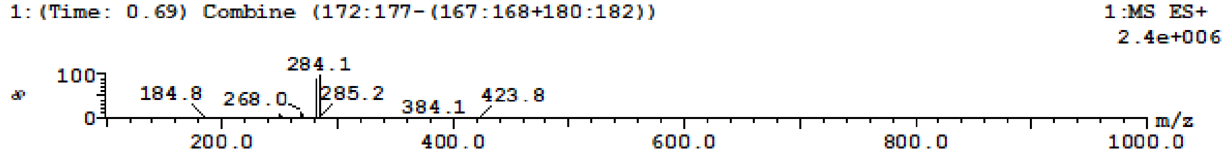

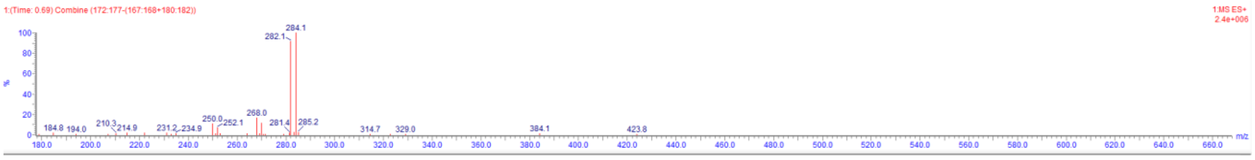

Compound **50**

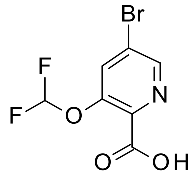

Exact Mass: 266.93
Molecular Formula: $C_7H_4BrF_2NO_3$

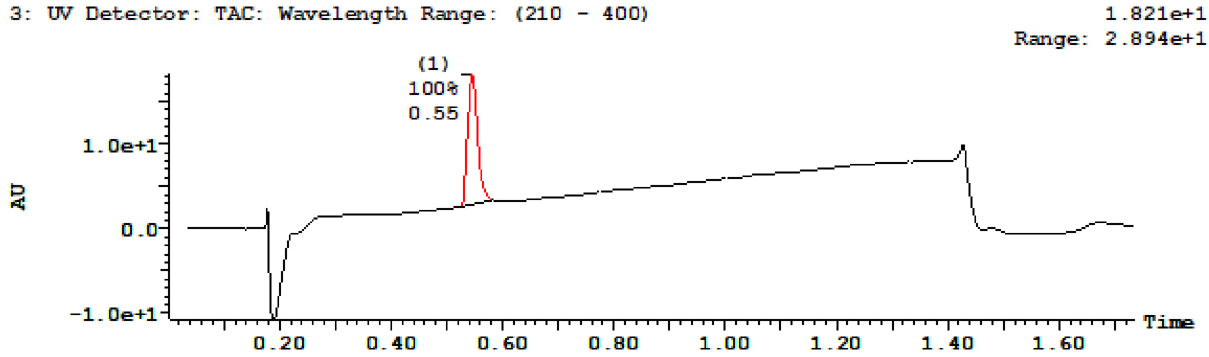

Peak Number	Compound	Time	AreaAbs	Area %Total	Width	Height	Mass Found
1		0.55	3e+005	100.00	0	2e+007	Not Found

Peak ID	Compound	Time	Mass Found
1		0.54	Not Found

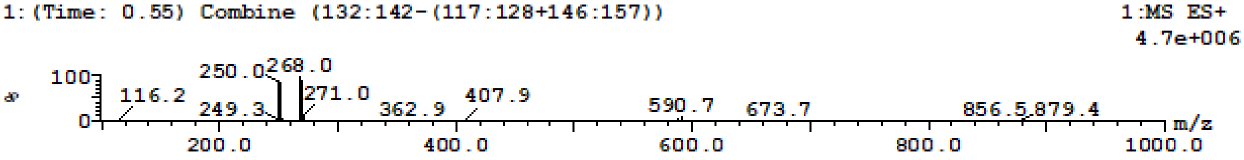

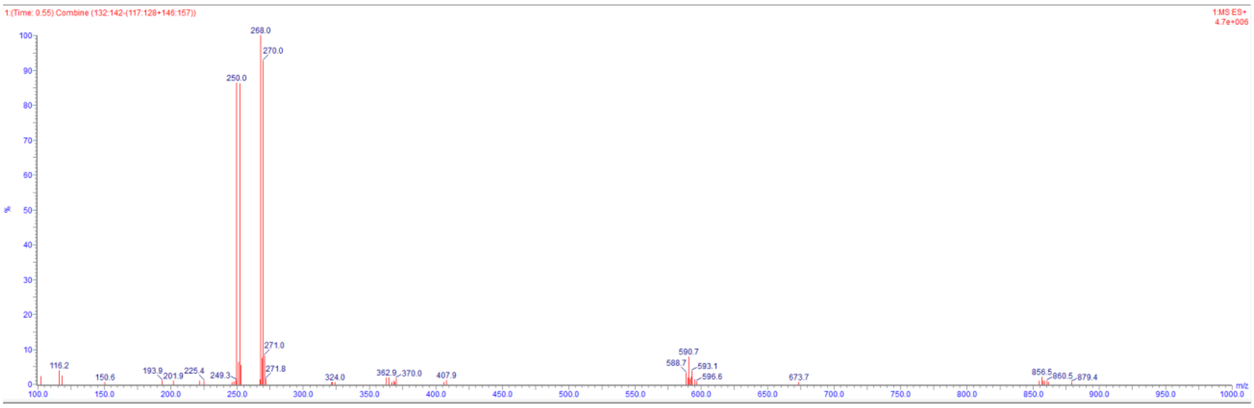

Compound **52**

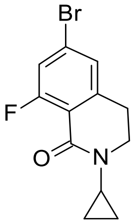

Exact Mass: 283
Molecular Formula: $C_{12}H_{11}BrFNO$

3: UV Detector: TAC: Wavelength Range: (210 - 400) 1.257e+2
Range: 1.31e+2

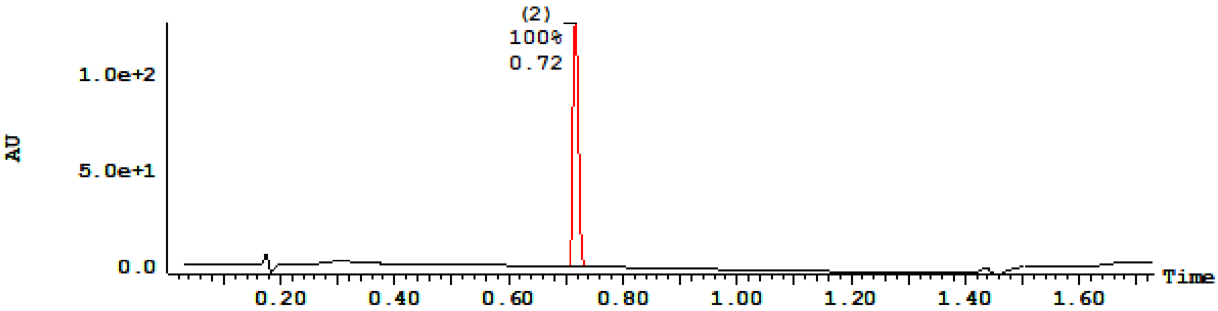

Peak Number	Compound	Time	AreaAbs	Area %Total	Width	Height	Mass Found
2		0.72	1e+006	100.00	0	1e+008	Not Found

Peak ID	Compound	Time	Mass Found
2		0.71	Not Found

2: (Time: 0.72) Combine (177:182-(172:174+186:187)) 1:MS ES+
1.4e+007

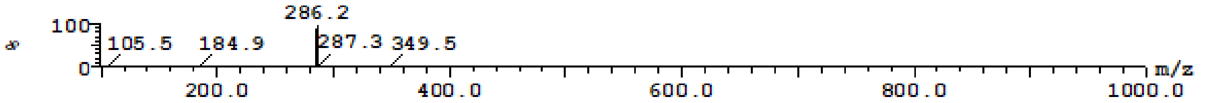

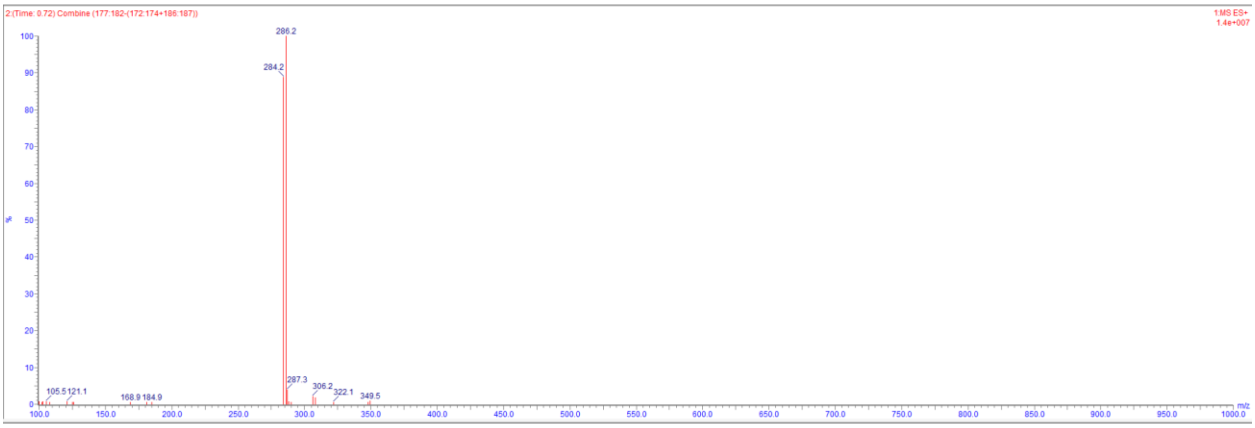

Compound **53a**

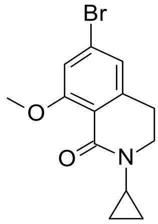

Exact Mass: 295.02
Molecular Formula: $C_{13}H_{14}BrNO_2$

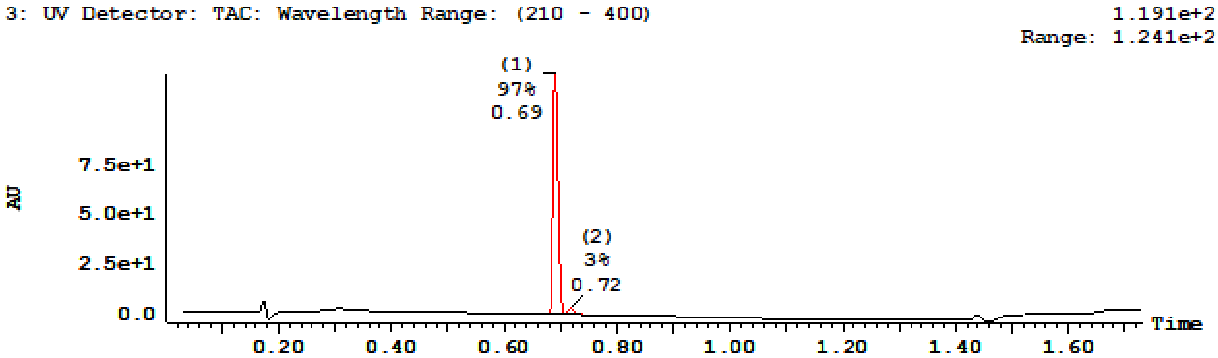

Peak Number	Compound	Time	AreaAbs	Area %Total	Width	Height	Mass Found
1		0.69	1e+006	97.23	0	1e+008	Not Found
2		0.72	4e+004	2.77	0	3e+006	Not Found

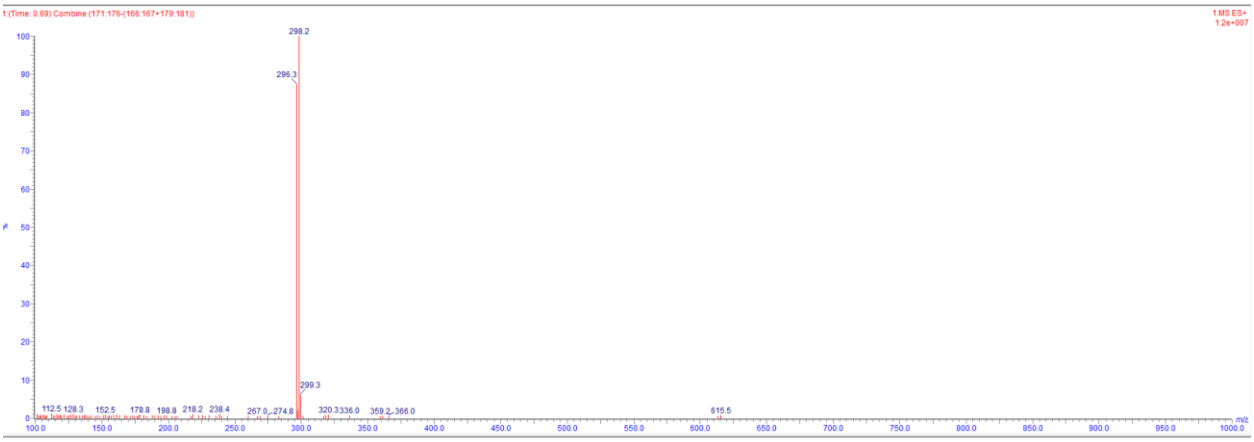

Compound **53b**

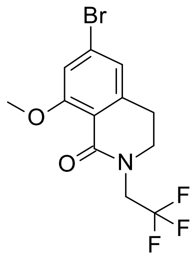

Exact Mass: 336.99
Molecular Formula: $C_{12}H_{11}BrF_3NO_2$

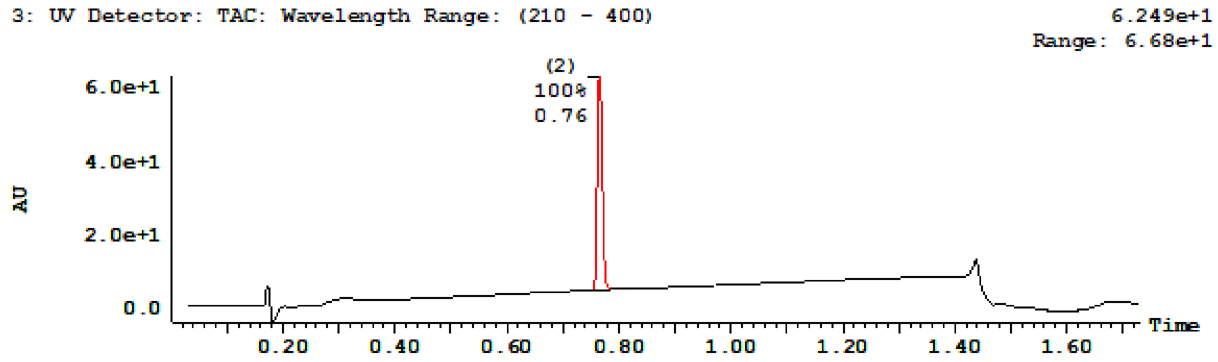

Peak Number	Compound	Time	AreaAbs	Area %Total	Width	Height	Mass Found
2		0.76	6e+005	100.00	0	6e+007	Not Found

Peak ID	Compound	Time	Mass Found
2		0.76	Not Found

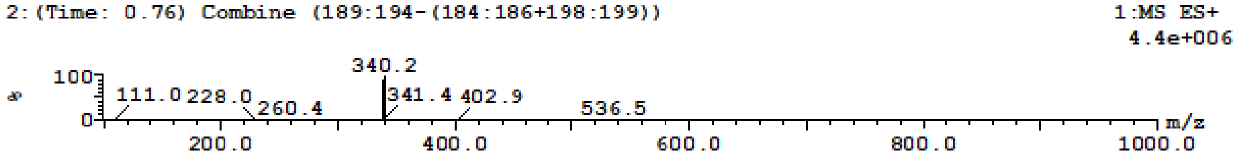

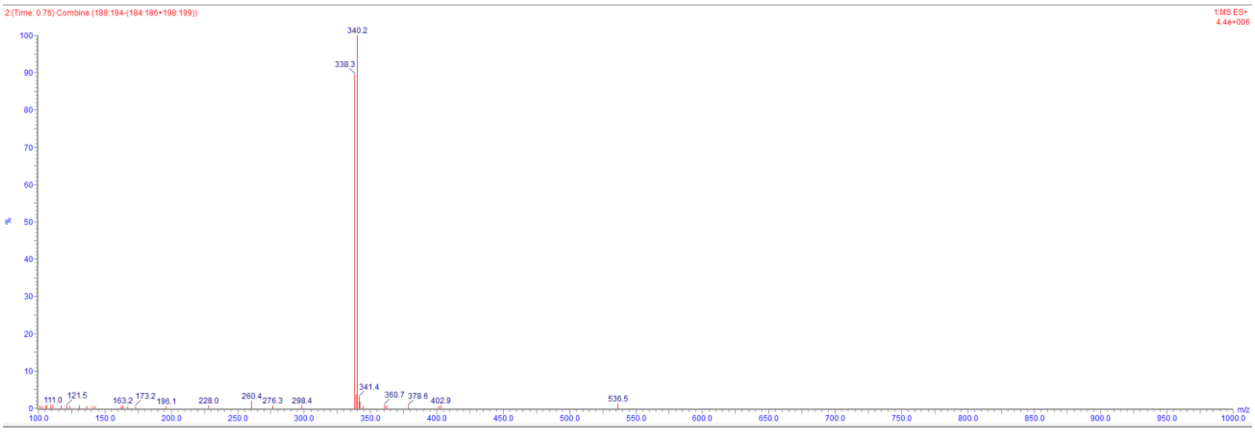

Compound **55a**

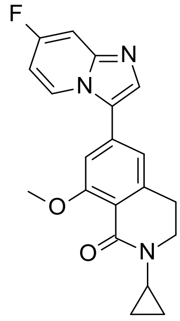

Exact Mass: 351.14
Molecular Formula: $C_{20}H_{18}FN_3O_2$

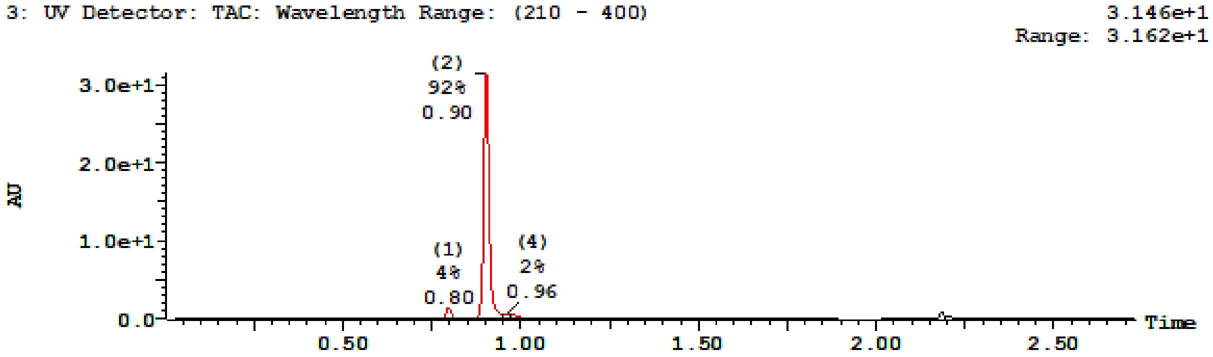

Peak Number	Compound	Time	AreaAbs	Area %Total	Width	Height	Mass Found
1		0.80	2e+004	3.76	0	1e+006	Not Found
2		0.90	6e+005	92.25	0	3e+007	Not Found

Peak ID	Compound	Time	Mass Found
2		0.90	Not Found

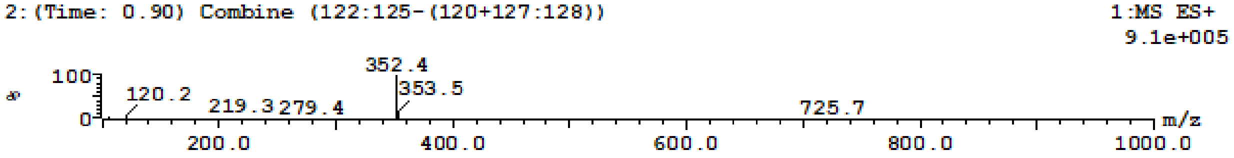

Compound **55b**

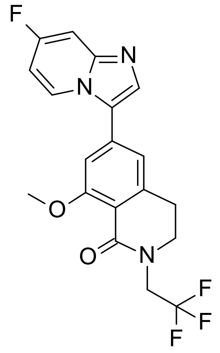

Exact Mass: 393.11
Molecular Formula: $C_{19}H_{15}F_4N_3O_2$

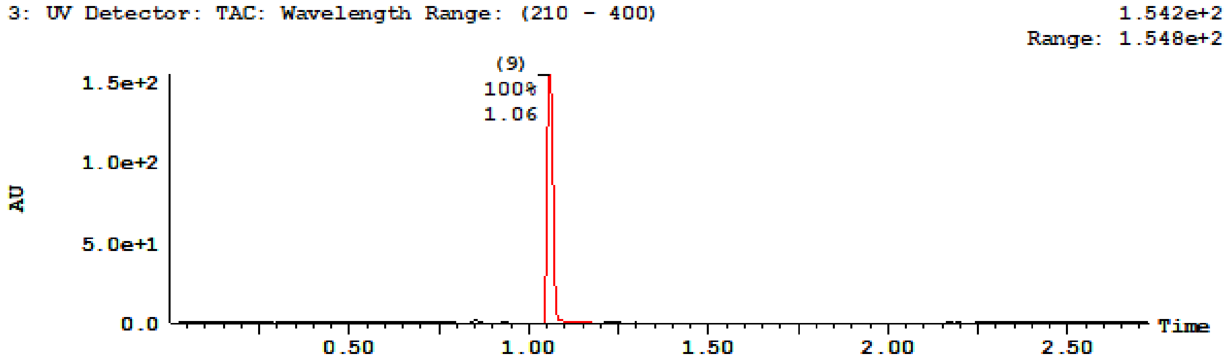

Peak Number	Compound	Time	AreaAbs	Area %Total	Width	Height	Mass Found
9		1.06	3e+006	100.00	0	2e+008	Not Found

Peak ID	Compound	Time	Mass Found
9		1.07	Not Found

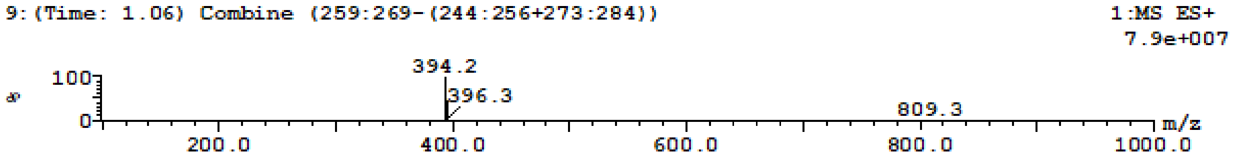

NMR Traces.

Compounds **8** and **9** have been previously described by Temal-Laib et al.[1].

Compound **10**

^{1}H NMR (400 MHz, DMSO-d_6)

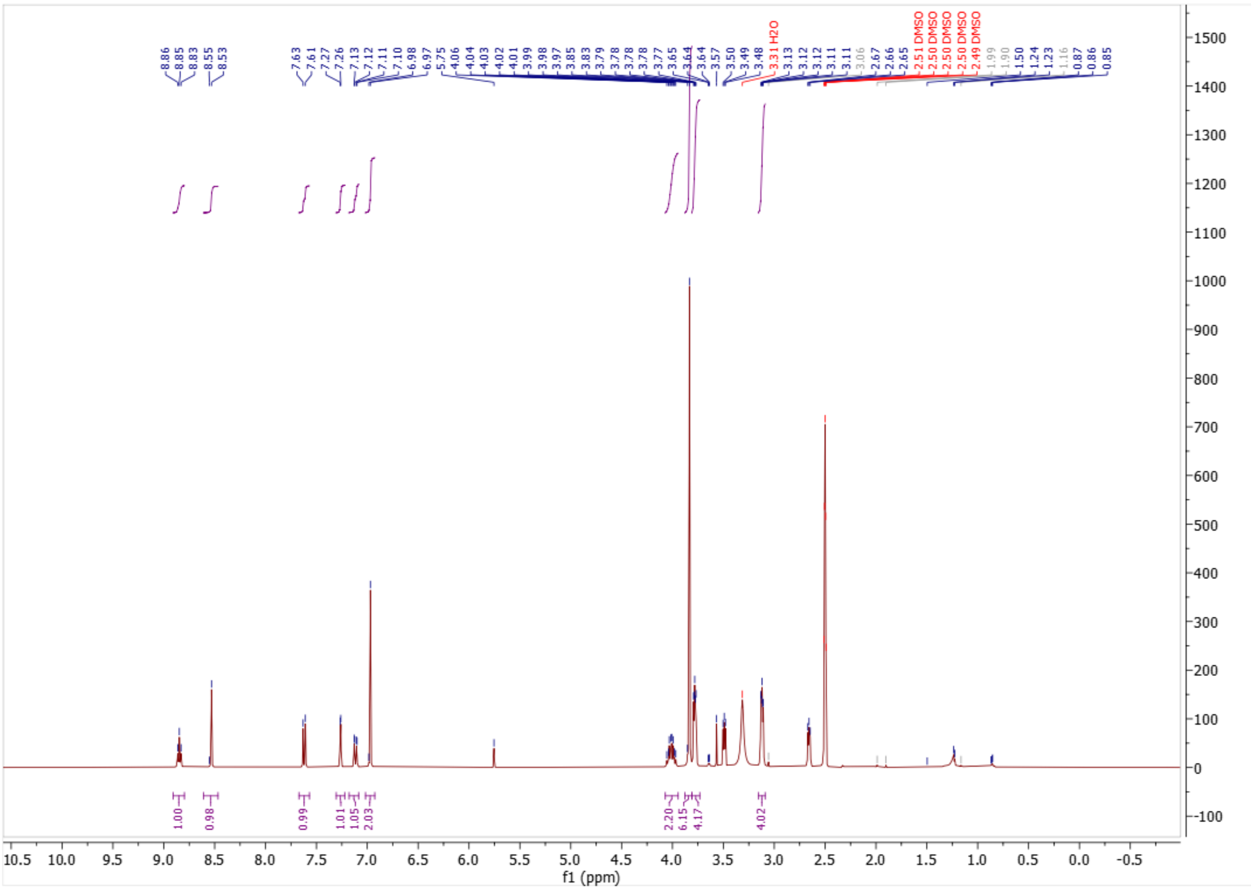

Compound **11**

^{1}H NMR (400 MHz, Chloroform-*d*)

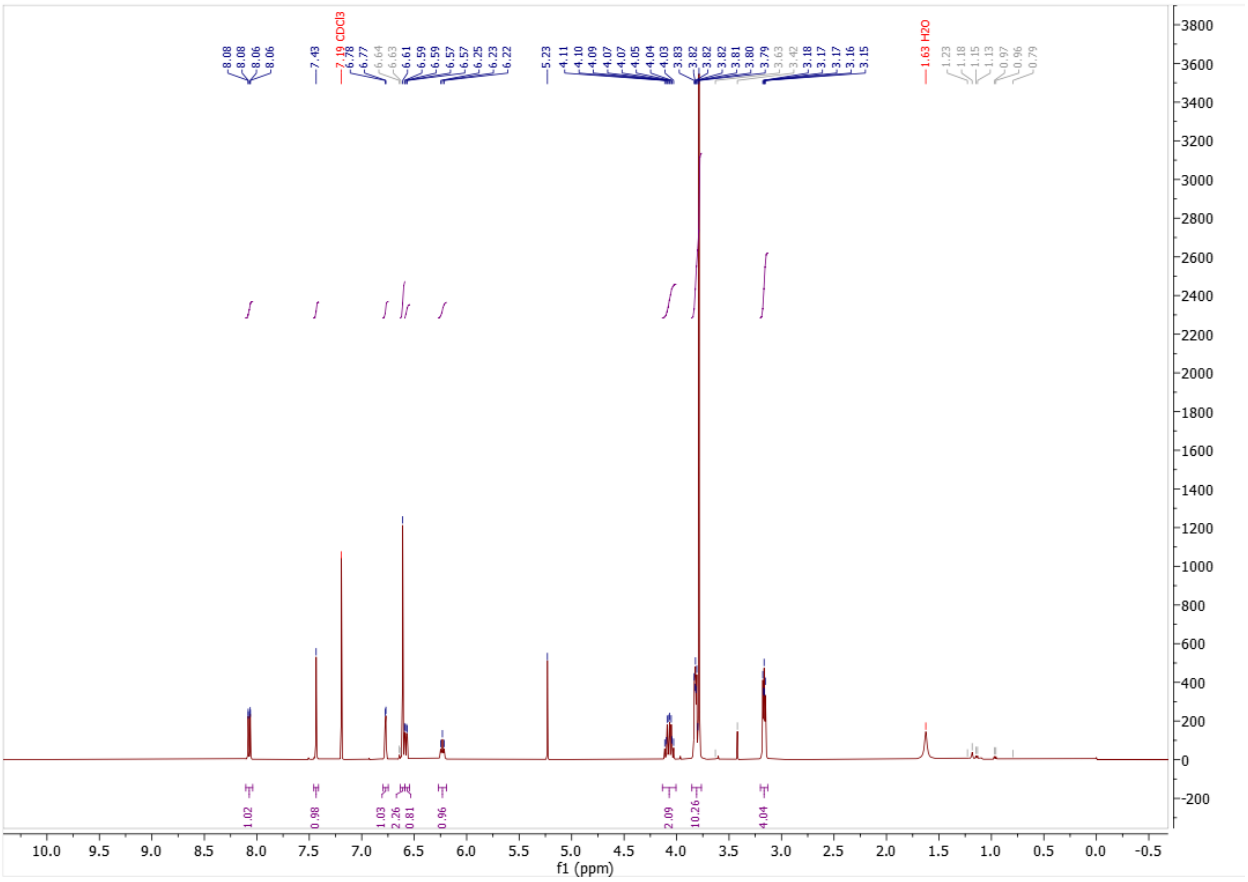

Compound **12**

^{1}H NMR (400 MHz, Chloroform-*d*)

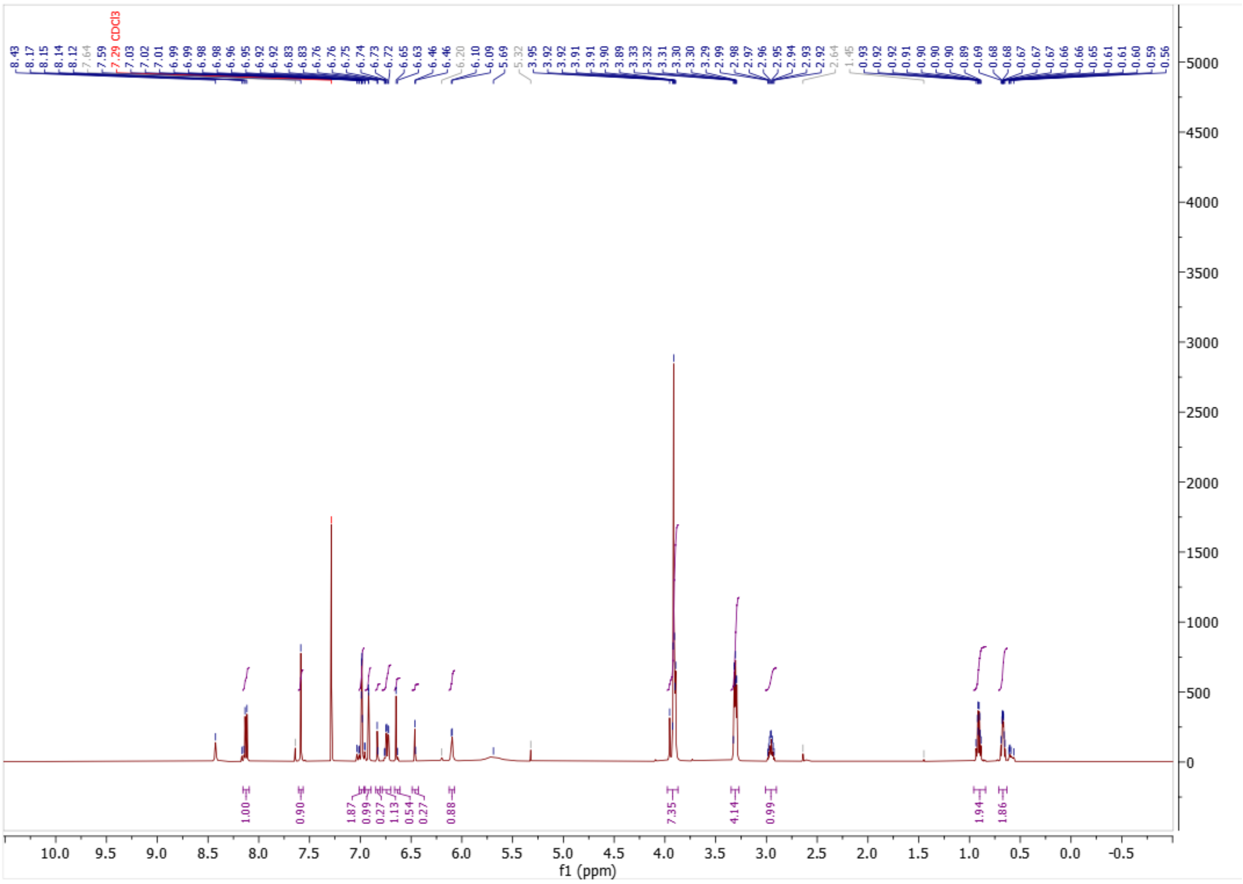

Compound **13**

^{1}H NMR (400 MHz, Chloroform-*d*)

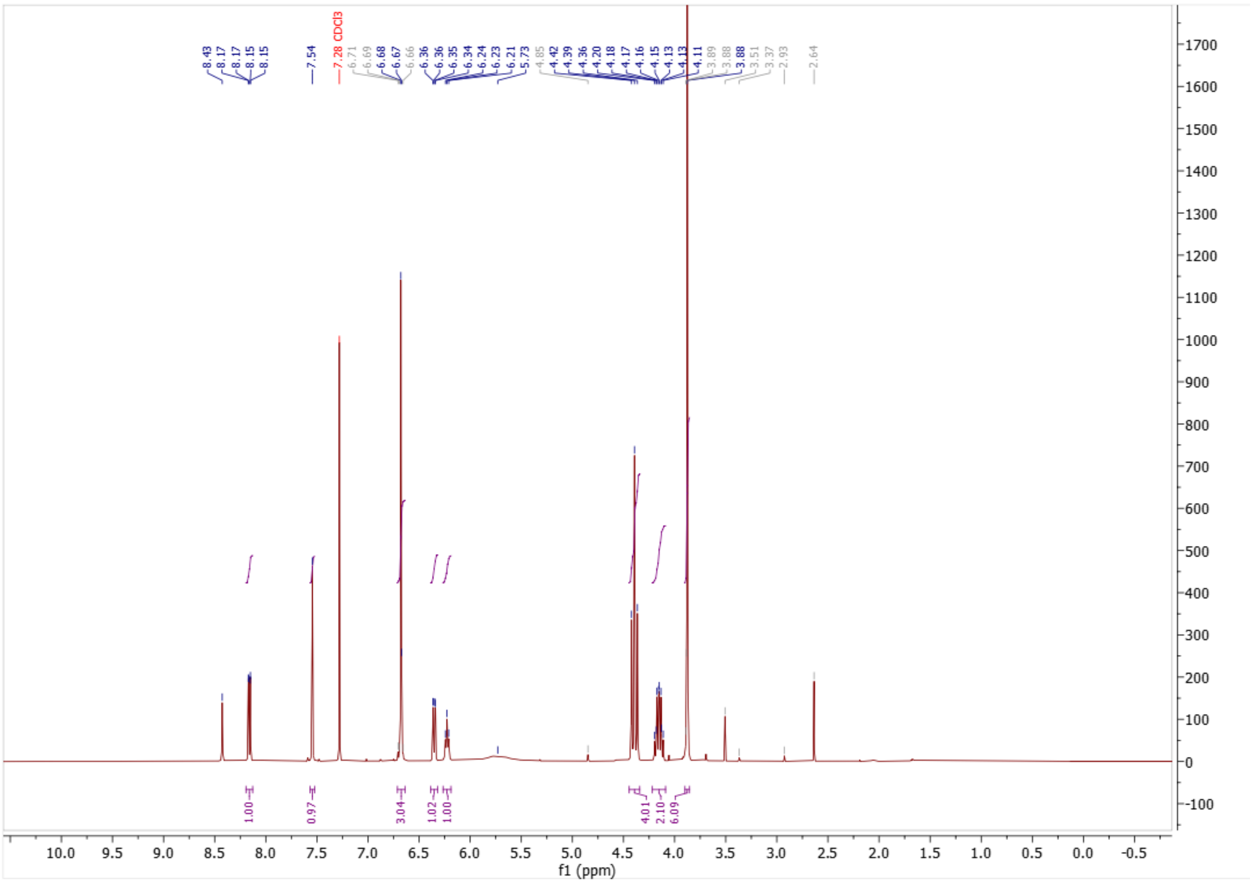

Compound **14**

^{1}H NMR (400 MHz, DMSO-d_6)

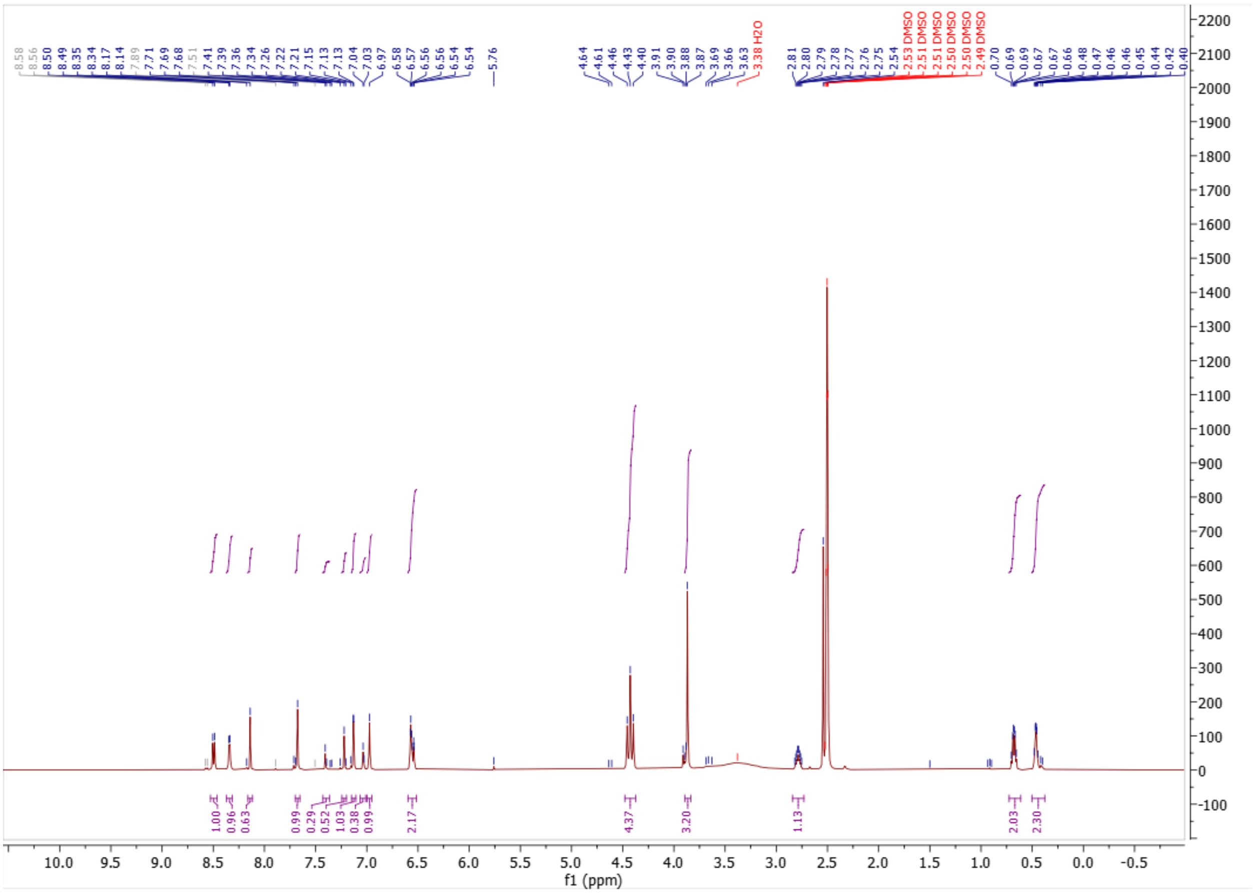

Compound **15**

^1H NMR (400 MHz, CD_3OD)

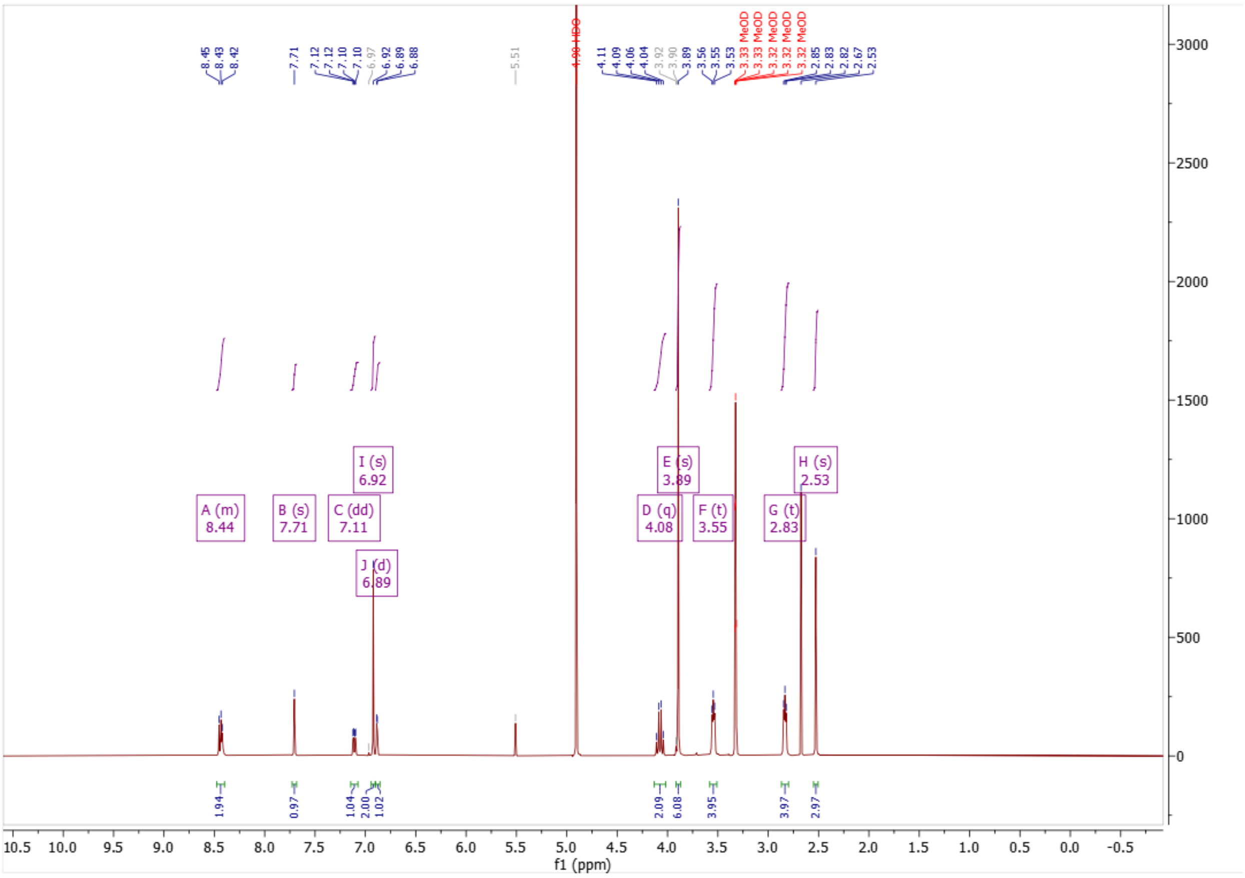

Compound **16**

^1H NMR (400 MHz, CD_3OD)

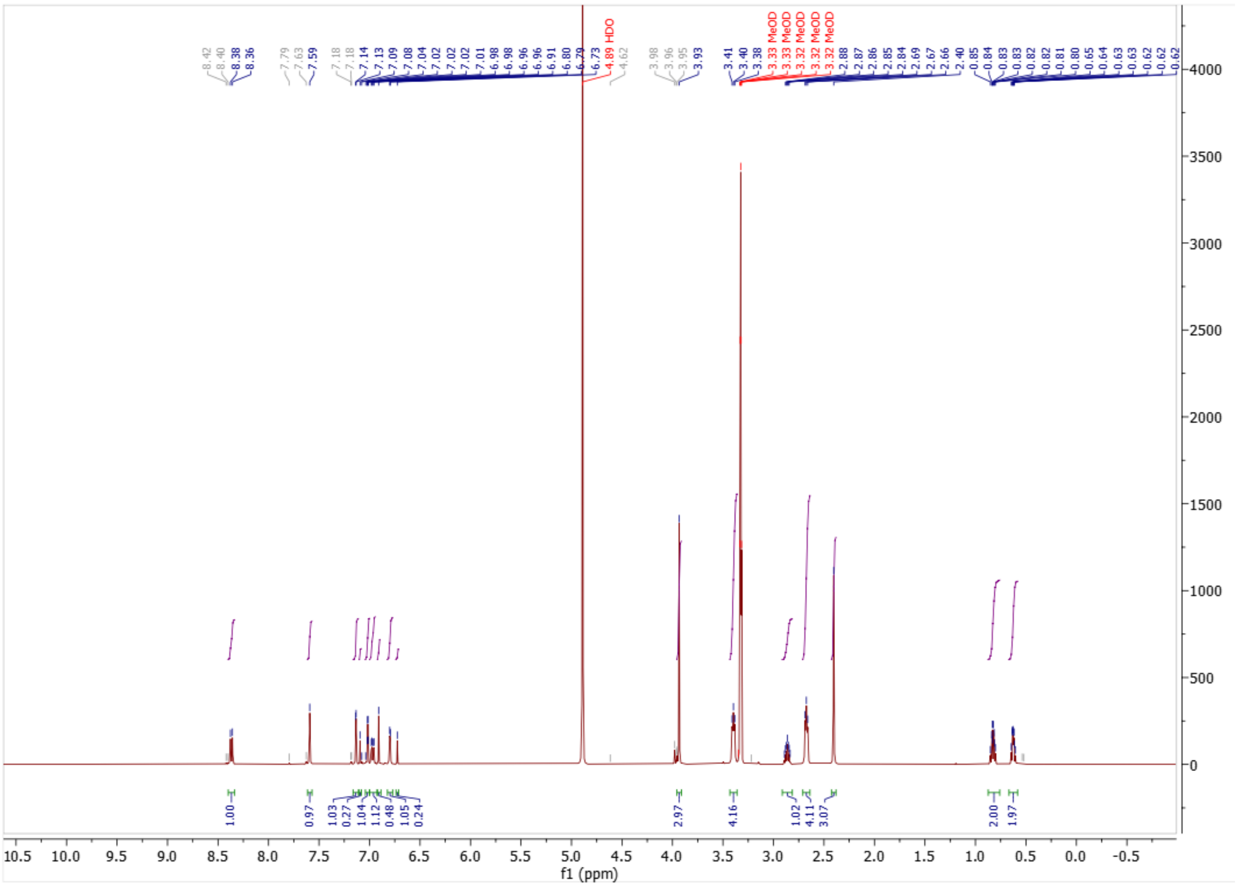

Compound **17**

^{1}H NMR (400 MHz, Chloroform-*d*)

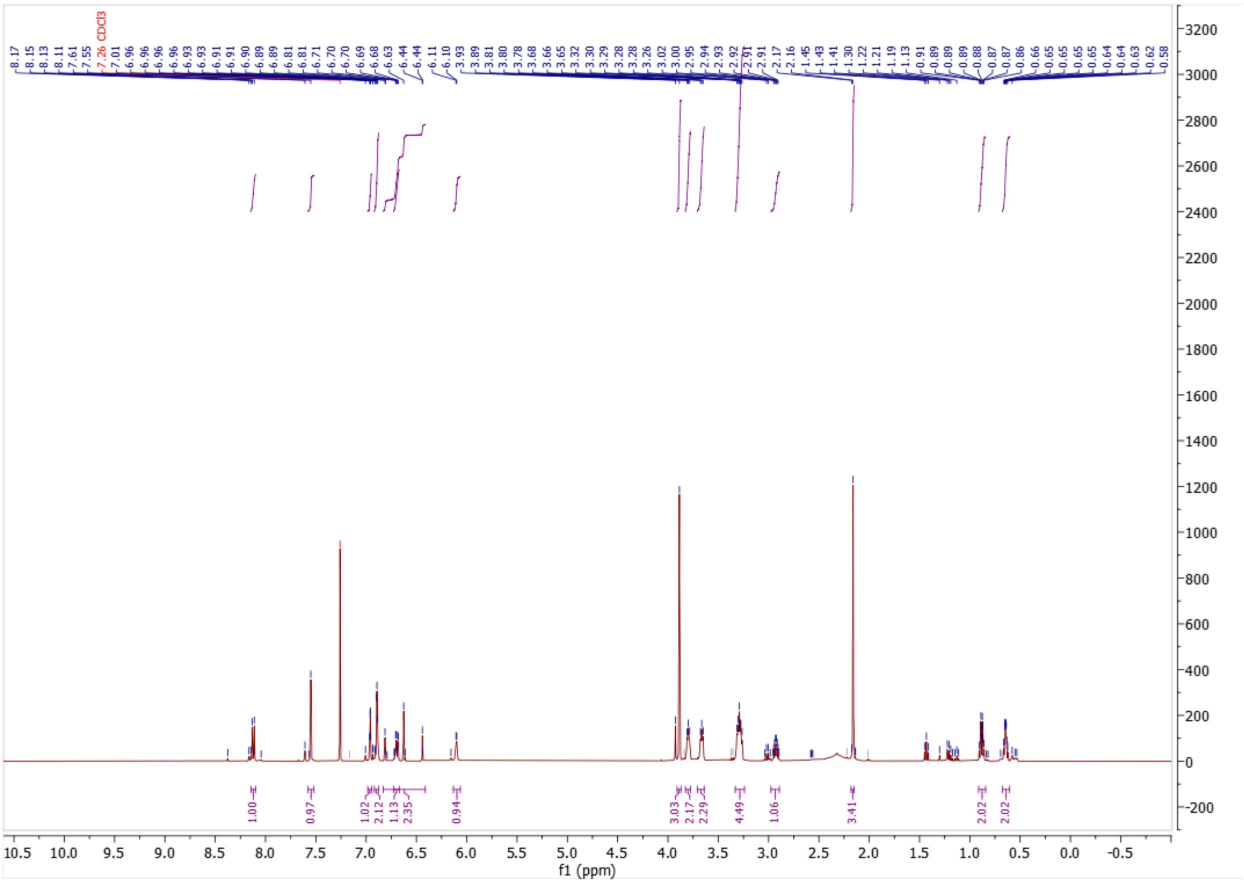

Compound **18**

^{1}H NMR (400 MHz, DMSO-d_6)

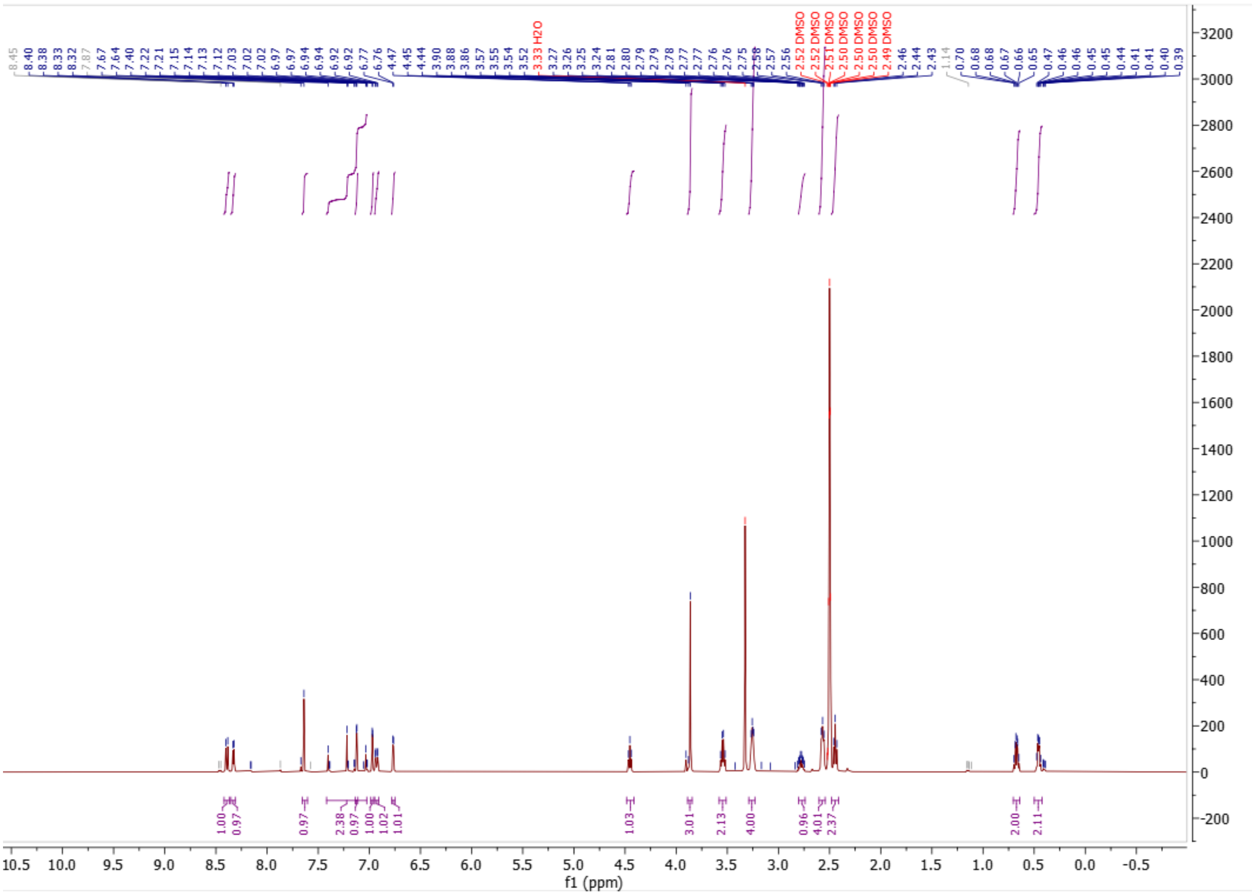

Compound **19**

^{1}H NMR (400 MHz, DMSO-d_6)

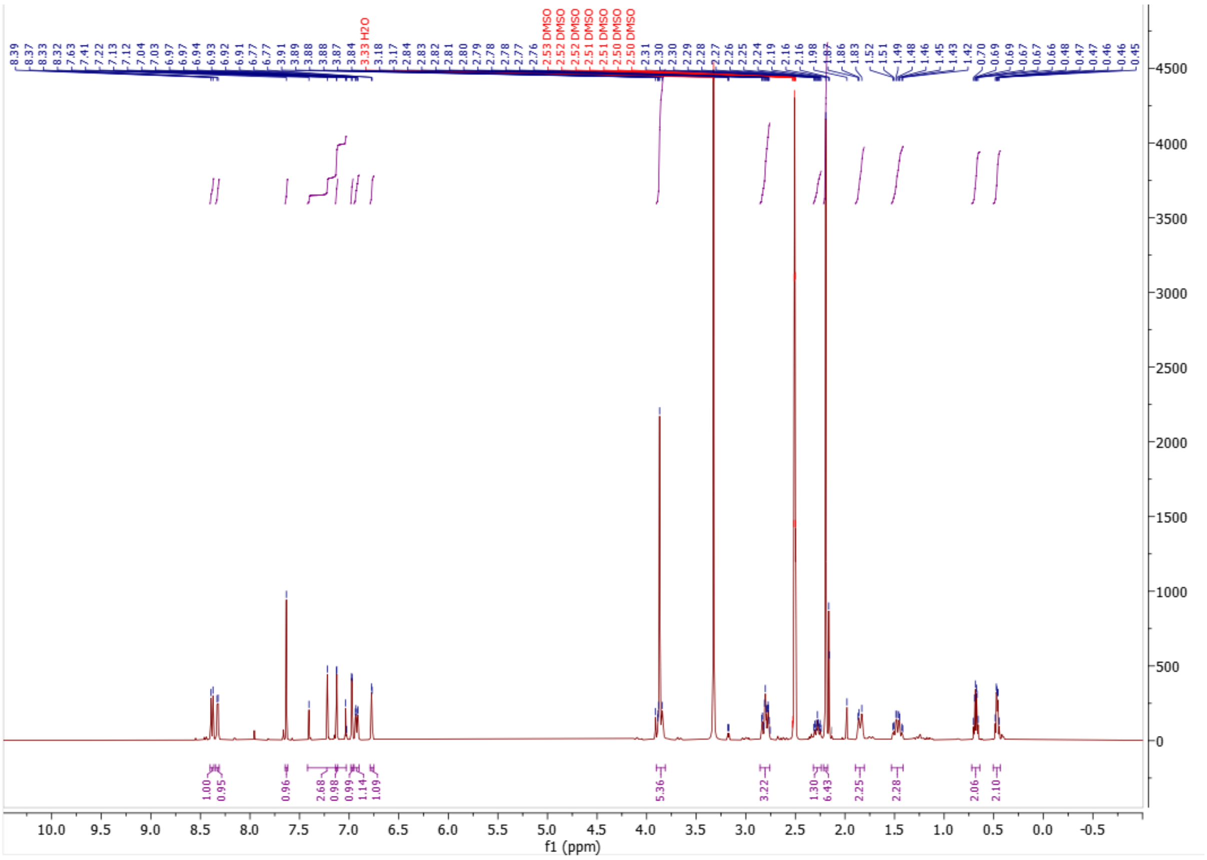

Compound **20**

^{1}H NMR (400 MHz, CD_3OD)

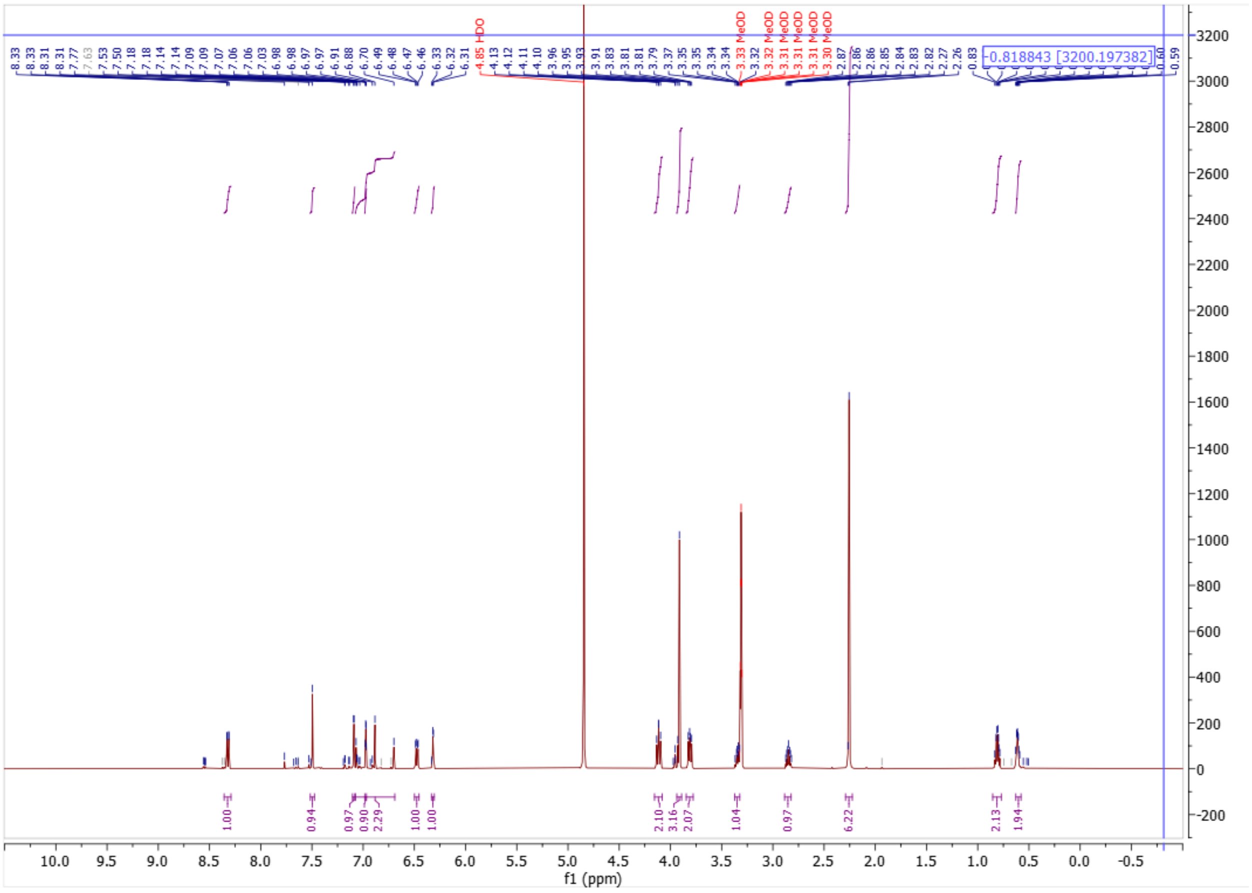

Compound **21**

^{1}H NMR (400 MHz, DMSO-d_6)

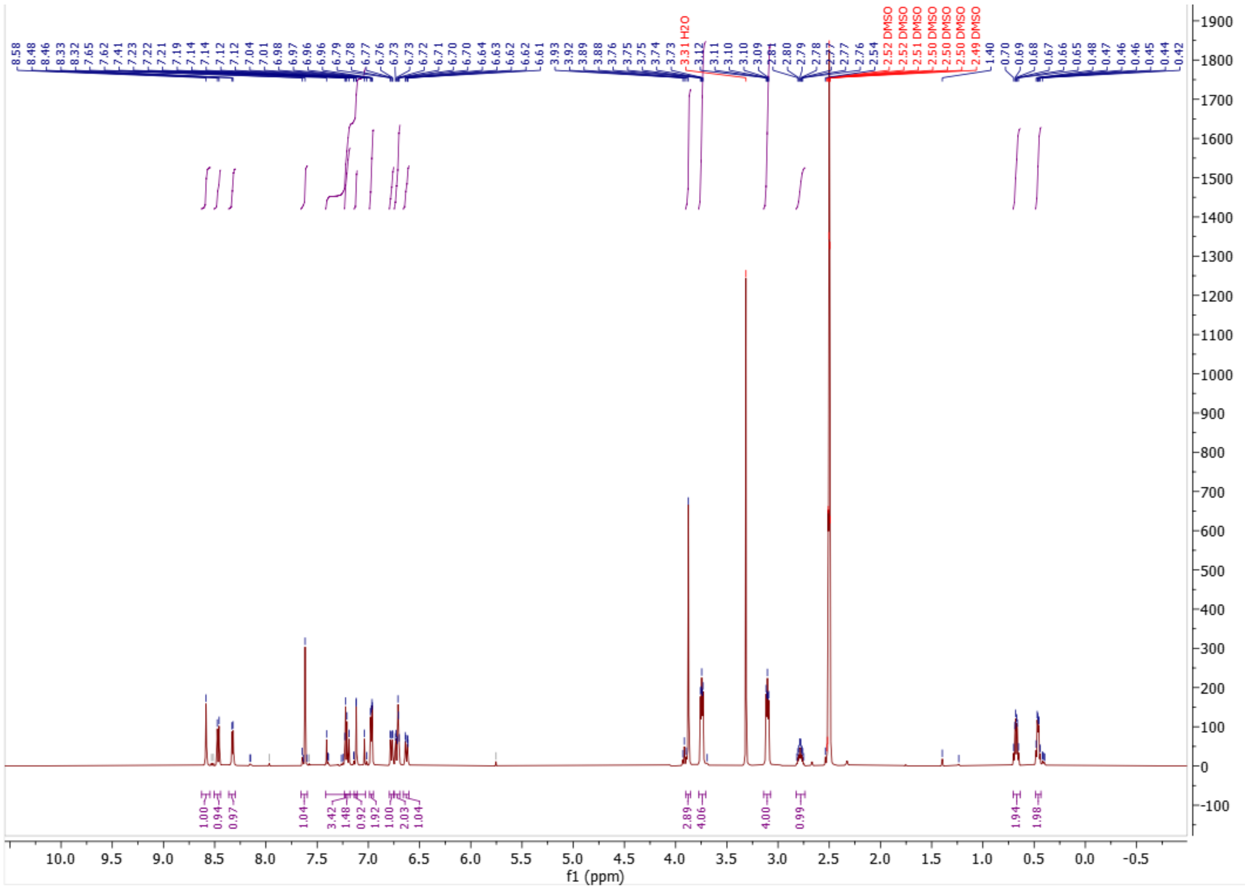

Compound **22**

^{1}H NMR (400 MHz, DMSO-d_6)

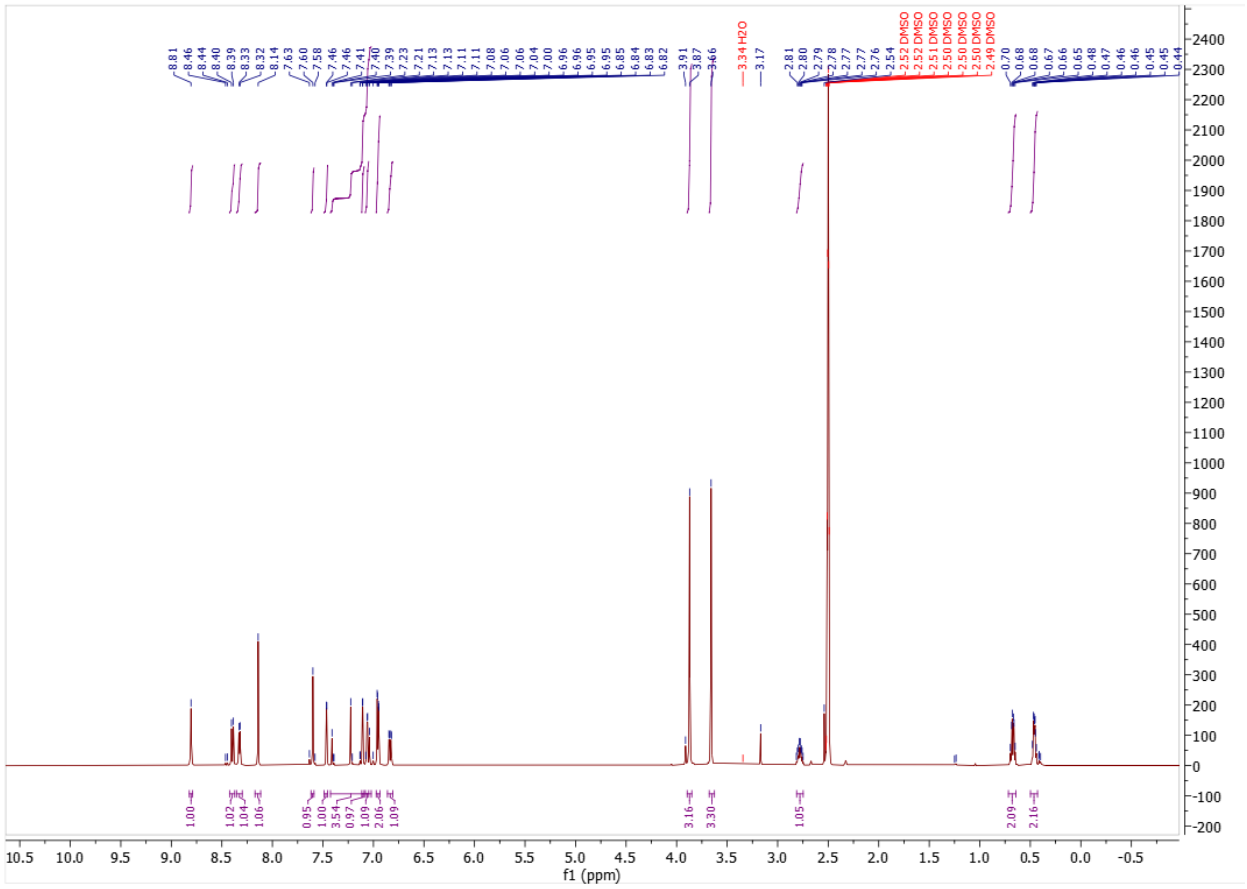

Compound **23**

^{1}H NMR (400 MHz, DMSO-d_6)

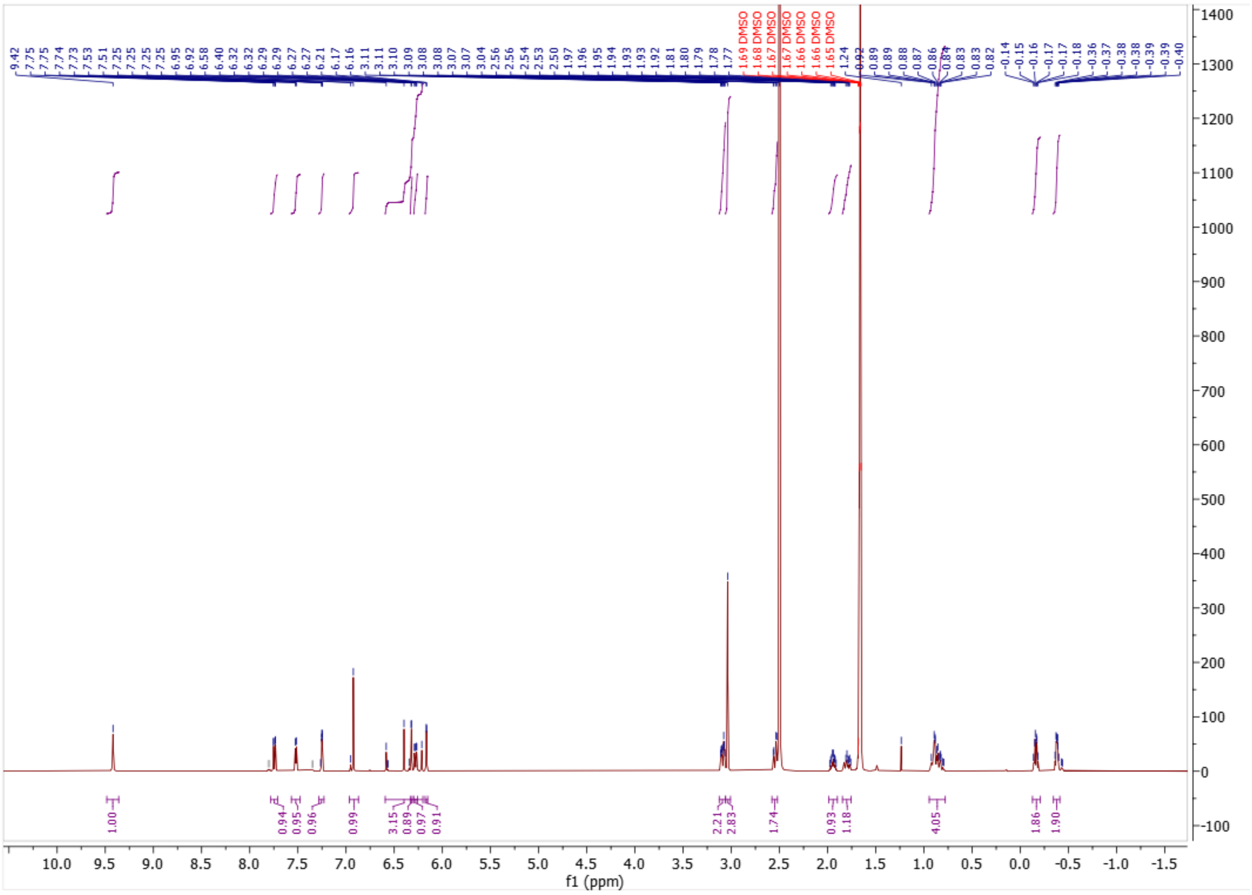

Compound **24**

^1H NMR (400 MHz, CD_3OD)

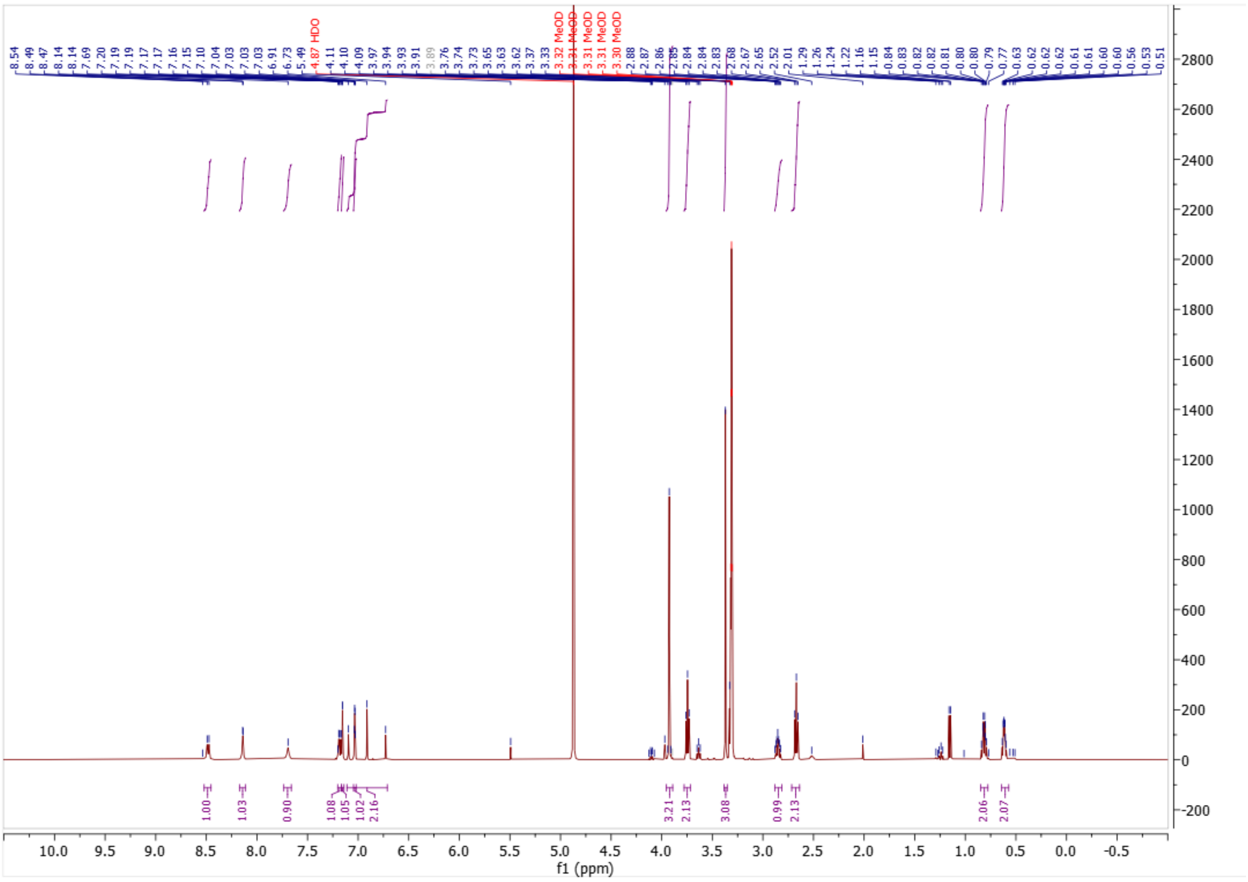

Compound **25**

^{1}H NMR (400 MHz, DMSO-d_6)

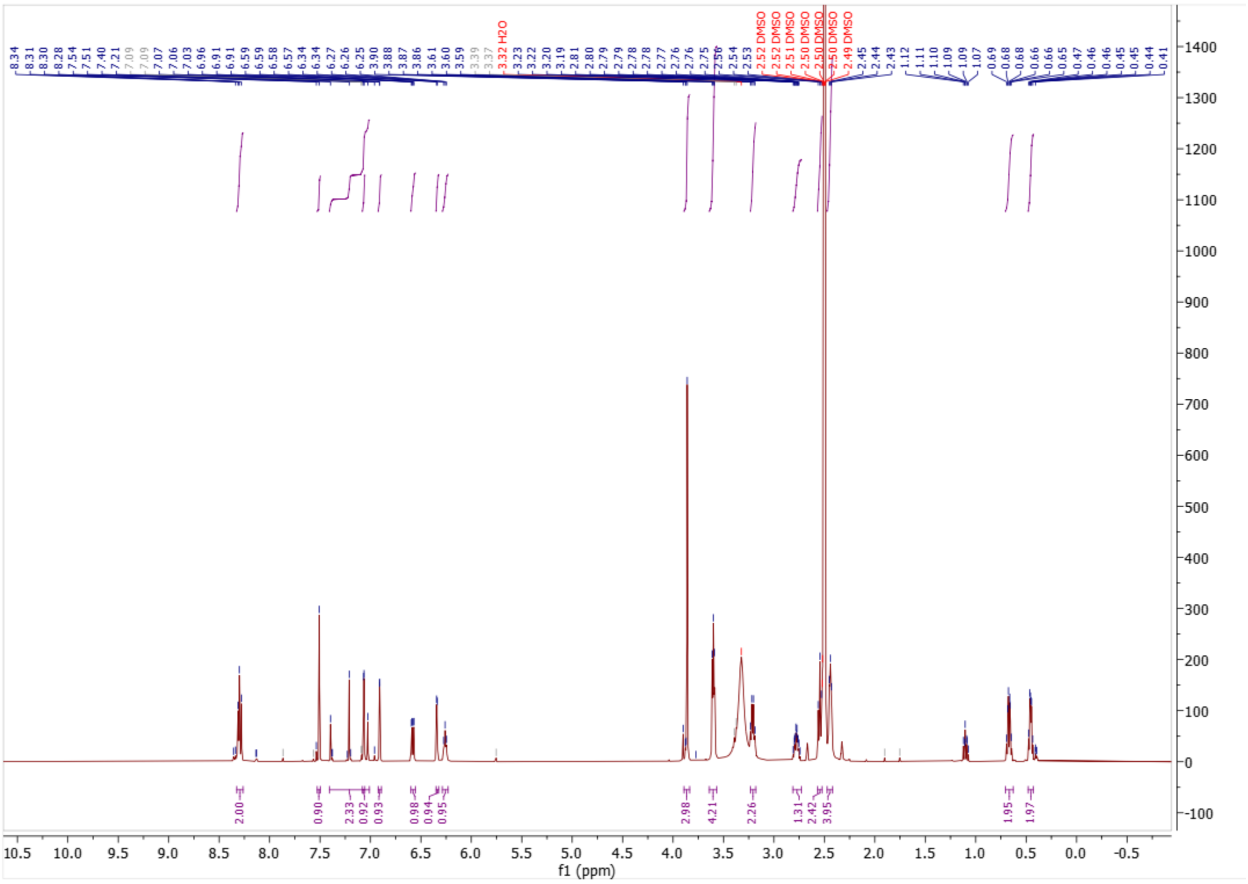

Compound **26**

^{1}H NMR (400 MHz, Chloroform-*d*)

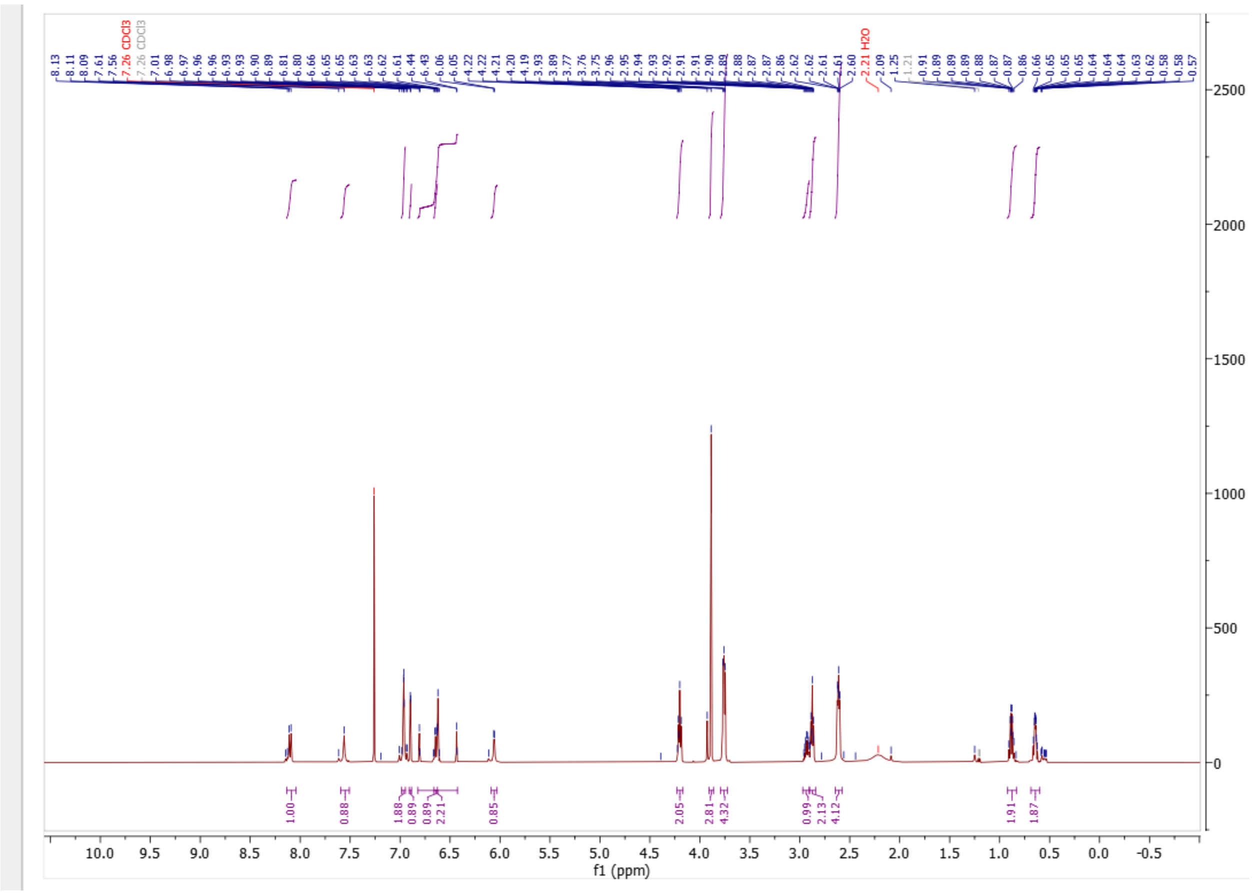

Compound **27**

^{1}H NMR (400 MHz, DMSO-d$_6$)

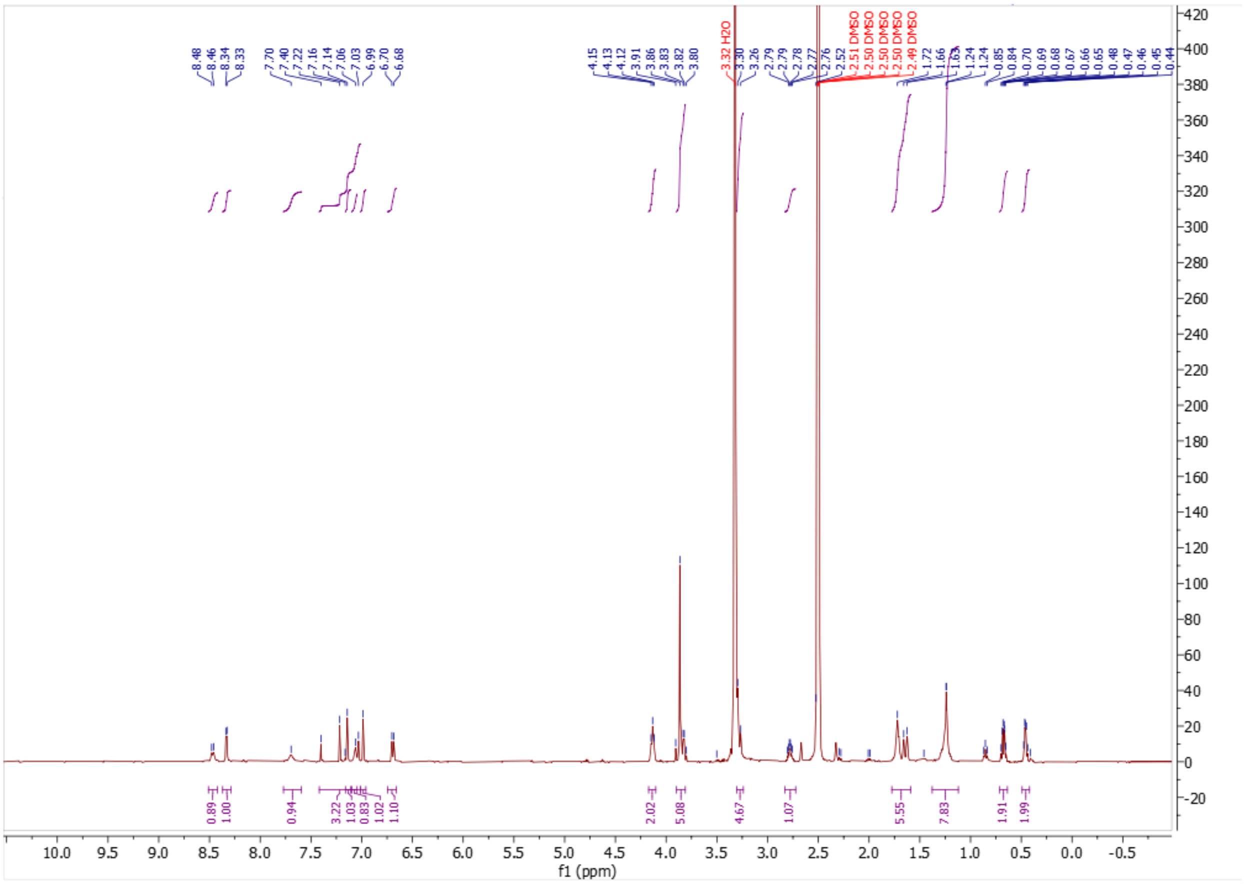

Compound **28**

^{1}H NMR (400 MHz, Methanol-d_4)

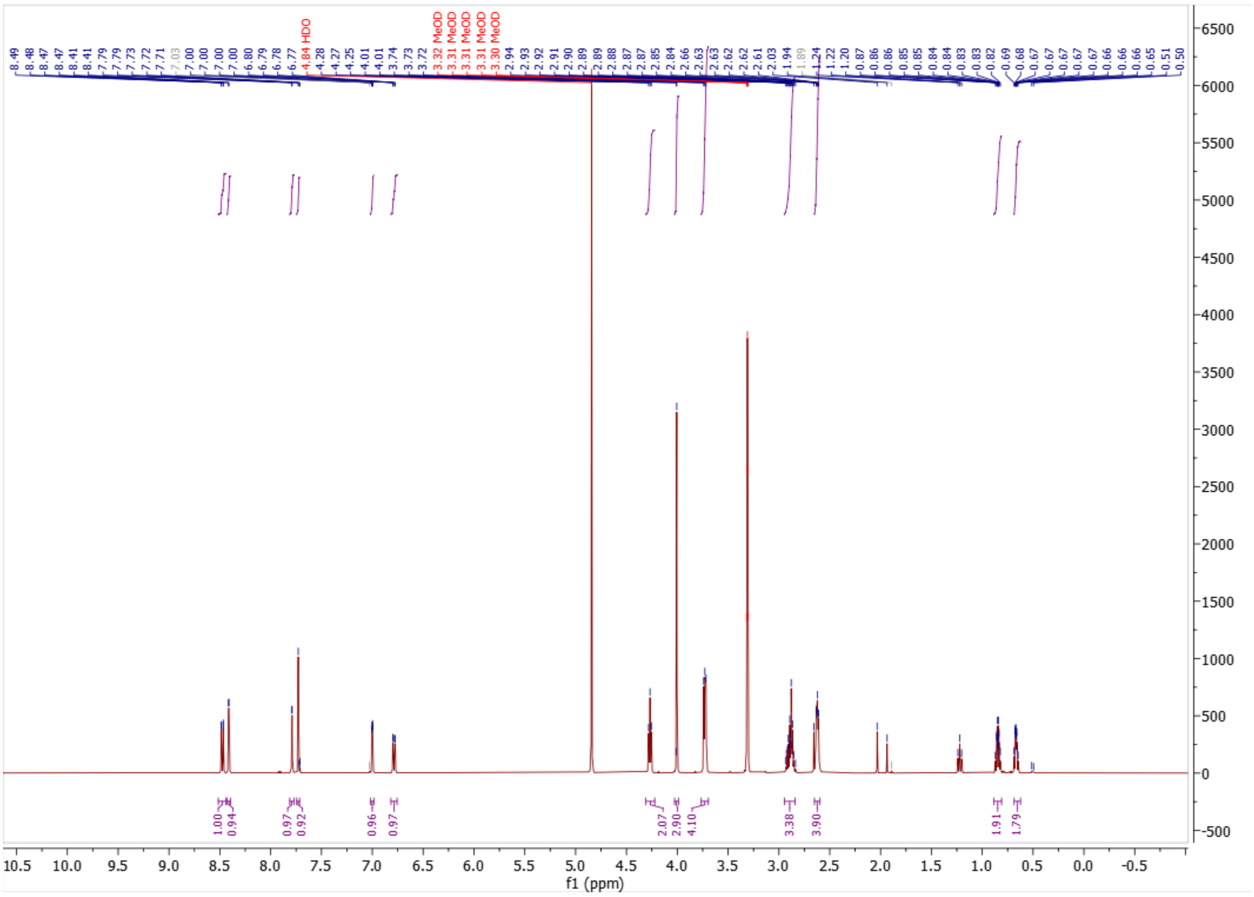

Compound **29**

^{1}H NMR (400 MHz, CD_3OD)

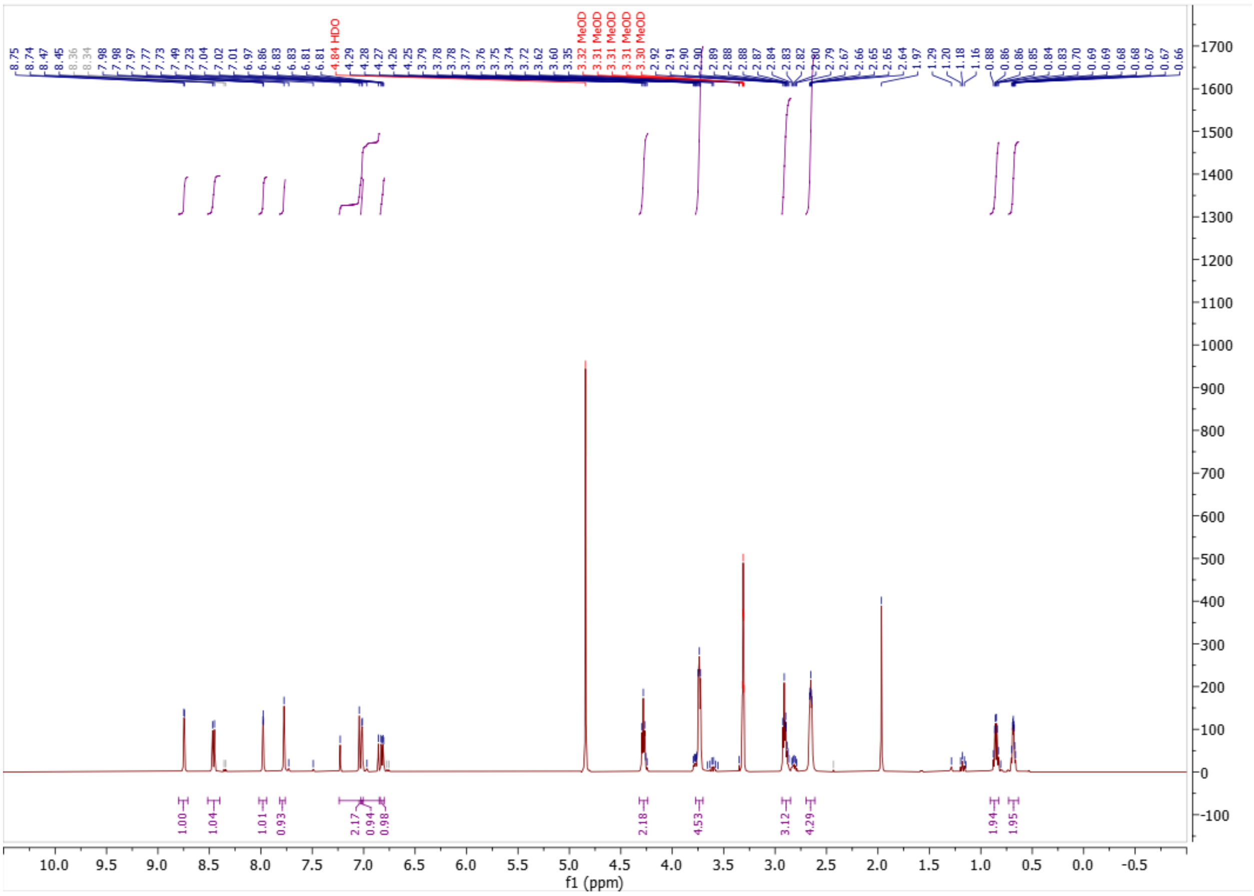

Compound **30**

^{1}H NMR (400 MHz, CD_3OD)

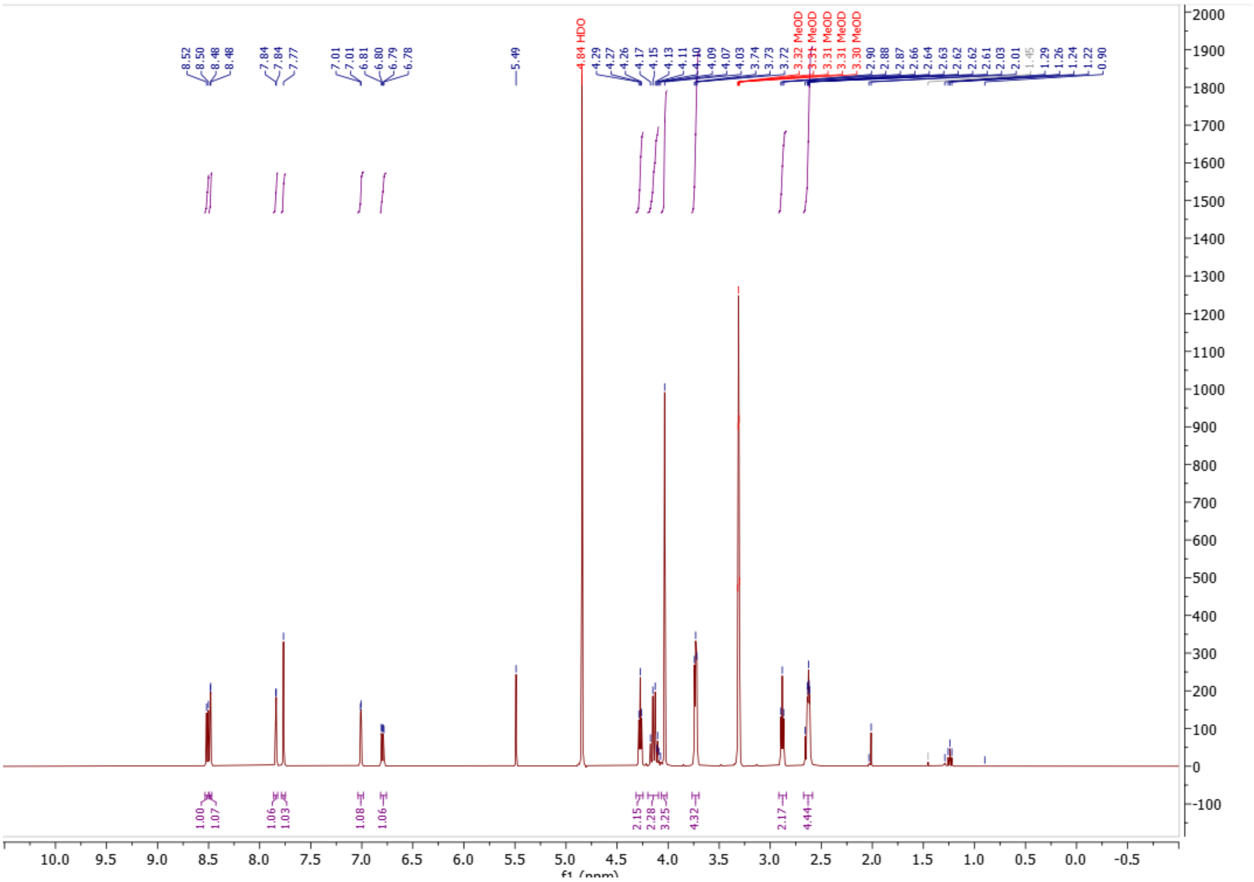

Compound **31**

^{1}H NMR (400 MHz, CD_3OD)

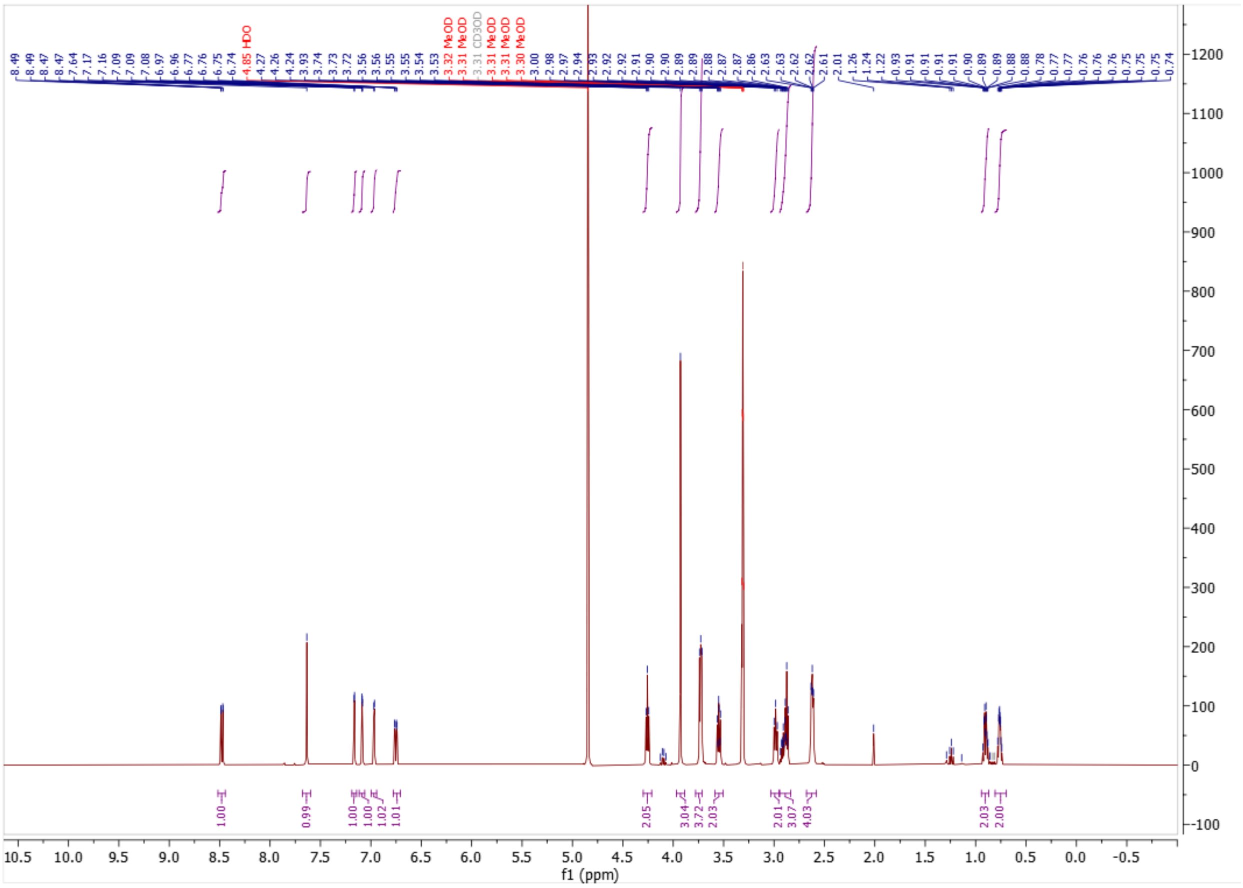

Compound **32**

^{1}H NMR (400 MHz, Methanol-*d4*)

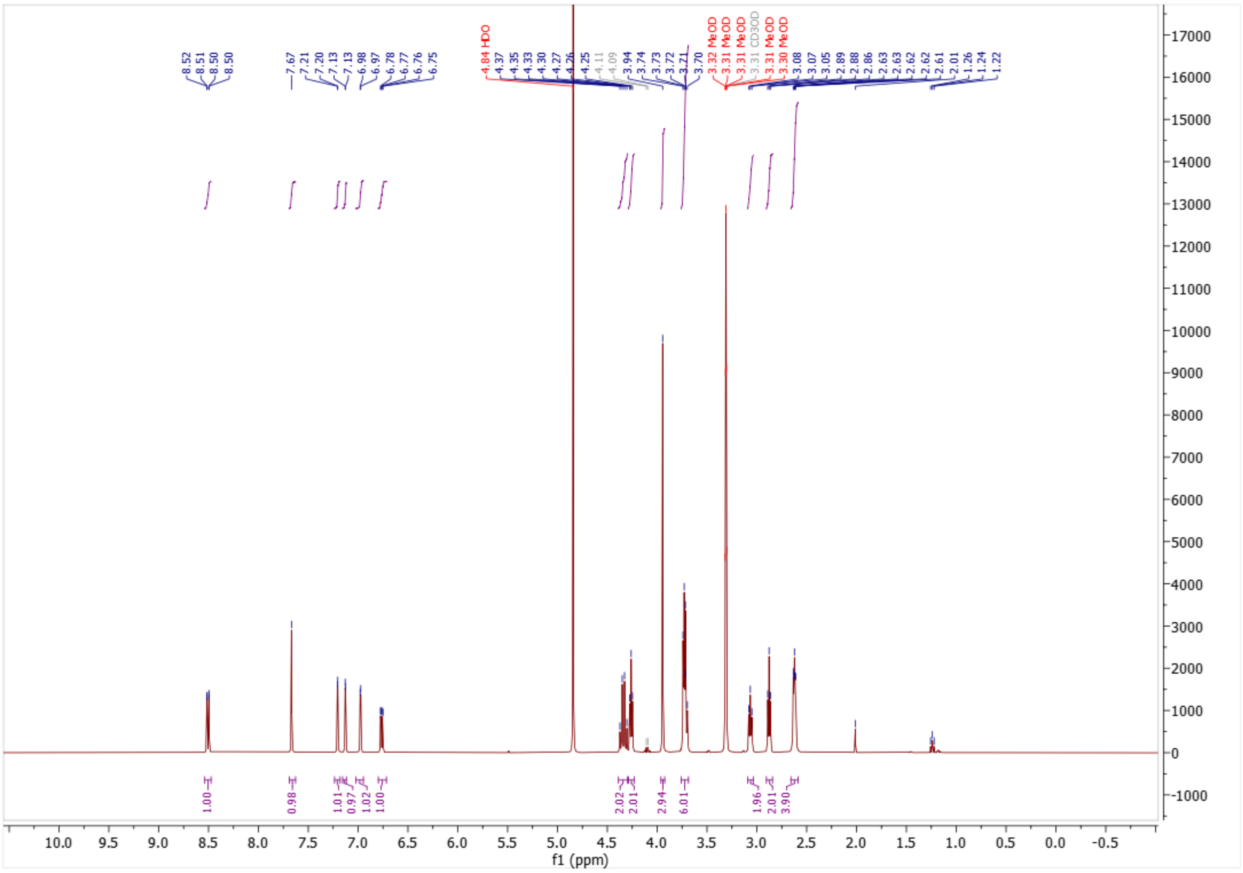

Supplementary Reference

(1) Temal-Laib, T.; Peixoto, C.; Desroy, N.; De Lemos, E.; Bonnaterre, F.; Bienvenu, N.; Picolet, O.; Sartori, E.; Bucher, D.; Lopez-Ramos, M.; Roca Magadan, C.; Laenen, W.; Flower, T.; Mollat, P.; Bugaud, O.; Touitou, R.; Pereira Fernandes, A.; Lavazais, S.; Monjardet, A.; Borgonovi, M.; Gosmini, R.; Brys, R.; Amantini, D.; De Vos, S.; Andrews, M. Optimization of Selectivity and Pharmacokinetic Properties of Salt-Inducible Kinase Inhibitors that Led to the Discovery of Pan-SIK Inhibitor GLPG3312. *J Med Chem* **2024**, *67* (1), 380-401. DOI: 10.1021/acs.jmedchem.3c01428
